# Mitochondrial retrograde signaling initiates HIF-1α/BNIP3/NIX-mediated mitophagy in Tibetan high-altitude adaptation

**DOI:** 10.1038/s41420-025-02933-8

**Published:** 2026-01-06

**Authors:** Yang Wei, Dayan Sun, Fei Wu, Shixuan Zhang, Bowen Cai, Yanyun Ma, Hongxiang Zheng, Xiangguang Shi, Yi Li, Shiguan Le, Xiang Zhou, Li Jin, Jiucun Wang

**Affiliations:** 1 State Key Laboratory of Genetic and Development of Complex Phenotypes, School of Life Sciences & Human Phenome Institute, Fudan University, Shanghai, 200438 China; 2 Department of Neonatal Surgery, Beijing Children’s Hospital, Capital Medical University, National Center for Children’s Health, Beijing, 100045 China; 3 Academy for Engineering & Technology, Fudan University, Shanghai, 200433 China; 4 Department of Dermatology, Huashan Hospital, Fudan University, Shanghai, 200040 China; 5 Research Unit of Dissecting the Population Genetics and Developing New Technologies for Treatment and Prevention of Skin Phenotypes and Dermatological Diseases (2019RU058), Chinese Academy of Medical Sciences, Beijing, 210042 China

**Keywords:** Mitophagy, Stress signalling

## Abstract

Genome-wide studies have identified the nuclear gene *EPAS1* and the mitochondrial M9a haplogroup as pivotal contributors to hypoxia adaptation in Tibetans. However, the interaction between these two genetic components is not yet clear. In this study, we demonstrate that cells harboring the Tibetan-specific M9a haplogroup with downregulated *EPAS1* (M9a+sh*EPAS1*) exhibit enhanced cellular function under hypoxic conditions. These cells display improved mitochondrial function and proliferation, alongside reduced apoptosis and mtDNA-mediated inflammation, driven by the activation of HIF-1α-BNIP3/NIX-mediated mitophagy and an increase in reactive oxygen species (ROS) levels. Furthermore, treatment with N-acetylcysteine (NAC), PX-478, or Mdivi-1 significantly attenuated BNIP3/NIX-mediated mitophagy, leading to an aggravation of mtDNA-mediated inflammation and apoptosis in M9a+sh*EPAS1* cells during hypoxia. This study first reveals that ROS-driven HIF-1α-BNIP3/NIX-mediated mitophagy mitigates hypoxia-induced inflammation and apoptosis, contributing to the enhanced hypoxia adaptation observed in Tibetans. HIF-1α-BNIP3/NIX-mediated mitophagy may offer potential therapeutic targets for high-altitude illnesses by regulating cellular energy metabolism and inflammation.

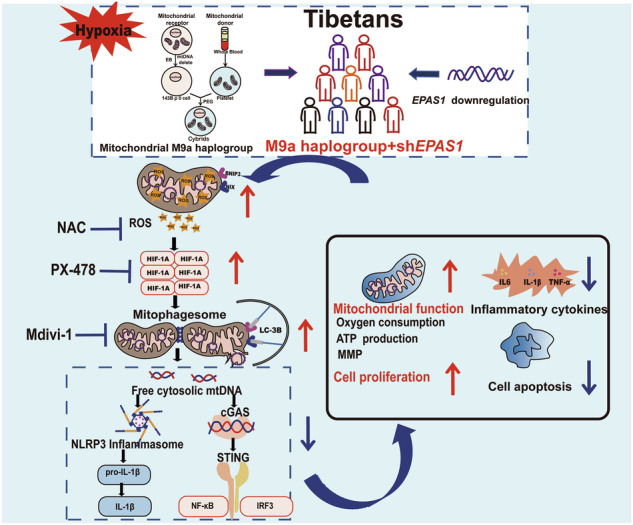

## Introduction

The extreme hypoxic challenge of the Tibetan Plateau has driven remarkable physiological adaptations in Tibetans over millennia, positioning them as an unparalleled model for decoding the genetic architecture of hypoxia tolerance [1]. Genome-wide studies have established *EPAS1* (encoding HIF-2α) and *EGLN1* (encoding PHD2) as cornerstone adaptive loci within the hypoxia-inducible factor (HIF) pathway [2, 3]. Tibetan-enriched *EPAS1* (*HIF-2A*) variants reach ~80% frequency at high altitudes but reach rare frequencies of less than 5% in lowlands [4, 5]. Mechanistically, these single-nucleotide polymorphisms (SNPs) within intronic regions of *EPAS1* reduce mRNA expression by diminishing promoter activity, thereby contributing to lower hemoglobin concentrations in Tibetan populations [6, 7]. Similarly, altitude-correlated *EGLN1* variants diminish HIF-2α protein abundance, further associating with reduced hemoglobin levels and inflammatory responses [8–10]. Notably, heterozygous *EPAS1* knockout mice recapitulate the Tibetan phenotype of attenuated hemoglobin elevation and confer protection against chronic hypoxic injury [11], providing direct experimental validation for our focus on *EPAS1* downregulation in Tibetan high-altitude adaptation.

Complementary to these nuclear genetic adaptations, mitochondria, as central hubs for energy metabolism and redox balance, are essential for hypoxic survival [12]. Studies suggested that mitochondrial haplogroups, defined by characteristic mitochondrial DNA (mtDNA) variations, represent evolutionary adaptations potentially underlying Tibetan high-altitude adaptation [13–15]. Notably, the M9a haplogroup is highly prevalent in Tibetans (25%) but rare or absent in lowland populations, indicating its selective advantage [16, 17]. However, current models predominantly emphasize nuclear genetic factors, leaving a critical gap in understanding mitochondrial-nuclear co-evolution and its integrated contribution to the adaptive phenotype.

Research found that mitochondrial components, such as mtDNA, NAD^+^, Ca^2+^, and reactive oxygen species (ROS), modulate nuclear gene expression via mitochondrial retrograde signaling during cellular stress, mitochondrial dysfunction, or hypoxia [18, 19]. Critically, ROS primarily generated at Complexes I and III of the mitochondrial electron transport chain (ETC) directly regulate the HIF signaling pathway [20–23]. This crosstalk is epitomized by hypoxia-inducible factor-1α (HIF-1α), a master regulator of BNIP3/NIX-mediated mitophagy that drives adaptation in hypoxia-related diseases [24, 25]. Based on converging evolutionary and mechanistic evidence—encompassing mitochondrial centrality in hypoxic response, natural selection of the Tibetan-enriched mitochondrial DNA haplogroup M9a, and bidirectional ROS-HIF regulatory crosstalk—we propose a mechanistic hypothesis that mitochondrial retrograde signaling orchestrates nuclear genomic reprogramming through modulation of HIF-responsive elements to establish hypoxia-tolerant phenotypes.

In the present study, we generated Tibetan-enriched M9a cytoplasmic hybrids (cybrids) and lowland-associated M7/8 cybrids using the trans-mitochondrial cybrid method in mtDNA-depleted 143B (TK⁻) ρ⁰ osteosarcoma cells, and then generated a stable *EPAS1* knockdown cell line in M9a cybrids using lentiviral transduction to recapitulate the nuclear and mitochondrial adaptive genotype of the Tibetan population. This model was used to investigate the molecular mechanism of Tibetan hypoxia adaptation, with a focus on mito-nuclear interactions under chronic hypoxia exposure, and uncover novel therapeutic targets for altitude-related pathologies.

## Results

### Tibetan-specific M9a haplogroup enhances mitochondrial function under hypoxia

**Fig. 1:**
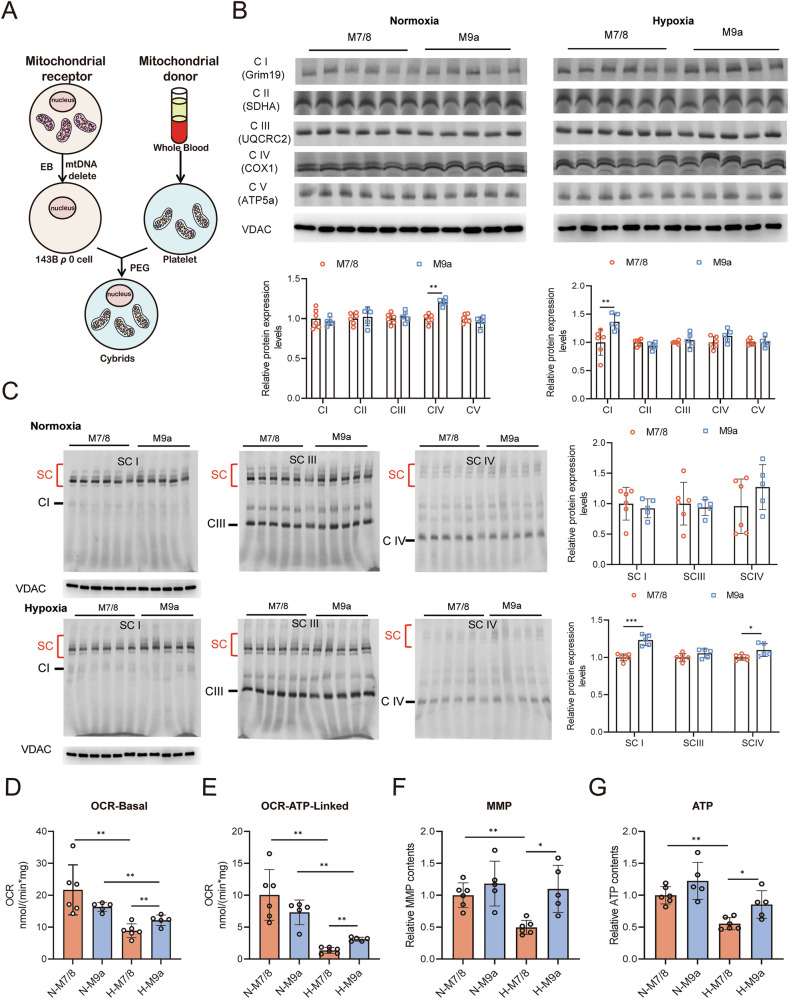
The M9a cybrids exhibited enhanced mitochondrial function than the M7/8 cybrids under hypoxic condition. **A** Schematic diagram of trans-mitochondrial cell model (EB Ethidium Bromide, PEG Polyethylene Glycol). **B** Mitochondrial respiratory complexes were assessed in M7/8 (*n* = 6) and M9a (*n* = 5) cybrids under normoxic and hypoxic conditions. Complexes I, II, III, IV, and V were immunoblotted using anti-Grim19, SDHA, UQCRC2, COX1, and ATP5a antibodies, with VDAC serving as the loading control. Protein levels were normalized to those in M7/8 cybrids. CI Complex I, CII Complex II, CIII Complex III, CIV Complex IV, CV Complex V. **C** Mitochondrial respiratory supercomplexes were evaluated in M7/8 (*n* = 6) and M9a (*n* = 5) cybrids under normoxic and hypoxic conditions. Supercomplexes I, III, and IV were immunoblotted using anti-Grim19, UQCRC2, and COX1 antibodies, with VDAC as the loading control. Protein levels were normalized to those in M7/8 cybrids. SCI Supercomplex I, SCIII Supercomplex III, SCIV Supercomplex IV. **D**, **E** Mitochondrial respiratory capacity was measured in M7/8 (*n* = 6) and M9a (*n* = 5) cybrids under normoxic and hypoxic conditions. Basal oxygen consumption rate (OCR) represents total endogenous respiration, and ATP-linked OCR was calculated by subtracting the value after the addition of oligomycin (2.5 mM) from the total endogenous respiration value. **F**, **G** MMP and ATP levels were determined in M7/8 (*n* = 6) and M9a (*n* = 5) cybrids under normoxic and hypoxic conditions. MMP and ATP levels in M9a cybrids were normalized to those in M7/8 cybrids. All cybrids were cultured under normoxic (21% O₂) or hypoxic (1% O₂) conditions for 48 h. Data are presented as the mean ± SD of three independent experiments. Statistical analysis was performed using two-tailed, unpaired Student’s *t* tests; *P < 0.05; **P < 0.01; ***P < 0.001.

Our previous studies, as well as those of others, have consistently demonstrated that the M9a haplogroup is the most prevalent mitochondrial haplogroup among Tibetans [16, 17]. To investigate whether the M9a haplogroup confers a biochemical advantage under hypoxic conditions, we first selected the plain M7/8 haplogroups, which are sister branches of the M9a haplogroup, as controls to minimize the introduction of additional mutations. Tibetan-specific M9a cytoplasmic hybrids (cybrids) and plain-specific M7/8 cybrids were then generated using the mitochondrial cybrid method in 143B (TK^−^) ρ0 cells (Fig. 1A), and the entire mitochondrial genome of all cybrid cells was fully sequenced (Supplementary Tables S1–S11).

Mitochondrial haplogroups consist of a series of mtDNA mutations that have been extensively studied for their impact on mitochondrial respiratory chain function [26, 27]. The mitochondrial respiratory chain comprises five complexes—Complexes I, II, III, IV, and V—which together form the electron transport chain responsible for ATP generation. We hypothesized that mtDNA mutations within specific haplogroups might affect mitochondrial function by influencing the assembly of these respiratory chain complexes. To test this, we first evaluated the assembly levels of mitochondrial complexes in the cybrids. Our findings revealed that M9a cybrids exhibited significantly increased assembly levels of complex IV under normoxic conditions and complex I under hypoxic conditions (Fig. 1B). Moreover, these complexes can assemble into larger supercomplexes, such as I + III + IV, II + III + IV, and I + III, which enhance electron transfer efficiency and overall mitochondrial function. Therefore, we also assessed the assembly levels of these supercomplexes in all cybrids. We observed that the supercomplex assembly levels in M9a cybrids were significantly higher under hypoxic conditions compared to M7/8 cybrids (Fig. 1C).

Next, we measured the mitochondrial oxygen consumption rate (OCR) under both normoxic and hypoxic conditions in the cybrids, as OCR is a key indicator of mitochondrial function. Our results showed a significant reduction in both basal and ATP-linked OCR in all cybrids under hypoxia. Notably, the basal OCR increased by 37%, and the ATP-linked OCR by 120% in M9a cybrids compared to M7/8 cybrids under hypoxic conditions, reflecting a marked enhancement in ATP production efficiency (Fig. 1D, E). Additionally, we observed a significant decline in mitochondrial membrane potential (MMP) and ATP levels in M7/8 cybrids under hypoxia. In contrast, M9a cybrids exhibited a 120% increase in MMP and a 50% increase in ATP levels relative to M7/8 cybrids under hypoxic conditions, further corroborating the OCR results (Fig. 1F, G). These findings demonstrate that the Tibetan M9a haplogroup cybrids exhibit enhanced mitochondrial function, thereby facilitating adaptation to hypoxic environments.

### M9a^+^sh*EPAS1* cells promote mitochondrial function and inhibit cell apoptosis during hypoxia

**Fig. 2:**
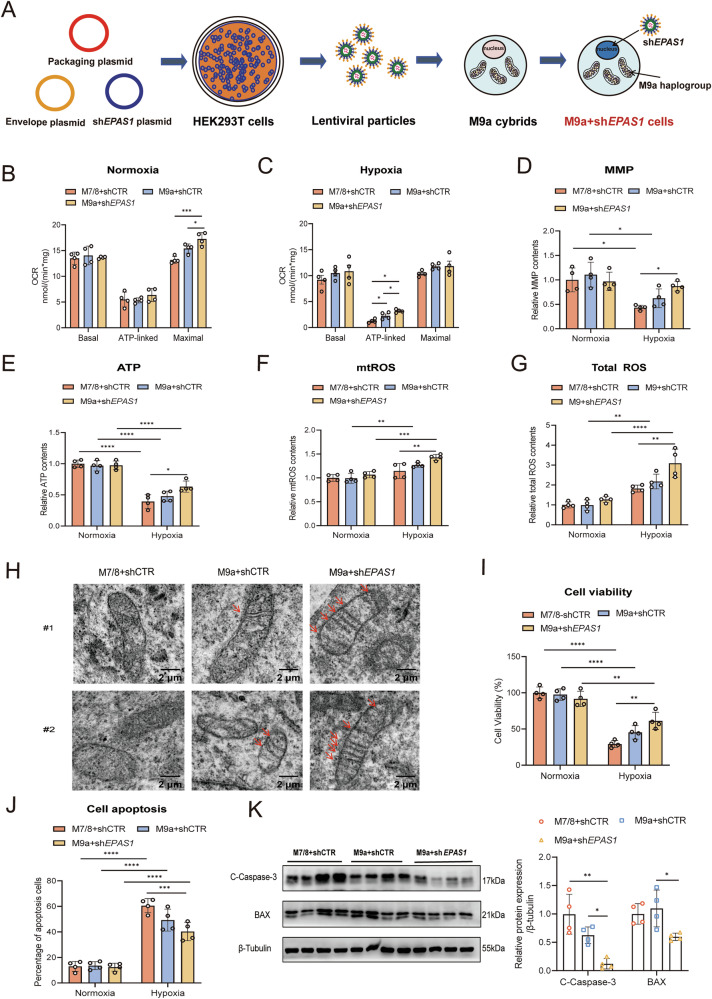
M9a+sh*EPAS1* cells exhibited optimal cellular function under hypoxic conditions. **A** Schematic diagram of constructing the M9a+sh*EPAS1* cell model. **B**, **C** Mitochondrial OCR was measured in M7/8+shCTR (*n* = 4), M9a+shCTR (*n* = 4), and M9a+sh*EPAS1* (*n* = 4) cells under normoxic and hypoxic conditions. Basal OCR represents total endogenous respiration; ATP-linked OCR was calculated by subtracting the value after oligomycin (2.5 mM) from total endogenous respiration; maximal OCR was assessed after the addition of the uncoupling agent FCCP (0.1 mM). **D**, **E** MMP and ATP content were determined in M7/8+shCTR (*n* = 4), M9a+shCTR (*n* = 4), and M9a+sh*EPAS1* (*n* = 4) cells under normoxic and hypoxic conditions. MMP and ATP levels in M9a+shCTR and M9a+sh*EPAS1* cells were normalized to those in M7/8+shCTR cells. **F**, **G** mtROS and total ROS contents were determined in the M7/8+shCTR (*n* = 4), M9a+shCTR (*n* = 4) and M9a+sh*EPAS1* (*n* = 4) cells under normoxic and hypoxic conditions. The mtROS and total ROS contents in the M9a+shCTR and M9a+sh*EPAS1* cells were normalized to those in the M7/8+shCTR cells. **H** Mitochondrial structure was analyzed in M7/8+shCTR (*n* = 2), M9a+shCTR (*n* = 2), and M9a+sh*EPAS1* (*n* = 2) cells using transmission electron microscopy (TEM) under hypoxic conditions. Scale bar: 2 μm. **I** Cell viability was assessed in M7/8+shCTR (*n* = 4), M9a+shCTR (*n* = 4), and M9a+sh*EPAS1* (*n* = 4) cells under normoxic and hypoxic conditions using the CCK-8 assay. **J** Cell apoptosis was determined in the M7/8+shCTR (*n* = 4), M9a+shCTR (*n* = 4) and M9a+sh*EPAS1* (*n* = 4) cells under normoxic and hypoxic conditions using Annexin V-FITC staining. **K** Western blot analysis of C-Caspase-3 and BAX protein levels in M7/8+shCTR (*n* = 4), M9a+shCTR (*n* = 4), and M9a+sh*EPAS1* (*n* = 4) cells under hypoxic conditions. Grayscale analysis of target protein levels was normalized to β-Tubulin. All cells were cultured under normoxia (21% O₂) or hypoxia (1% O₂) for 48 h. Data are presented as the mean ± SD of three independent experiments. **B**, **C**, **K** One-way ANOVA followed by the Tukey test; **D**–**F**, **G**, **I**, **J** Two-way analysis of variance (ANOVA) followed by the Tukey test. *P < 0.05; **P < 0.01; ***P < 0.001; ****P < 0.0001.

Previous studies have demonstrated that *EPAS1* exhibits the strongest selection signal and is downregulated in Tibetans [3–5]. To mimic the nuclear adaptive genotype of the Tibetan population, we generated a stable *EPAS1* knockdown cell line in M9a cybrids using lentiviral transduction (Fig. 2A). The efficiency of the knockdown was confirmed by mRNA and protein analyses, as compared to scrambled shRNA control (shCTR) cells (Supplementary Fig. S1A, B). Mitochondrial function was assessed under normoxic and hypoxic conditions by measuring OCR, MMP, and ATP levels. Under normoxic conditions, M9a+sh*EPAS1* cells exhibited a 16% increase in maximal OCR compared to M9a+shCTR cells and a 30% increase compared to M7/8+shCTR cells. Under hypoxia, ATP-linked OCR in M9a+sh*EPAS1* cells increased by 80% compared to M9a+shCTR cells and by 150% compared to M7/8+shCTR cells (Fig. 2B, C), indicating enhanced OXPHOS efficiency. Additionally, M9a+sh*EPAS1* cells displayed a 44% and 101% increase in MMP and a 21% and 61% increase in ATP levels compared to M9a+shCTR and M7/8+shCTR cells, respectively (Fig. 2D, E). However, compared to M9a+shCTR and M7/8+shCTR cells, respectively, M9a+sh*EPAS1* cells exhibited a 20% and 70% increase in total ROS levels, and a 10% and 25% increase in mitochondrial ROS (mtROS) levels, likely due to enhanced OXPHOS (Fig. 2F, G). Furthermore, the mitochondria of M9a+sh*EPAS1* cells maintained more well-defined cristae under hypoxia compared to M9a+shCTR and M7/8+shCTR cells (Fig. 2H). These findings suggest that the Tibetan-specific M9a haplogroup, coupled with an adaptive nuclear gene background, optimizes mitochondrial function under hypoxic conditions.

To further investigate the impact of hypoxia on cellular function, we next evaluated cell proliferation and apoptosis levels. Under hypoxic conditions, M9a+sh*EPAS1* cells exhibited the highest viability, with live cell percentages increasing by 55% and 109% compared to M9a+shCTR and M7/8+shCTR cells, respectively (Fig. 2I). In contrast, Annexin V-FITC staining showed that the percentage of apoptotic cells in M9a+sh*EPAS1* cells decreased by 18% and 33% compared to M9a+shCTR and M7/8+shCTR cells, respectively (Fig. 2J). Western blot analysis confirmed reduced levels of apoptotic markers, including Cleaved Caspase-3 (C-Caspase-3) and BAX in M9a+sh*EPAS1* cells under hypoxia (Fig. 2K). Collectively, these results demonstrate that M9a+sh*EPAS1* cells exhibit superior hypoxia tolerance.

### M9a^+^sh*EPAS1* cells attenuate inflammation by enhancing mitophagy under hypoxia

**Fig. 3:**
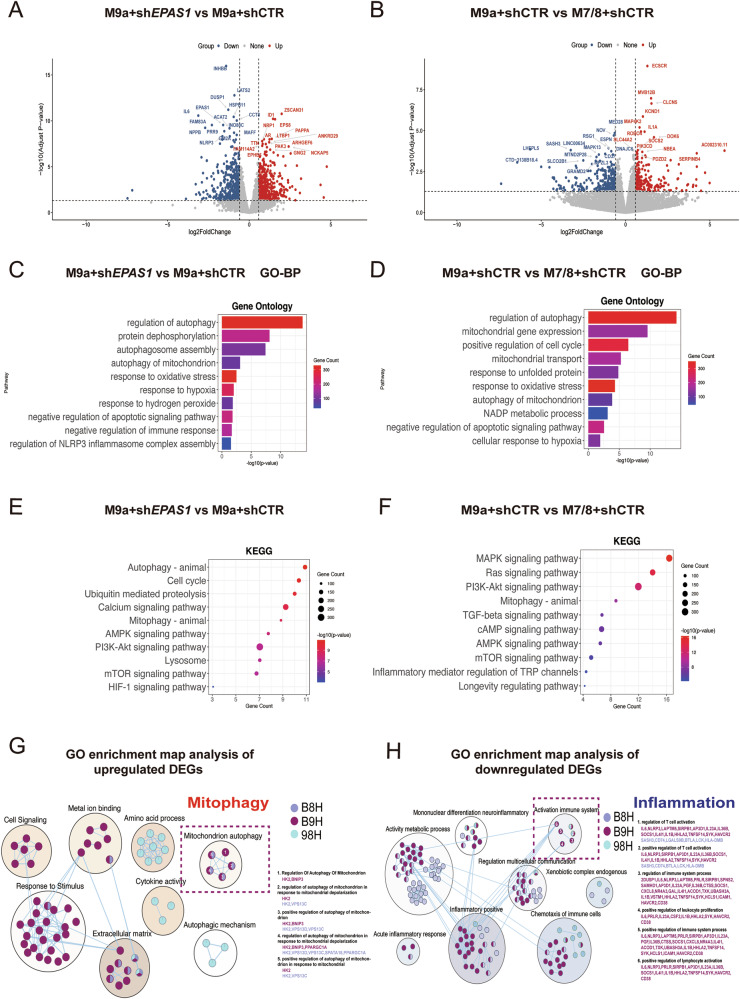
RNA-sequencing analysis of the M7/8+shCTR, M9a+shCTR and M9a+sh*EPAS1* cells under hypoxia. **A**, **B** Volcano plot analysis of M9a+sh*EPAS1* vs M9a+shCTR and M9a+shCTR vs M7/8+shCTR cells. **C**, **D** GO-BP analysis of DEGs from the M9a+sh*EPAS1* vs M9a+shCTR and M9a+shCTR vs M7/8+shCTR comparisons. **E**, **F** KEGG analysis of DEGs from the M9a+sh*EPAS1* vs M9a+shCTR and M9a+shCTR vs M7/8+shCTR comparisons. **G** GO enrichment map analysis of upregulated DEGs across the three groups. (M7/8+shCTR, M9a+shCTR, M9a+sh*EPAS1*). **H** GO enrichment map analysis of downregulated DEGs across the three groups. (M7/8+shCTR, M9a+shCTR, M9a+sh*EPAS1*). The experiment included three treatment groups, with four samples per group. B8H: M9a+sh*EPAS1* vs M7/8+shCTR cells; B9H: M9a+sh*EPAS1* vs M9a+shCTR cells; 98H: M9a+shCTR vs M7/8+shCTR cells.

To investigate the mechanism of Tibetan adaptation to hypoxia, RNA sequencing was performed on three cell lines (M7/8+shCTR, M9a+shCTR, M9a+sh*EPAS1*). Volcano plots revealed significant downregulation of Dual Specificity Phosphatase 1 (*DUSP1*) in M9a+sh*EPAS1* cells relative to both M9a+shCTR and M7/8+shCTR controls. This downregulation was associated with activation of AMPK and MAPK pathways (Fig. 3A, Supplementary Fig. S2A). Concomitant suppression of NLR family pyrin domain-containing 3 (*NLRP3*), interleukin 6 (*IL6*), and inhibin subunit Beta B (*INHBB*) specifically in M9a+sh*EPAS1* versus M9a+shCTR indicated attenuated inflammation, while transcriptomic profiles showed no divergence between M9a+shCTR and M7/8+shCTR (Fig. 3A, B). Gene Ontology (GO) analysis revealed that the biological processes (BP) of mitophagy, apoptosis, inflammation, and oxidative stress response were enriched in the M9a+sh*EPAS1* versus M9a+shCTR comparison (Fig. 3C), whereas DEGs from the M9a+shCTR versus M7/8+shCTR comparison showed enrichment of pathways related to mitophagy, mitochondrial gene expression, apoptosis, and the unfolded protein response (Fig. 3D). The Kyoto Encyclopedia of Genes and Genomes (KEGG) pathway analysis of M9a+sh*EPAS1* versus M9a+shCTR highlighted mitophagy, cell cycle, PI3K-AKT, and HIF-1α signaling (Fig. 3E), while the comparison of M9a+shCTR versus M7/8+shCTR revealed enrichment in MAPK, PI3K-AKT, mTOR, and TGF-β signaling pathways. (Fig. 3F). Critically, both GO and KEGG patterns for M9a+sh*EPAS1* versus M7/8+shCTR recapitulated those of M9a+sh*EPAS1* versus M9a+shCTR (Supplementary Fig. S2B, C). Subsequent GO enrichment map analysis of upregulated differentially expressed genes (DEGs) highlighted significant upregulation of mitophagy-related processes in M9a+sh*EPAS1* cells (Fig. 3G), and the downregulated DEGs revealed significant downregulation of inflammation-related processes in M9a+sh*EPAS1* cells (Fig. 3H). These results suggest that M9a+sh*EPAS1* cells may mitigate inflammation and cell apoptosis by enhancing mitophagy under hypoxia.

### M9a^+^sh*EPAS1* cells upregulate HIF-1α-BNIP3/NIX-mediated mitophagy and suppress mtDNA-mediated inflammation under hypoxia condition

**Fig. 4:**
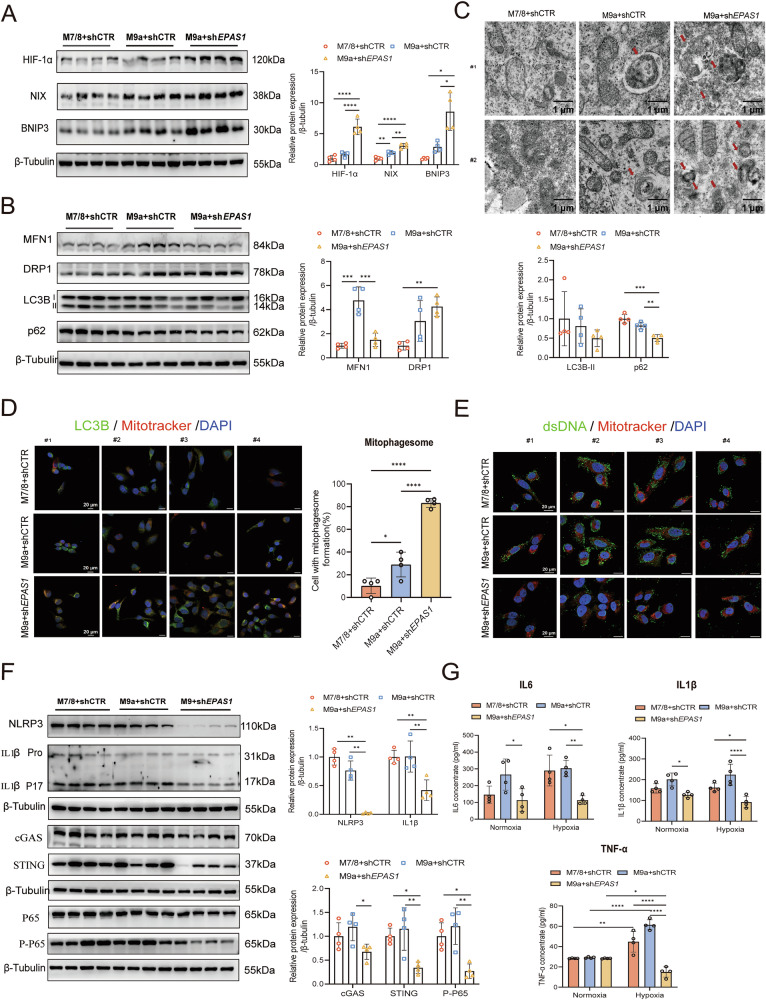
Enhanced mitophagy reduces mtDNA-mediated inflammation in M9a+sh*EPAS1* cells under hypoxia. **A**, **B** Western blot analysis of HIF-1α, BNIP3, NIX, MFN1, DRP1, LC3B, and p62 protein levels in M7/8+shCTR (*n* = 4), M9a+shCTR (*n* = 4), and M9a+sh*EPAS1* (*n* = 4) cells under hypoxia. Grayscale analysis of target protein levels was normalized to β-Tubulin. **C** Autophagosomes were analyzed in M7/8+shCTR (*n* = 2), M9a+shCTR (*n* = 2), and M9a+sh*EPAS1* (*n* = 2) cells using TEM under hypoxia. Scale bar: 1 μm. **D** Autophagic flux was assessed by quantifying the colocalization of LC3B (green) and Mitotracker (red) (marking mitophagosomes) using immunofluorescence in M7/8+shCTR (*n* = 4), M9a+shCTR (*n* = 4), and M9a+sh*EPAS1* (*n* = 4) cells under hypoxia. DAPI (blue) stains the nucleus. Scale bar: 20 μm. **E** Cytoplasmic free mtDNA was determined by staining the double-stranded DNA (dsDNA) (green) and Mitotracker (red) using immunofluorescence in M7/8+shCTR (*n* = 4), M9a+shCTR (*n* = 4), and M9a+sh*EPAS1* (*n* = 4) cells under hypoxia. DAPI (blue) stains the nucleus. dsDNA that does not colocalize with either the nucleus or mitochondria is considered cytoplasmic free mtDNA. Scale bar: 20 μm. **F** Western blot analysis of NLRP3, IL1β, cGAS, STING, P65, and P-P65 protein levels in M7/8+shCTR (*n* = 4), M9a+shCTR (*n* = 4), and M9a+sh*EPAS1* (*n* = 4) cells under hypoxia. Grayscale analysis of target protein levels was normalized to β-Tubulin. **G** Inflammatory cytokines IL6, IL1β, and TNF-α in the culture medium were measured in M7/8+shCTR (*n* = 4), M9a+shCTR (*n* = 4), and M9a+sh*EPAS1* (*n* = 4) cells under normoxic and hypoxic conditions using an ELISA kit. All cells were cultured under normoxia (21% O₂) or hypoxia (1% O₂) for 48 h.#1–4 represent individual samples. Data are presented as the mean ± SD of three independent experiments. One-way ANOVA followed by Tukey’s test; *P < 0.05; **P < 0.01; ***P < 0.001; ****P < 0.0001.

Hypoxia-induced mitophagy is typically mediated by BNIP3/NIX and regulated by HIF-1α [28], so we first excluded the involvement of PINK1/PARKIN-mediated mitophagy in Tibetan hypoxia adaptation (Supplementary Fig. S3A). Immunoblotting revealed that *EPAS1* knockdown resulted in more than a 3.5-fold increase in HIF-1α protein expression under hypoxia, leading to upregulation of downstream mitophagy-related proteins BNIP3 and NIX in M9a+sh*EPAS1* cells (Fig. 4A). Mitophagy, dependent on mitochondrial fission, was evidenced by decreased levels of mitochondrial fusion protein 1 (MFN1) and increased levels of dynamin-related protein 1 (DRP1). Furthermore, reduced levels of autophagy markers LC3B-II and p62 proteins indicated enhanced mitophagy in M9a+sh*EPAS1* cells under hypoxia (Fig. 4B). Transmission electron microscopy (TEM) revealed an increase in autophagosomes, while immunofluorescence analysis showed enhanced colocalization of LC3B with MitoTracker-labeled mitochondria (marking mitophagosomes) in M9a+sh*EPAS1* cells, further indicating enhanced mitophagy (Fig. 4C, D). M9a+shCTR cells also showed slightly higher mitophagy levels compared to M7/8+shCTR cells, as demonstrated by the expression of mitophagy-related proteins and the number of mitophagosomes and autophagosomes (Fig. 4A–D). These results suggest that HIF-1α-BNIP3/NIX-mediated mitophagy is enhanced in M9a+sh*EPAS1* cells.

Mitophagy is critical for cellular homeostasis, as insufficient mitophagy can lead to the release of mitochondrial components, such as mtDNA, into the cytoplasm, triggering inflammation [29]. We hypothesize that enhanced mitophagy would reduce inflammation in Tibetans. To test this, we first assessed cytoplasmic free mtDNA levels under hypoxia using immunofluorescence, and M9a+sh*EPAS1* cells exhibited significantly reduced cytoplasmic free mtDNA compared to both M9a+shCTR and M7/8+shCTR cells (Fig. 4E). We then evaluated inflammatory pathways associated with mtDNA, including the NLRP3 inflammasome and the cGAS-STING pathway. These analyses revealed a marked decrease in these inflammatory pathways in M9a+sh*EPAS1* cells under hypoxia (Fig. 4F). Additionally, the levels of proinflammatory cytokines IL6, IL1β, and TNF-α were significantly reduced in the culture medium of M9a+sh*EPAS1* cells (Fig. 4G). mRNA analysis further confirmed decreased expression of proinflammatory factors such as *NLRP3*, *IL6*, *IL1B*, *TNFA*, and *IL1A*, along with increased levels of the anti-inflammatory factor *IL10* in M9a+sh*EPAS1* cells (Supplementary Fig. S3B). However, these effects were not observed in M9a+shCTR cells compared to M7/8+shCTR cells (Fig. 4E–G, Supplementary Fig. S3B). These findings suggest that enhanced mitophagy reduces cytoplasmic free mtDNA levels, thereby mitigating inflammation in Tibetan-specific M9a+sh*EPAS1* cells.

### ROS drive HIF-1α-BNIP3/NIX-mediated mitophagy and alleviate cell apoptosis and inflammation in M9a+sh*EPAS1* cells during hypoxia

**Fig. 5:**
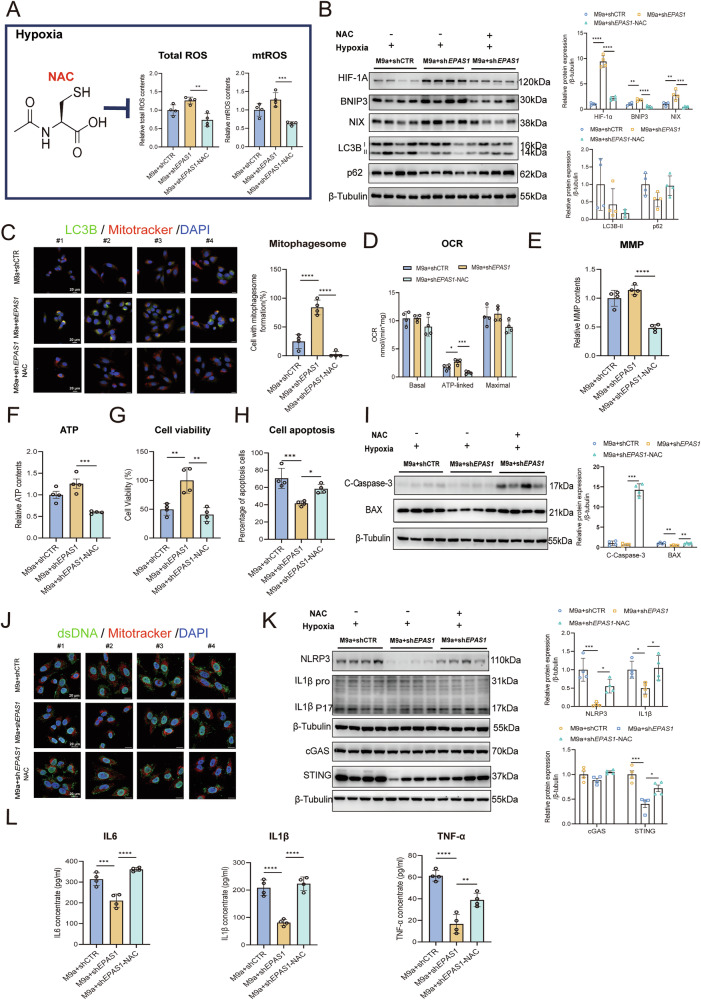
NAC inhibits HIF-1α stability and BNIP3/NIX-mediated mitophagy, exacerbating mtDNA-mediated inflammation in M9a+sh*EPAS1* cells during hypoxia. **A** Total ROS and mtROS contents were determined in the M9a+shCTR (*n* = 4), M9a+sh*EPAS1* (*n* = 4) and M9a+sh*EPAS1*-NAC (*n* = 4). The mtROS and total ROS contents in the M9a+sh*EPAS1* and M9a+sh*EPAS1*-NAC cells were normalized to those in the M9a+shCTR cells. **B** Western blot analysis of HIF-1α, BNIP3, NIX, LC3B, and p62 protein levels in the three cell lines under hypoxia. Grayscale analysis of target protein levels was normalized to β-Tubulin. **C** Autophagic flux was quantified by colocalizing LC3B (green) and Mitotracker (red) (mitophagosomes) using immunofluorescence in the three cell lines under hypoxia. DAPI (blue) stains the nucleus. Scale bar: 20 μm. **D**–**F** Mitochondrial OCR (basal, ATP-linked, maximal), MMP, and ATP content were measured in the three cell lines under hypoxia. MMP and ATP levels in M9a+sh*EPAS1* and M9a+sh*EPAS1*-NAC cells were normalized to those in M9a+shCTR cells. **G** CCK-8 assay showing cell viability in the three cell lines under hypoxia. **H** Cell apoptosis was determined using Annexin V-FITC staining in the three cell lines under hypoxia. **I** Western blot analysis of C-Caspase-3 and BAX protein levels in the three cell lines under hypoxia. Grayscale analysis of target protein levels was normalized to β-Tubulin. **J** Cytoplasmic free mtDNA was determined by staining dsDNA (green) and Mitotracker (red) using immunofluorescence in the three cell lines under hypoxia. DAPI (blue) stains the nucleus. dsDNA that does not colocalize with either the nucleus or mitochondria is considered cytoplasmic free mtDNA. Scale bar: 20 μm. **K** Western blot analysis of NLRP3, IL1β, cGAS, and STING protein levels in the three cell lines under hypoxia. Grayscale analysis of target protein levels was normalized to β-Tubulin. **L** Inflammatory cytokines IL6, IL1β, and TNF-α in the culture medium supernatant were measured in the three cell lines under hypoxia using an ELISA kit. The three cell lines, M9a+shCTR (*n* = 4), M9a+sh*EPAS1* (*n* = 4), and M9a+sh*EPAS1*-NAC (*n* = 4), were treated with hypoxia (1% O₂) for 48 h, with NAC treatment (10 mM) administered during the last 24 h.#1–4 represent individual samples. Data are presented as the mean ± SD of three independent experiments. One-way ANOVA followed by Dunnett’s test; *P < 0.05; **P < 0.01; ***P < 0.001; ****P < 0.0001.

To identify the cause of induced mitophagy, we explored the role of ROS, given their involvement in stabilizing HIF-1α [22, 28]. We hypothesized that elevated ROS levels might trigger mitophagy. Indeed, M9a+sh*EPAS1* cells exhibited increased levels of both total ROS and mtROS (Fig. 2F, G). To further investigate the role of ROS, we treated M9a+sh*EPAS1* cells with the broad-spectrum antioxidant N-acetylcysteine (NAC) under hypoxic conditions. NAC treatment significantly neutralized both total ROS and mtROS (Fig. 5A). Furthermore, NAC markedly disrupted HIF-1α stability, even under hypoxia. This disruption was accompanied by a reduction in the number of mitophagosomes and decreased levels of BNIP3, NIX, and LC3-II, while the p62 level increased, indicating attenuated mitophagy in M9a+sh*EPAS1* cells following NAC treatment (Fig. 5B, C).

We next assessed mitochondrial function by measuring OCR, MMP, and ATP levels. NAC treatment significantly reduced ATP-linked oxygen consumption in M9a+sh*EPAS1* cells (Fig. 5D), and both MMP and ATP levels were also diminished (Fig. 5E, F). To evaluate the effects on cell proliferation and apoptosis, we observed that NAC treatment decreased cell proliferation and increased apoptosis in M9a+sh*EPAS1* cells, as indicated by a reduction in live cell percentage, elevated levels of C-Caspase-3 and BAX, and a higher proportion of Annexin V-positive cells (Fig. 5G–I). In addition, we examined inflammation-related parameters. NAC treatment increased cytoplasmic free mtDNA levels and activated mtDNA-related inflammatory pathways, leading to elevated levels of inflammatory factors in M9a+sh*EPAS1* cells (Fig. 5J–L, Supplementary Fig. S4A).

In summary, these findings suggest that inhibiting ROS with NAC downregulates HIF-1α and BNIP3/NIX-mediated mitophagy, thereby exacerbating cell apoptosis and inflammation in M9a+sh*EPAS1* cells. These results underscore ROS as a key trigger for mitophagy by stabilizing HIF-1α.

### HIF-1α protects the M9a+sh*EPAS1* cells against hypoxia-induced apoptosis and inflammation by upregulating mitophagy during hypoxia

**Fig. 6:**
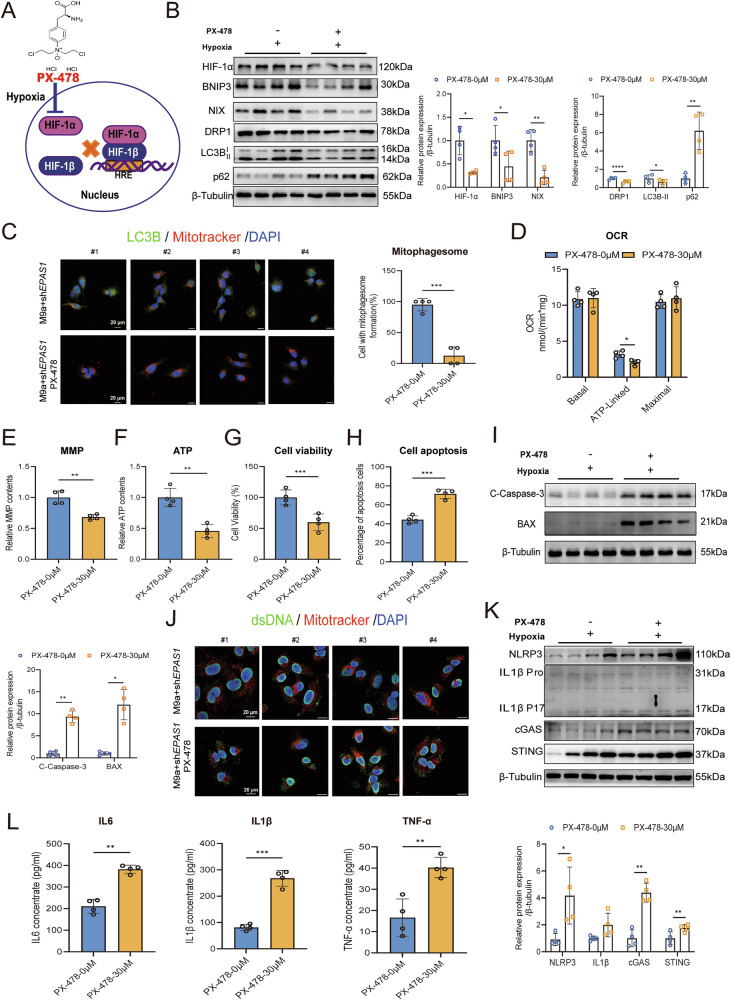
Downregulation of HIF-1α inhibits BNIP3/NIX-mediated mitophagy and exacerbates mtDNA-mediated inflammation in M9a+sh*EPAS1* cells during hypoxia. **A** Schematic diagram of the mechanism of action of PX-478. **B** Western blot analysis of HIF-1α, BNIP3, NIX, DRP1, LC3B, and p62 protein levels in M9a+sh*EPAS1* cells following PX-478 treatment under hypoxia. Grayscale analysis of protein levels was normalized to β-Tubulin. **C** Autophagic flux was quantified by colocalizing LC3B (green) and Mitotracker (red) (mitophagosomes) using immunofluorescence in M9a+sh*EPAS1* cells following PX-478 treatment under hypoxia. DAPI (blue) stains the nucleus. Scale bar: 20 μm. **D**–**F** Mitochondrial OCR (basal, ATP-linked, maximal), MMP, and ATP levels were determined in M9a+sh*EPAS1* cells after PX-478 treatment under hypoxia. **G** CCK-8 assay showing cell viability in M9a+sh*EPAS1* cells after PX-478 treatment under hypoxia. **H** Cell apoptosis was measured in M9a+sh*EPAS1* cells after PX-478 treatment under hypoxia using Annexin V-FITC staining. **I** Western blot analysis of C-Caspase-3 and BAX protein levels in M9a+sh*EPAS1* cells following PX-478 treatment under hypoxia. Grayscale analysis of protein levels was normalized to β-Tubulin. **J** Cytoplasmic free mtDNA was determined by staining dsDNA (green) and Mitotracker (red) in M9a+sh*EPAS1* cells following PX-478 treatment under hypoxia. DAPI (blue) stains the nucleus. dsDNA that does not colocalize with either the nucleus or mitochondria is considered cytoplasmic free mtDNA. Scale bar: 20 μm. **K** Western blot analysis of NLRP3, IL1β, cGAS, and STING protein levels in M9a+sh*EPAS1* cells after PX-478 treatment under hypoxia. Grayscale analysis of protein levels was normalized to β-Tubulin. **L** Inflammatory cytokines IL6, IL1β, and TNF-α in the culture medium were measured in M9a+sh*EPAS1* cells after PX-478 treatment under hypoxia using an ELISA kit. M9a+sh*EPAS1* cells (*n* = 4) were treated with 30 μM PX-478 (for the last 22 h) under hypoxia (1% O₂) for 48 h. #1–4 represent individual samples. Data are presented as the mean ± SD of three independent experiments. Two-tailed, paired Student’s *t* tests; *P < 0.05; **P < 0.01; ***P < 0.001.

The role of HIF-1α in regulating mitophagy in M9a+sh*EPAS1* cells was examined by treatment with PX-478, a HIF-1α inhibitor (Fig. 6A). PX-478 effectively suppressed HIF-1α and downstream NIX protein expression in a dose-dependent manner (Supplementary Fig. S5A). Inhibition of HIF-1α significantly decreased the expression of mitophagy-related proteins BNIP3, NIX, DRP1, and LC3B, while increasing the expression of p62 (Fig. 6B). Furthermore, the impairment of mitophagy following HIF-1α knockdown faithfully modeled the inhibitor treatment phenotype, an effect that was partially attenuated upon NIX overexpression (Supplementary Fig. S5B, C). Immunofluorescence analysis further revealed a reduction in mitophagosome formation following PX-478 treatment (Fig. 6C), indicating that BNIP3/NIX-mediated mitophagy is primarily regulated by HIF-1α.

Subsequent assessments of mitochondrial function demonstrated that PX-478 treatment severely impaired mitochondrial activity, as evidenced by decreased ATP-linked OCR, reduced MMP, and lower ATP levels (Fig. 6D–F). Additionally, PX-478 treatment led to reduced cell viability, elevated levels of apoptosis markers, and an increased proportion of Annexin V-positive cells, suggesting that HIF-1α inhibition significantly exacerbated hypoxia-induced apoptosis in M9a+sh*EPAS1* cells (Fig. 6G–I). Analysis of inflammation-related parameters showed that PX-478 significantly elevated mtDNA-mediated inflammatory responses, as indicated by increased levels of inflammation markers in M9a+sh*EPAS1* cells (Fig. 6J–L, Supplementary Fig. S5D).

Taken together, these findings clarify that HIF-1α regulates BNIP3/NIX-mediated mitophagy, mitigating apoptosis and inflammation in M9a+sh*EPAS1* cells. This underscores the role of HIF-1α as an upstream regulator of BNIP3/NIX-mediated mitophagy.

### Blocking mitophagy reduces mitochondrial function, aggravates mtDNA-mediated inflammation, and increases cell apoptosis in M9a+sh*EPAS1* cells during hypoxia

**Fig. 7:**
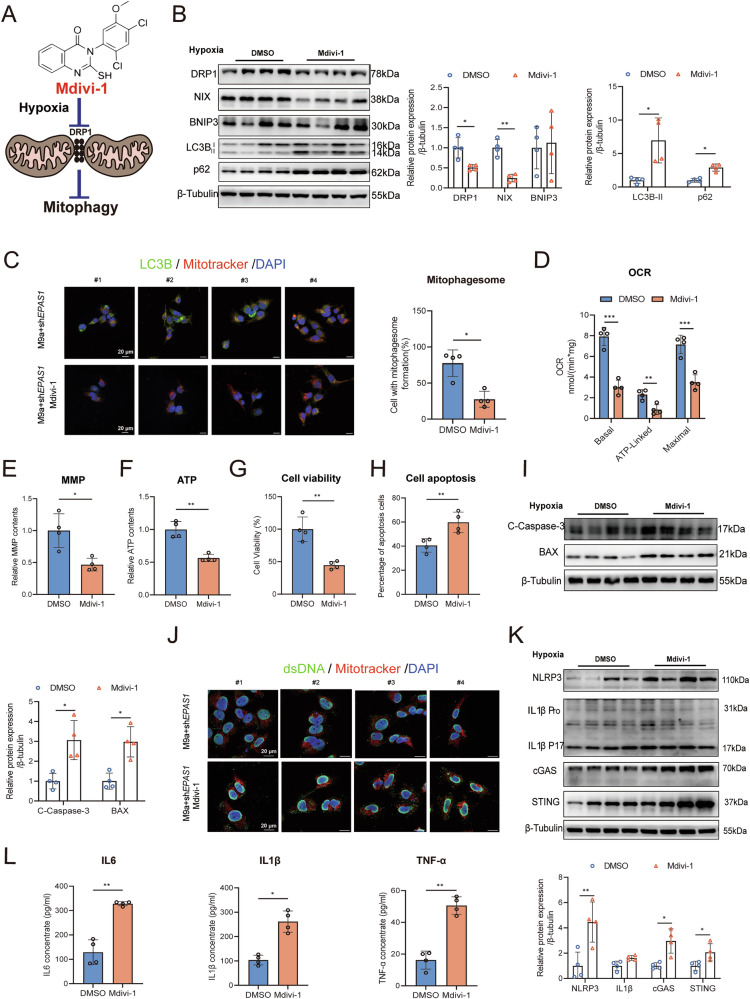
Mdivi-1 inhibited mitophagy and exacerbated mtDNA-mediated inflammation in M9a+sh*EPAS1* cells during hypoxia. **A** Schematic diagram of the mechanism of action of Mdivi-1. **B** Western blot analysis of DRP1, NIX, BNIP3, LC3B, and p62 protein levels in M9a+sh*EPAS1* cells after Mdivi-1 treatment under hypoxia. Grayscale analysis of protein levels was normalized to β-Tubulin. **C** Autophagic flux was assessed by quantifying the colocalization of LC3B (green) and Mitotracker (red) (mitophagosomes) using immunofluorescence in M9a+sh*EPAS1* cells after Mdivi-1 treatment under hypoxia. DAPI (blue) stains the nucleus. Scale bar: 20 μm. **D**–**F** Mitochondrial OCR (basal, ATP-linked, maximal), MMP, and ATP content were measured in M9a+sh*EPAS1* cells after Mdivi-1 treatment under hypoxia. **G** CCK-8 assay showing cell viability in M9a+sh*EPAS1* cells after Mdivi-1 treatment under hypoxia. **H** Cell apoptosis was determined in M9a+sh*EPAS1* cells after Mdivi-1 treatment under hypoxia using Annexin V-FITC staining. **I** Western blot analysis of C-Caspase-3 and BAX protein levels in M9a+sh*EPAS1* cells after Mdivi-1 treatment under hypoxia. Grayscale analysis of protein levels was normalized to β-Tubulin. **J** Cytoplasmic free mtDNA was determined by staining dsDNA (green) and Mitotracker (red) using immunofluorescence in M9a+sh*EPAS1* cells after Mdivi-1 treatment under hypoxia. DAPI (blue) stains the nucleus. dsDNA that does not colocalize with either the nucleus or mitochondria is considered cytoplasmic free mtDNA. Scale bar: 20 μm. **K** Western blot analysis of NLRP3, IL1β, cGAS, and STING protein levels in M9a+sh*EPAS1* cells after Mdivi-1 treatment under hypoxia. Grayscale analysis of protein levels was normalized to β-Tubulin. **L** Inflammatory cytokines IL6, IL1β, and TNF-α in the culture medium were measured using ELISA kits in M9a+sh*EPAS1* cells after Mdivi-1 treatment under hypoxia. M9a+sh*EPAS1* cells (*n* = 4) were treated with 50 μM Mdivi-1 or DMSO (pre-treatment for 4 h, followed by treatment for 48 h) under hypoxia (1% O₂).#1–4 represent individual samples. Data are presented as the mean ± SD of three independent experiments. Two-tailed, paired Student’s *t* tests; *P* < 0.05; *P < 0.05; **P < 0.01; ***P < 0.001.

To further elucidate the role of mitophagy in the adaptation of Tibetans to hypoxia, we used Mdivi-1, a selective DRP1 inhibitor, to suppress mitophagy (Fig. 7A). Mdivi-1 effectively inhibited the expression of DRP1 and the mitophagy-related protein NIX in a dose-dependent manner (Supplementary Fig. S6A). Western blot analysis and immunofluorescence confirmed a significant reduction in mitophagy in M9a+sh*EPAS1* cells treated with Mdivi-1 (Fig. 7B, C). Additionally, Mdivi-1 significantly decreased the OCR, including basal, ATP-linked, and maximal rates in M9a+sh*EPAS1* cells (Fig. 7D). Both MMP and ATP levels were also reduced following Mdivi-1 treatment (Fig. 7E, F). Furthermore, cell viability was significantly decreased (Fig. 7G), while apoptosis levels increased, as indicated by elevated markers such as C-Caspase-3 and BAX, and a higher proportion of Annexin V-positive cells (Fig. 7H, I). Importantly, Mdivi-1 treatment led to a marked increase in mtDNA-mediated inflammation-related indicators (Fig. 7J–L, Supplementary Fig. S6B).

These results underscore the critical role of mitophagy in mitigating apoptosis and inflammation in M9a+sh*EPAS1* cells under hypoxia, highlighting its importance in adapting to the high-altitude hypoxic environment encountered by Tibetans.

## Discussion

This study provides evidence that the Tibetan-specific M9a haplogroup, in conjunction with the adaptive downregulation of the nuclear gene *EPAS1*, exhibits enhanced mitochondrial function, reduced inflammation, and decreased cell apoptosis under hypoxic conditions. These effects were mediated through the activation of HIF-1α-BNIP3/NIX-driven mitophagy, with ROS playing a critical role in initiating this process in Tibetan adaptation to hypoxia. This was supported by experiments using the antioxidant NAC, the HIF-1α inhibitor PX-478, and the mitophagy inhibitor Mdivi-1, which demonstrated that ROS trigger HIF-1α-BNIP3/NIX-mediated mitophagy. This mitophagy was essential for promoting high-energy metabolism and reducing inflammation, suggesting that HIF-1α may represent a promising regulatory target for facilitating adaptation to hypoxic environments.

The mitochondrial cybrid technique in 143B cells is widely used to study various diseases, exploring how mtDNA variants influence mitochondrial functions in different haplogroups, such as neurological disorders, cardiovascular diseases, cerebrovascular diseases, aging, and cancer [30, 31]. Recent evidence has highlighted the association between mitochondrial haplogroups and high-altitude hypoxia adaptation, with the M9a haplogroup receiving particular attention. This haplogroup is the most prevalent among Tibetans and is strongly correlated with altitude [13–15]. In our Lhasa samples, the M9a haplogroup accounted for ~28.5%, consistent with previous reports [16, 17]. Using transmitochondrial cell technology, we generated Tibetan-specific M9a cybrids and plain-specific M7/8 cybrids as controls. The M9a haplogroup exhibited superior mitochondrial function compared to the M7/8 haplogroups under hypoxic conditions, demonstrated by enhanced OXPHOS efficiency in ATP production and elevated MMP levels. Additionally, the conserved T3394C mutation in the M9a haplogroup, located within mitochondrial complex I and involving the substitution of an evolutionarily conserved tyrosine (Y) with histidine (H) at position 30 (Y30H), has been shown to influence complex I activity under normoxic conditions and is enriched in the Tibetan population [17]. In this study, we observed significantly higher expression levels of mitochondrial complex I and supercomplex I in the M9a haplogroup compared to the M7/8 haplogroup under hypoxic conditions, leading to enhanced mitochondrial function and increased oxygen utilization, as evidenced by elevated OCR, MMP, and ATP production.

Hypoxia-inducible factors HIF-1α and HIF-2α play pivotal roles in orchestrating metabolic adaptation to hypoxia by modulating downstream gene expression pathways [32]. Although these factors are both involved in the hypoxic response, they regulate overlapping but distinct sets of genes and cellular processes. For instance, both HIF-1α and HIF-2α can activate *VEGFA*, a gene implicated in the pathogenesis of pulmonary hypertension. HIF-1α predominantly regulates glycolysis, OXPHOS, and mitochondrial biogenesis, while HIF-2α primarily regulates erythropoiesis, blood vessel remodeling, and cell cycle progression [33–35]. Notably, *EPAS1* (which encodes HIF-2α) has shown a strong selection signal and is correlated with lower hemoglobin concentrations in Tibetans [2–5], whereas *HIF1A* (which encodes HIF-1α) has not shown clear signs of selection among Tibetans [3]. Our findings demonstrate that downregulation of HIF-2α leads to a significant upregulation of HIF-1α and the downstream mitophagy pathway, which plays a protective role under hypoxia. This may help explain the lack of natural selection for *HIF1A* in the Tibetan population. Furthermore, our study revealed that Tibetan M9a+sh*EPAS1* cells exhibited the most robust mitochondrial function, supporting the hypothesis of increased oxygen transport and utilization capacity in Tibetans [36, 37].

Historically, ROS were considered disruptors of physiological functions and were implicated in various pathological processes, including diabetes, neurodegenerative diseases, aging, and more [38, 39]. However, recent evidence highlights the critical role of ROS in maintaining cellular homeostasis [40]. Mitochondria are considered one of the primary sources of ROS, and hypoxia exacerbates their production by inhibiting the mitochondrial respiratory chain [41]. Research suggests that ROS directly stabilize HIF-1α under hypoxic conditions by oxidizing Fe^2+^ to Fe^3+^ at the active site of prolyl hydroxylase enzymes [42–44]. Additionally, cytosolic hydrogen peroxide (H_2_O_2_) acts as the primary form of ROS responsible for stabilizing HIF-1α. This is supported by findings that the expression of glutathione peroxidase or catalase, rather than superoxide dismutase, prevents HIF-1α stabilization under hypoxia [42]. Our study corroborates this link between ROS and HIF-1α stabilization, showing that ROS play a protective role under hypoxia by stabilizing HIF-1α. Moreover, we found that the antioxidant NAC, a glutathione precursor, effectively counteracted ROS-dependent HIF-1α stabilization, even under hypoxic conditions. This resulted in maladaptive phenotypes, including worsened mitochondrial dysfunction, cell apoptosis, and inflammation, emphasizing the importance of ROS in maintaining cellular homeostasis during hypoxia. Therefore, it is crucial to recognize the physiological significance of moderate ROS levels. The use of antioxidants, particularly catalase analogs, should be approached cautiously, as they may disrupt ROS-mediated regulatory mechanisms, especially in hypoxia-related diseases. Future research should aim to identify the threshold at which ROS production shifts from being physiologically beneficial to potentially harmful. Additionally, it is essential to characterize the specific types of ROS involved in various physiological or pathological contexts to allow for precise regulation that maintains their beneficial effects while mitigating their harmful impacts.

Receptor-mediated mitophagy involves mitochondrial outer membrane proteins such as BNIP3, NIX, and FUNDC1, which directly interact with LC3B to facilitate the clearance of damaged mitochondria. Studies have shown that BNIP3/NIX-mediated mitophagy predominates under hypoxic conditions and is regulated by HIF-1α [28]. Our investigation reveals the protective role of BNIP3/NIX-mediated mitophagy, rather than PINK1/PARKIN-mediated mitophagy, in Tibetan hypoxia adaptation. Additionally, we demonstrated that HIF-1α acts upstream of receptor-mediated mitophagy, based on our findings that loss of HIF-1α function—achieved through either pharmacological inhibition (PX-478) or genetic downregulation—markedly suppressed BNIP3/NIX-mediated mitophagy. Consistent with our findings, previous studies have highlighted the cytoprotective role of HIF-1α in ischemia-reperfusion injury and acute kidney injury through the activation of receptor-mediated mitophagy pathways [45, 46]. Furthermore, the degradation of damaged mitochondria via enhanced mitophagy facilitates organelle renewal, mitigates mitochondrial aging, and preserves OXPHOS efficiency [47]. This aligns with our research, underscoring the enhancement of mitochondrial function through mitophagy under hypoxic conditions.

Mitophagy deficiency can lead to the release of mitochondrial components, such as mtDNA, into the cytoplasm, triggering inflammatory responses [48]. Cytosolic mtDNA has been implicated in activating the cGAS-STING inflammation pathway, which contributes to inflammation in various diseases, including aging, systemic lupus erythematosus, and neurodegenerative conditions [29, 49, 50]. Additionally, mtDNA is associated with inflammation mediated by the NLRP3 inflammasome pathway [51]. There is a bidirectional regulatory relationship between mitophagy and inflammation: insufficient mitophagy can initiate inflammation, while reducing inflammation can promote mitophagy [45]. Previous research suggests that hypoxia exacerbates inflammatory responses [52]. However, Tibetan populations display lower levels of inflammation compared to immigrant Han populations [10]. Moreover, Tibetans show lower susceptibility to acute mountain sickness (AMS) and high-altitude pulmonary edema (HAPE), conditions in which inflammation is believed to play a significant role [53, 54]. The hypoxia-induced inflammatory response is coordinated by transcription factors HIFs and NF-κB [55]. In line with prior findings, our study demonstrated that enhanced mitophagy mitigates mtDNA-mediated inflammation under hypoxia. This was evidenced by the downregulation of cytoplasmic mtDNA-related cGAS-STING and NLRP3 inflammasome signaling pathways, as well as reduced expression of proinflammatory factors IL6, IL1β, and TNF-α in Tibetan M9a+sh*EPAS1* cells. These results were corroborated by experiments using the mitophagy inhibitor Mdivi-1. Besides, our findings showed that the Tibetan-specific M9a haplogroup enhanced mitophagy but did not significantly reduce inflammation when compared with the M7/8 haplogroup. This may be attributed to an insufficient degree of mitophagy enhancement to effectively clear damaged mitochondria. In contrast, the combination of the M9a haplogroup with sh*EPAS1* significantly augmented BNIP3/NIX-mediated mitophagy and markedly reduced mtDNA-mediated inflammation under hypoxia. This suggests that the downregulation of *EPAS1* not only correlates with lower hemoglobin concentrations in Tibetans but also contributes to reduced inflammation and enhanced energy metabolism through improved HIF-1α-BNIP3/NIX-mediated mitophagy, highlighting the effectiveness of natural selection. Consistent with our findings, a recent study identified that the Tibetan adaptive haplotype PHD2 (D4E/C127S) alleviates the inflammatory response in monocytes by downregulating HIF-2α [11]. Our study reveals the critical role of HIF-1α-mediated mitophagy in alleviating hypoxia-induced inflammation. Notably, emerging evidence reveals that HIF-1α transcriptionally activates heat shock factor 1 (HSF1), the master regulator of the heat shock response [56, 57]. Furthermore, research finds HSF1 not only induces heat shock protein expression to maintain proteostasis but also directly interacts with the NLRP3 inflammasome under stress conditions, thereby suppressing its aberrant activation [58, 59]. HIF-1α likely orchestrates cytoprotection by coordinating these parallel anti-inflammatory pathways.

Our results indicate that ROS-HIF-1α-BNIP3/NIX-mediated mitophagy is a key mechanism underlying the lower inflammation and improved oxygen utilization observed in Tibetans. However, it is important to acknowledge the limitations inherent in mitochondrial haplogroup research techniques, which preclude the direct extrapolation of our findings to murine models. Future investigations using murine models are warranted to validate and further elucidate our results, thereby advancing our understanding of the mechanisms underlying hypoxia adaptation.

## Conclusion

This study uncovers a novel hypoxia adaptation mechanism in Tibetans mediated by mitochondria-nuclear crosstalk, wherein the enhancement of BNIP3/NIX-mediated mitophagy contributes to the reduction of hypoxia-induced inflammation and improves oxygen utilization. Furthermore, ROS function as signaling molecules to stabilize HIF-1α and regulate BNIP3/NIX-mediated mitophagy. Targeting the HIF-1α-BNIP3/NIX-mediated mitophagy pathway may offer a promising therapeutic strategy for the treatment of high-altitude illness.

## Methods and materials

### Cell line generation and genotyping

Whole blood samples from plain regions were obtained from the Taizhou Institute of Health Sciences, Fudan University, while samples from high-altitude regions were collected from healthy Tibetan individuals in Lhasa, Xizang. This study was approved by the Human Ethics Committee of Fudan University (Shanghai, China), informed consent was obtained from all participants. The method for constructing the transmitochondrial cell model was performed as previously described [60]. Briefly, the whole blood samples were divided into two parts: one part underwent DNA extraction using a whole blood DNA extraction kit (QIAGEN, Germany) for haplotype identification, while the other part was used for platelet isolation. Platelets were then fused with 143B ρ0 cells (mtDNA-depleted) using polyethylene glycol [60]. After a three-day culture, the cells were selected in Dulbecco’s Modified Eagle Medium (DMEM) supplemented with 10% dialyzed FBS (Thermo Fisher Scientific, Waltham, MA) without pyruvate or uracil. Surviving clones from this selection were identified as the desired cybrids. Whole mitochondrial genome sequencing was conducted using 24 pairs of mtDNA primers, as previously reported, via the Sanger method [61]. The identified single-nucleotide polymorphisms were compared with the global human phylogenetic tree SNPs available at http://www.phylotree.org/tree/index.htm to determine specific mtDNA haplogroups. In this study, 11 cybrids distributed in an mtDNA tree were constructed, including M9a cybrids (*n* = 5) and M7/8 cybrids (*n* = 6). The complete mitochondrial genomes of all cybrid cells are provided in Supplementary Tables S1–S11.

### Cell culture and treatments

The 143B ρ0 cells (lacking mtDNA) were generously provided by Professor Hezhi Fang from Wenzhou Medical University and were authenticated by STR profiling and tested for mycoplasma. Cells were cultured in DMEM (Thermo Fisher Scientific, Waltham, MA) supplemented with 10% fetal bovine serum (Thermo Fisher Scientific, Waltham), 100 μg/mL pyruvate, 50 μg/mL uridine, and 100 U/mL penicillin-streptomycin at 37 °C with 5% CO₂.

The remaining cell lines were cultured in DMEM containing 10% fetal bovine serum, 100 μg/mL pyruvate, and penicillin-streptomycin-amphotericin B solution. Cells were maintained in a normoxic incubator (37 °C, 5% CO₂, 21% O₂) or in a hypoxic workstation (Invivo2 400, Ruskinn, UK; 37 °C, 5% CO₂, 1% O₂). Typically, cells were first cultured under normoxic conditions for 24 h to allow adherence, and then transferred to a hypoxic workstation. To preclude reoxygenation, all cells designated for Western blot analysis or RNA extraction were subjected to three sequential washes with PBS within the hypoxic workstation, followed immediately by lysis using the appropriate buffer (RIPA lysis or Trizol reagent). Inhibitors were used as follows: N-acetylcysteine (NAC) was dissolved in the medium to a final concentration of 10 mM (diluted in ddH₂O, Sigma, USA), the HIF-1α inhibitor PX-478 was dissolved in ddH₂O to a final concentration of 30 μM (MCE, USA), and Mdivi-1 was used at 50 μM (diluted in DMSO, MCE, USA).

### Generation of sh*EPAS1* cell lines

Lentiviral vector plasmids, packaging plasmids, and transfection reagent Lipofectamine 3000 (Invitrogen, USA) were mixed and added to 293T cells to generate lentiviral particles. The cybrid cell lines were transfected with lentivirus, and stable cell lines were selected using 1 μg/mL puromycin (Sigma, USA). The NIX overexpression plasmid for transient transfection was purchased from YiXueSheng Biosciences Inc (Shanghai, China). Cells were transfected with the plasmid using Lipofectamine 3000 (Invitrogen, USA). The sequences for the short hairpin RNAs (shRNAs) targeting *EPAS1* and *HIF-1A* genes and the negative control were as follows:

ShCTR : 5′- TTCTCCGAACGTGTCACGT-3′ (CDS region);

sh*EPAS1* : 5′- caGTACCCAGACGGATTTCAA-3′ (CDS region);

sh*HIF-1A*: 5′- TGCTCTTTGTGGTTGGATCTA-3′ (CDS region).

### Total RNA extract and quantitative real-time PCR (qPCR) analysis

Total RNA was extracted using Trizol reagent (Thermo Fisher Scientific, USA). A reverse transcription kit (Vazyme, China) was employed to synthesize cDNA from the extracted RNA. For quantitative real-time PCR (qPCR), the qPCR mixture, comprising cDNA, SYBR Green master mix (Takara Bio, Dalian, China), and primers, was prepared and amplified using a QuantStudio™ 7 Flex Real-Time PCR System (Life Technologies, USA). The housekeeping gene β-Tubulin was used as an internal reference for normalization. The expression levels of target genes were calculated using the 2^−ΔΔCT^ method. Primer sequences used in this study are provided in Supplementary Table S12.

### RNA sequencing (RNA-Seq) analysis

RNA-Seq was employed to analyze the global gene expression patterns in three cell lines: M7/8+shCTR (*n* = 4), M9a+shCTR (*n* = 4), and M9a+sh*EPAS1* (*n* = 4). Gene expression quantification was performed using the Kallisto software based on the GRCh38 genome. A total of 35,630 transcriptionally active regions were evaluated. DEGs were identified using *p* < 0.05 and |logFC|> 1. Gene enrichment studies were conducted using KEGG and GO, and pathway enrichment clustering was performed using EnrichmentMap in Cytoscape (*Q* value < 0.05) and AutoAnnotate (Max annotations < 50). The analysis was conducted using R v4.3 and Linux Ubuntu.

### Immunoblotting and antibodies

Proteins were extracted using RIPA lysis buffer (CST, USA) supplemented with the protease inhibitor PMSF (Sigma, USA). Proteins were separated by 12% SDS-PAGE and transferred onto 0.22 μM PVDF membranes (Sigma, USA). Membranes were blocked with 5% non-fat milk for 2 h and incubated with primary antibodies at 4 °C overnight. The membranes were then incubated with HRP-conjugated secondary antibodies (CST, USA) for 1–2 h at room temperature. Primary antibodies were diluted 1:1000 in Primary Antibody Dilution Buffer (Beyotime, China), as listed in Supplementary Table S13. Western blots were visualized using ECL substrate (Yeasen, China), and protein bands were quantified using Gel-Pro-Analysis software.

### Blue native PAGE (BN-PAGE) and antibodies

To detect mitochondrial complexes, mitochondrial membrane proteins were extracted using n-dodecyl-D-maltoside (DDM) (Sigma, MO, USA) supplemented with PMSF (Sigma, MO, USA). Subsequently, 60 µg of proteins were separated by Blue Native Polyacrylamide Gel Electrophoresis (BN-PAGE) using a 4–16% gel.

For mitochondrial supercomplex detection, membrane proteins were extracted using digitonin (Sigma, MO, USA), supplemented with PMSF (Sigma, MO, USA). Sixty µg of proteins were separated by BN-PAGE using a 3–11% gel. After gel electrophoresis, the proteins were transferred to a membrane and blocked with 5% milk. Membranes were incubated with primary antibodies at 4 °C overnight, followed by incubation with alkaline phosphatase (AP)-conjugated secondary antibodies (CST, USA) for 4 h at room temperature. Visualization of protein bands was achieved using BCIP (6.67 mg/ml) and NBT (8 mg/ml). All primary antibodies were diluted 1:1000 in Primary Antibody Dilution Buffer (Beyotime, China), as listed in Supplementary Table S13. Protein bands were quantified using Gel-Pro-Analysis software.

### Oxygen consumption

Oxygen consumption in intact cells was evaluated using an Oxygraph-2k instrument (Oroboros, Austria), following established protocols. Basal respiration was measured first, followed by the addition of oligomycin (2.5 mM) (Sigma, MO, USA) to assess phosphorylation-coupled respiration. Finally, the uncoupling agent FCCP (0.1 mM) (Sigma, MO, USA) was introduced to determine maximal respiration. Oxygen consumption values were normalized to protein concentration.

### ATP measurements

ATP levels were quantified using an ATP measurement kit (Thermo Fisher Scientific, Waltham, MA, USA) according to the manufacturer’s instructions. Briefly, cells were washed with PBS and lysed with 100 µL ATP extraction solution (100 mM Tris, 4 mM EDTA, pH 7.75) by boiling at 100 °C for 90 s. Supernatants were collected by centrifugation at 10,000 × *g* for 1 min. The ATP content was determined by measuring the luminescence of the supernatants mixed with Luciferase Assay buffer using a Spark Multimode Reader (Tecan, Männedorf, Switzerland). ATP luminescence values were normalized to protein concentration.

### MMP measurements

MMP was assessed using the cationic fluorescent dye tetramethylrhodamine methyl ester (TMRM) (Thermo Fisher Scientific, Waltham, MA, USA). Cells were treated with a final concentration of 30 nM TMRM at 37 °C for 20 min. After incubation, cells were washed with PBS, and the fluorescence signal was measured using a Spark Multimode Reader (Tecan, Männedorf, Switzerland). MMP fluorescence values were normalized to protein concentration.

### Total ROS and mtROS measurements

Total ROS and mtROS were measured using the fluorescent probes H2DCFDA (Thermo Fisher Scientific, Waltham, MA, USA) and MitoSOX (Thermo Fisher Scientific, Waltham, MA, USA), respectively. Briefly, cells were washed with Hank’s Buffered Salt Solution (HBSS) (Beyotime, China), then resuspended in DMEM containing 10 µM H2DCFDA and 5 µM MitoSOX. The cells were incubated at 37 °C for 20 min. After incubation, cells were washed three times with HBSS, and the fluorescence signal was measured using a Spark Multimode Reader (Tecan, Männedorf, Switzerland). Total ROS and mtROS fluorescence values were normalized to protein concentration.

### Cell proliferation and apoptosis detection

Cell proliferation was assessed using the Cell Counting Kit-8 (CCK-8) (Beyotime, China). In brief, 6000 cells/well were seeded into a 96-well plate (5 wells per condition). After the respective treatments, 10 μL of CCK-8 solution was added to each well and incubated for 2 h at 37 °C. Absorbance at 450 nm was measured using a Spark Multimode Reader (Tecan, Männedorf, Switzerland).

Cell apoptosis was evaluated using the Annexin V-FITC Apoptosis Detection Kit (Beyotime, China). Cell precipitates were collected, washed with PBS, and stained with Annexin V-FITC dye in the dark at room temperature for 20 min, followed by incubation on ice. Samples were analyzed immediately using flow cytometry (Calibur, BD Biosciences, USA) and data were processed using FlowJo 10 software (BD Biosciences, USA).

### Immunofluorescence (IF) staining

Mitochondria were labeled with MitoTracker Red (200 nM) (Thermo Fisher Scientific, USA) at 37 °C for 30 min. Cells were then fixed with 4% paraformaldehyde (PFA) for 10 min at room temperature, followed by permeabilization with 0.25% Triton X-100 (Servicebio, China) in PBS for 10 min. After blocking with 2% bovine serum albumin (BSA) in PBS for 1 h at room temperature, cells were incubated overnight at 4 °C with primary antibodies (diluted 1:100 in 1% BSA). Cells were washed three times with PBS to remove unbound antibodies and then incubated with 488 fluorophore-conjugated secondary antibodies (diluted 1:400 in 1% BSA) for 1 h at room temperature in the dark. Nuclei were counterstained with 4’,6-diamidino-2-phenylindole (DAPI) (Thermo Fisher Scientific, USA) for 1 min at room temperature. Cells were mounted onto glass slides using Antifade Mounting Medium (Beyotime, China) to preserve fluorescence. Images were acquired using a Zeiss LSM880 (Zeiss, Germany) microscope with Airyscan.

### Transmission electron microscopy (TEM)

Briefly, cells were collected and fixed with 2.5% glutaraldehyde (Biossci, China). Biossci (Hubei) Biotechnology Co., Ltd was responsible for embedding and sectioning the samples. Sections with a thickness of 70 nm were observed using a Hitachi HT-7800 transmission electron microscope (Hitachi, HT-7800).

### Cytokine detection

A total of 200,000 cells were seeded in six-well plates and cultured under normoxic or hypoxic conditions. The cell culture supernatant was collected for the detection of inflammatory cytokines. Human IL-6, IL-1β, and TNF-α levels were measured using ELISA kits (LiankeBio, China) according to the manufacturer’s instructions. Briefly, samples were incubated in a 96-well polystyrene microplate pre-coated with monoclonal antibodies specific to each cytokine. The samples were allowed to bind to the solid phase and detection antibodies. After washing to remove unbound material, horseradish peroxidase-labeled streptavidin (Streptavidin-HRP) was added. Following another wash to remove excess Streptavidin-HRP, a color development substrate (TMB) was added. The plates were protected from light to allow color development. Absorbance was measured at a wavelength of 450 nm with a reference wavelength of 570 nm using a Spark Multimode Reader (Tecan, Männedorf, Switzerland).

### Statistical analysis

Data are presented as mean ± SD and were analyzed with GraphPad Prism 9.0 (GraphPad Software, San Diego, CA, USA). All analyses accounted for homogeneity of variances. Specifically, two-group comparisons used unpaired/paired two-tailed *t*-tests (with Welch’s correction if variances differed), while multi-group comparisons used one-way or two-way ANOVA (using Welch’s test for one-way when variances were unequal). Statistical significance was defined as *p* < 0.05.

## Supplementary information

Supplementary date

Full length uncropped original western blots.

## Data Availability

The data used to support the findings of this study are available from the corresponding author upon reasonable request. Full and uncropped western blots are included in a single supplemental file (named “Full length uncropped original western blots”). The raw RNA-seq data generated in this study can be found in the NCBI SRA online repository under the accession number PRJNA1354993.
