# Supplementary material for: Mitochondrial retrograde signaling initiates HIF-1α/BNIP3/NIX-mediated mitophagy in Tibetan high-altitude adaptation
Source: Cell Death Discov. 2026 Jan 6;12:81. doi: 10.1038/s41420-025-02933-8 (PMC12877009; doi:10.1038/s41420-025-02933-8)
Supplement: Supplementary file 1 — Supplementary date [file 41420_2025_2933_MOESM1_ESM.docx]

**Supplementary Information for:**

**Mitochondrial Retrograde Signaling Initiates HIF-1α/BNIP3/NIX-Mediated Mitophagy in Tibetan High-Altitude Adaptation**

**Yang Wei^1#^, Dayan Sun^1,2#^, Fei Wu^1#^, Shixuan Zhang^1^, Bowen Cai^1^, Yanyun Ma^3^, Hongxiang Zheng^1^, Xiangguang Shi^1,4^, Yi Li^1^, Shiguan Le^1^, Xiang Zhou^1^, Li Jin^1,5*^, Jiucun Wang^1,4,5*^**

*** Correspondence:**

Jiucun Wang, Email: jcwang@fudan.edu.cn

Li Jin, Email: [lijin@fudan.edu.cn](mailto:lijin@fudan.edu.cn)

**The PDF file includes:**

Supplementary Figures 1 to 6

Supplementary Table 1-13

**Supplementary Figures**


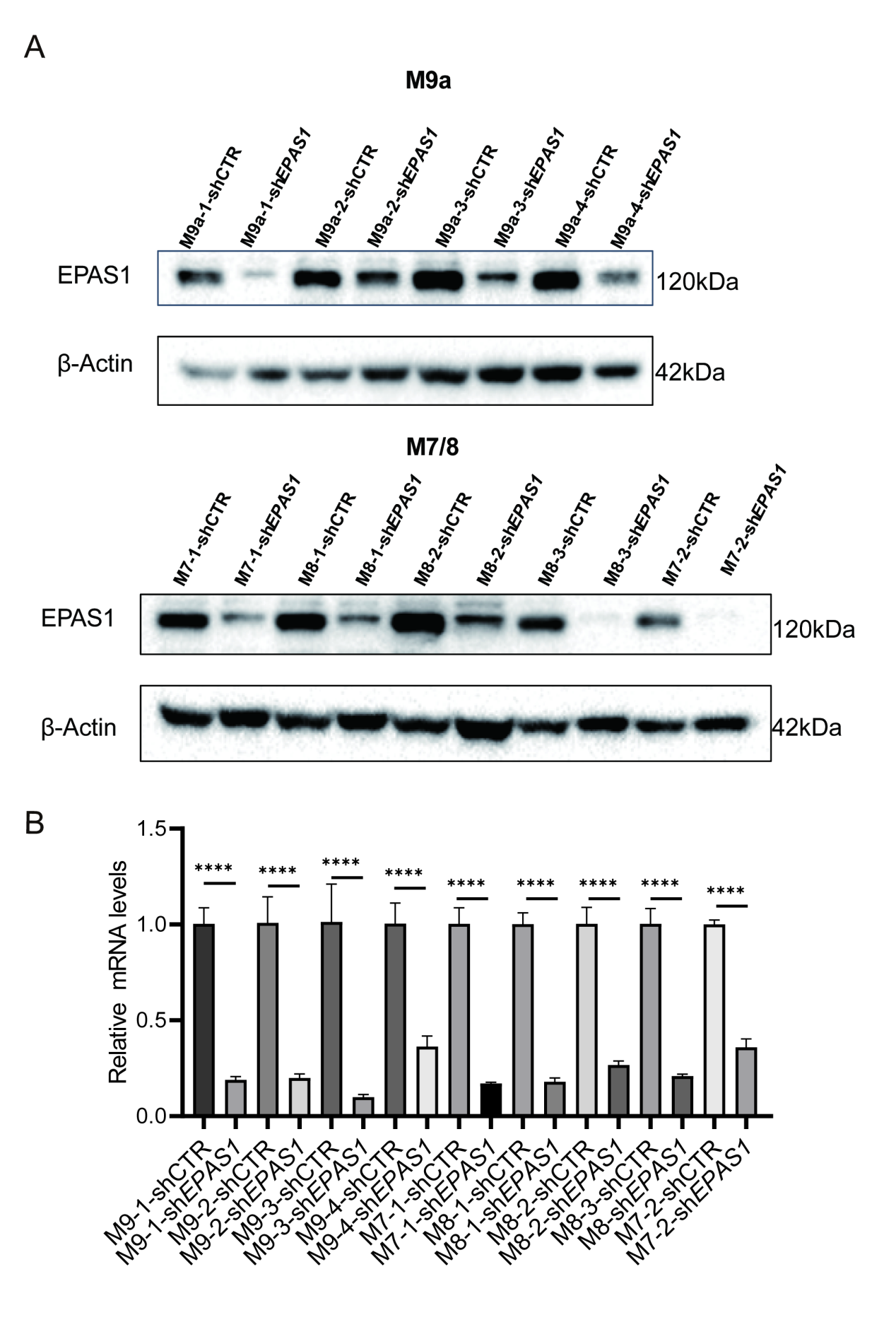


**Supplementary Fig.1. EPAS1 expression was significantly downregulated in sh*EPAS1* cells.**

The protein (A) and mRNA (B) expression of EPAS1 in M7/8/9a cybirds by treating with shRNA *EPAS1*. ACTIN was used as a loading control. Data are presented as the mean ± SD of three experiments. Two-tailed, unpaired Student’s t-tests; *P < 0.05; **P < 0.01, ***P < 0.001, ****P<0.0001.


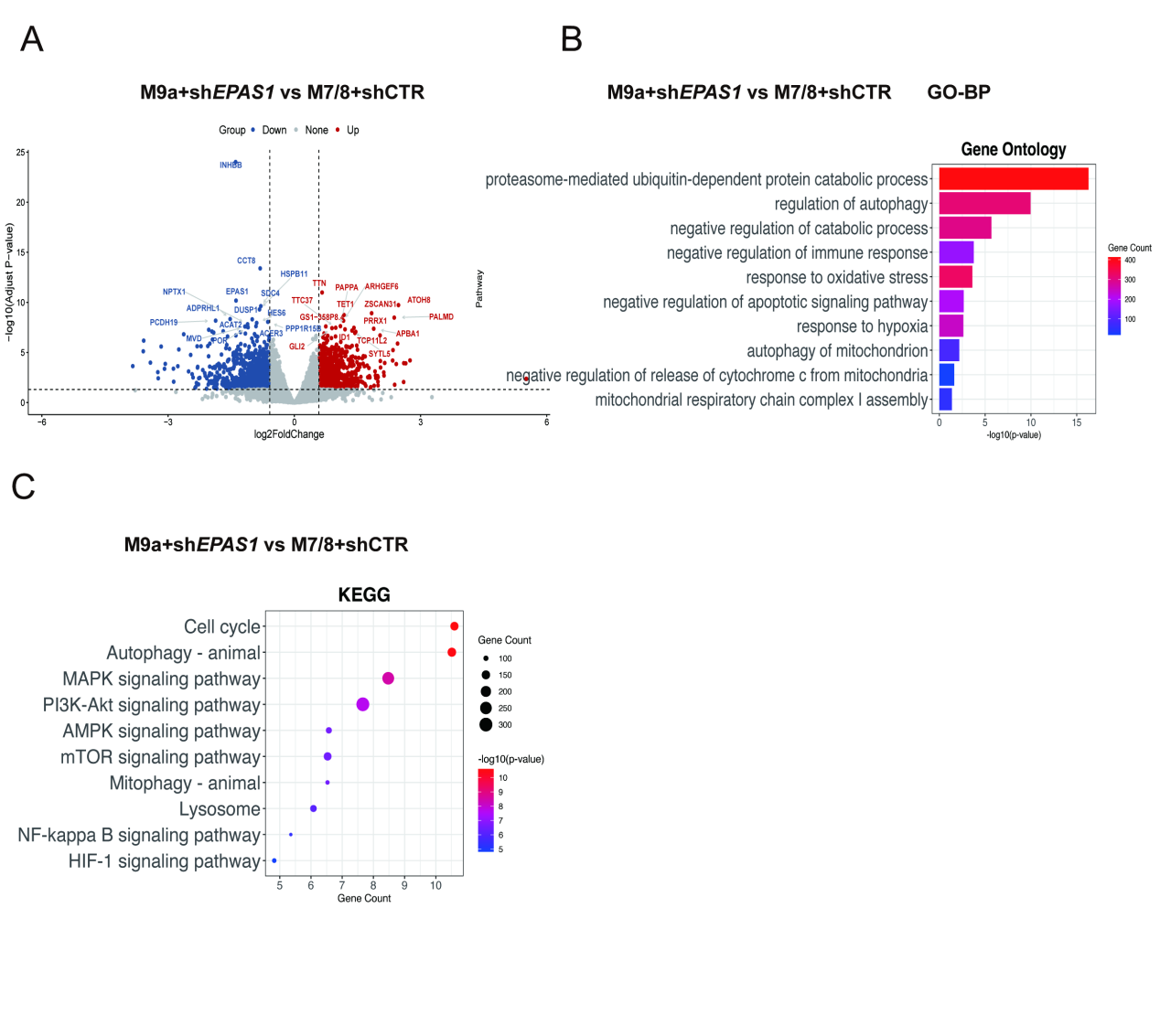


**Supplementary Fig.2. RNA-sequencing analysis of the M9a+sh*EPAS1* and M7/8+shCTR cells under hypoxia.**

The Volcano plot (A) , GO-BP (B) and KEGG (C) analysis of M9a+sh*EPAS1* VS M7/8+shCTR cells.


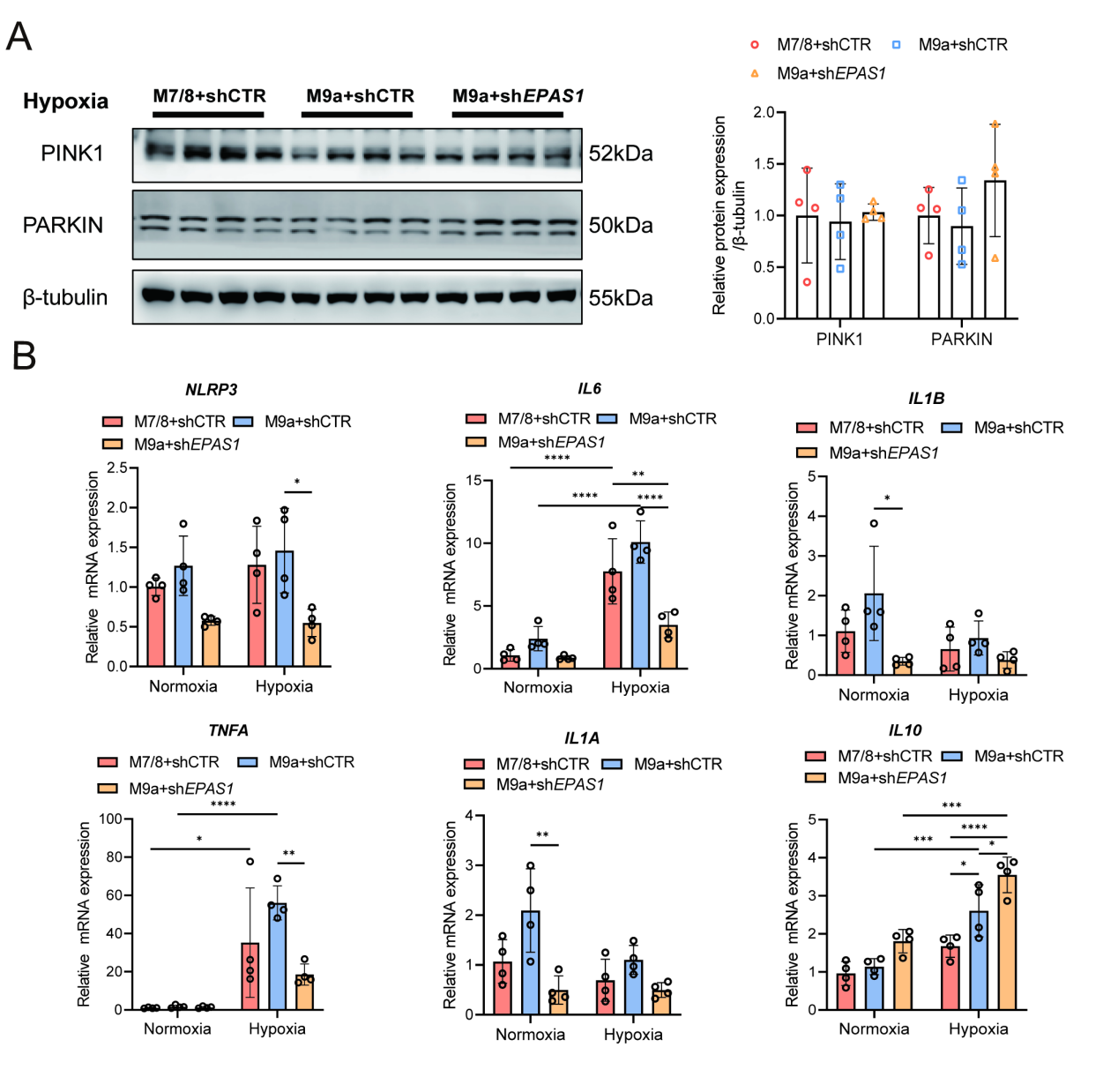


**Supplementary Fig.3. The protein expression levels of PINK1/PARKIN and the expression of inflammation-related genes were detected in three cell lines.**

(A):Western blot analysis of PINK1and PARKIN protein levels in the M7/8+shCTR (n = 4), M9a+shCTR (n = 4) and M9a+sh*EPAS1* (n = 4) cells under hypoxic condition. Grayscale value analysis of target protein levels were calibrated to β-Tubulin. (B):The mRNA expression of *NLRP3*, *IL6*, *IL1B*, *TNF*, *IL1A* and *IL10* were detected in the M7/8+shCTR (n = 4), M9a+shCTR (n = 4) and M9a+sh*EPAS1* (n = 4) cells under normoxia and hypoxic condition. ACTIN was used as control. Data are presented as the mean ± SD of three experiments. Two-way ANOVA followed by the Tukey test. *P < 0.05; **P < 0.01, ***P < 0.001, ****P<0.0001.


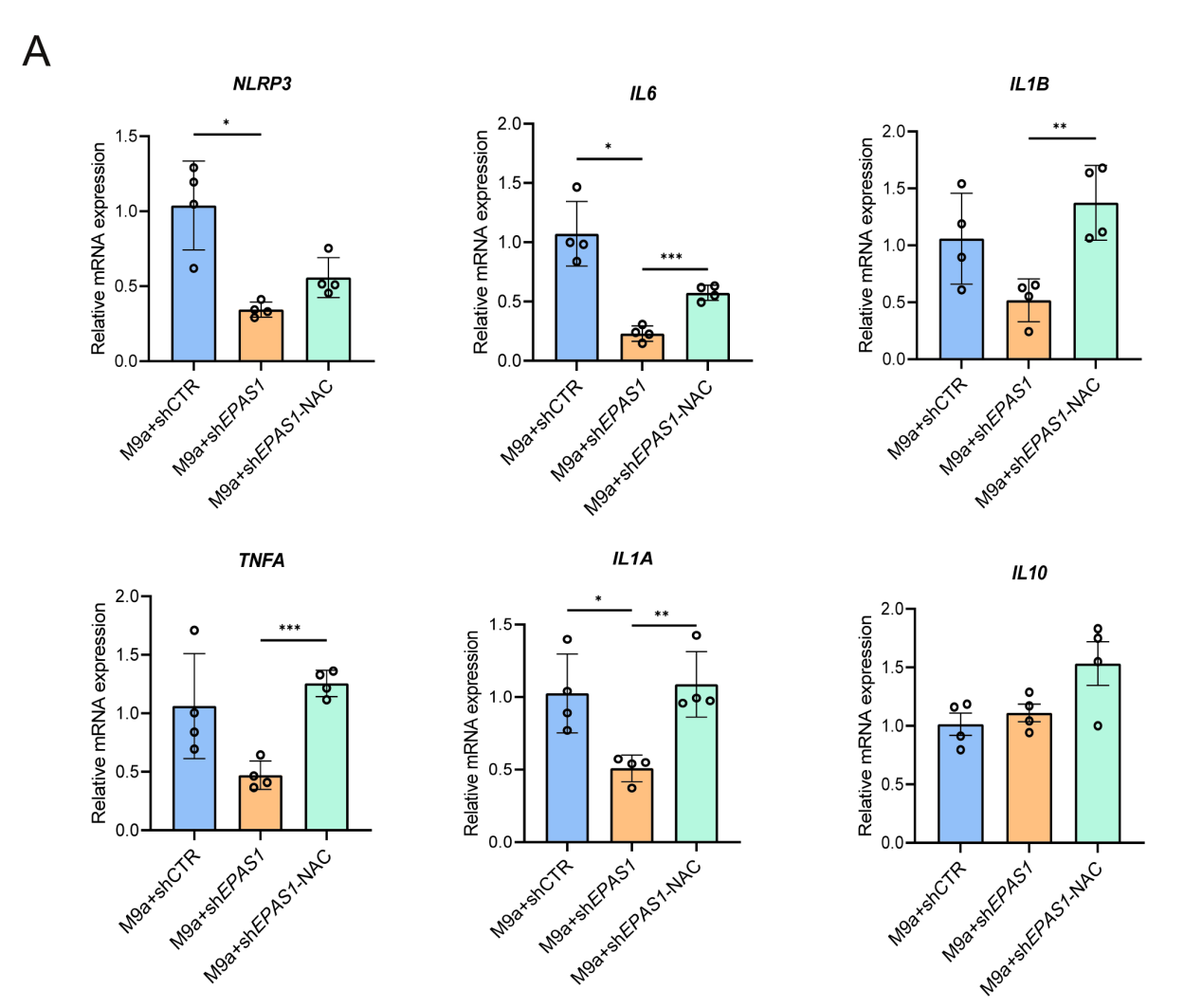


**Supplementary Fig.4. The levels of mtROS/total ROS and the expression of inflammation-related genes were detected under hypoxia.**

(A): The mRNA expression of *NLRP3*, *IL6*, *IL1B*, *TNF*, *IL1A* and *IL10* were detected in the M9a+shCTR (n = 4), M9a+sh*EPAS1* (n = 4) and M9a+sh*EPAS1*-NAC (n = 4) cells under normoxia and hypoxic condition. ACTIN was used as a control. Data are presented as the mean ± SD of three experiments. A-B: Two-way ANOVA followed by the Tukey’s test; C-E: One-way ANOVA followed by the Dunnett’s test. *P < 0.05; **P < 0.01, ***P < 0.001, ****P<0.0001.


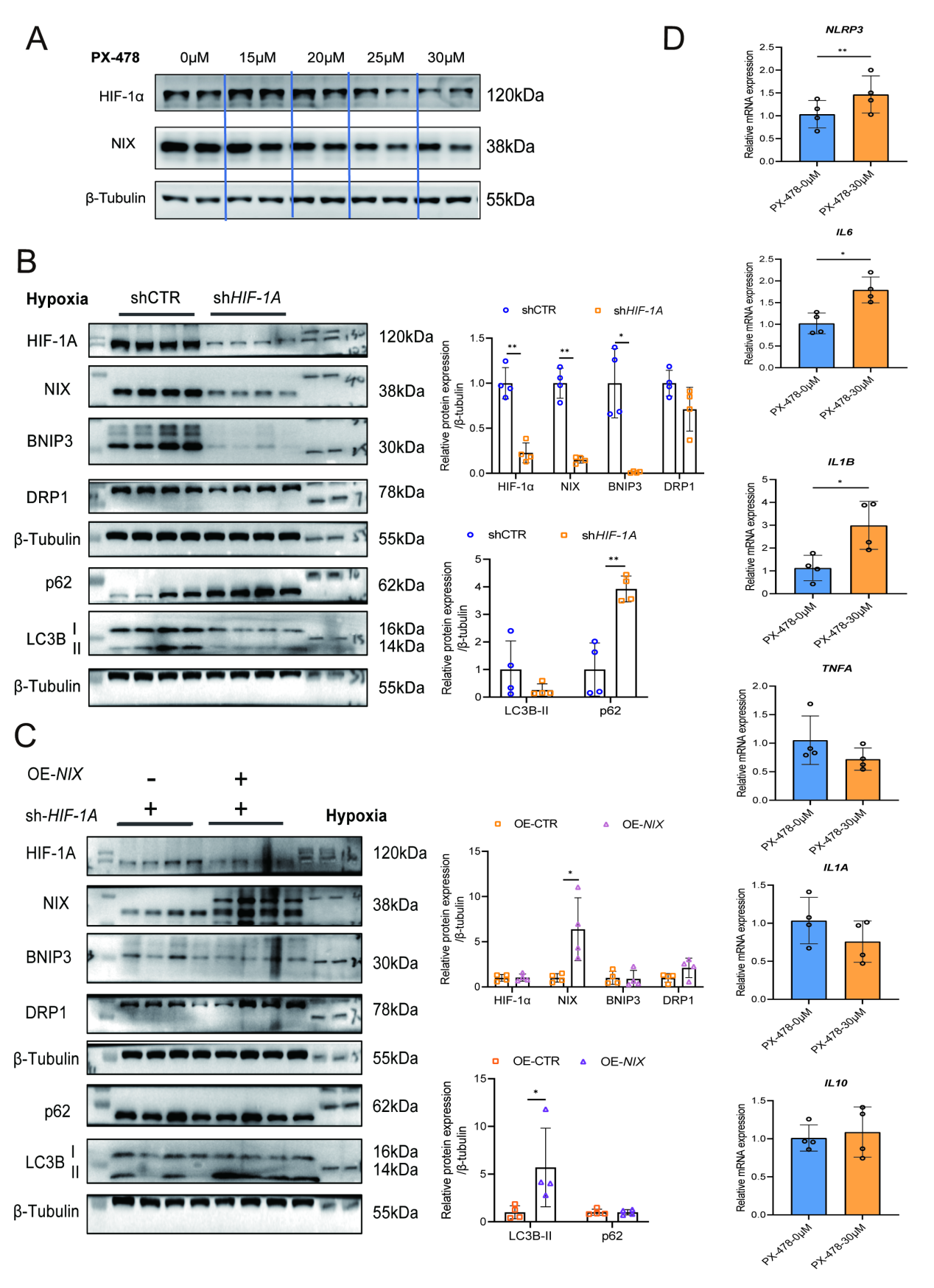


**Supplementary Fig.5**. **The role of the HIF-1α in mediating mitophagy and the inflammatory response under hypoxia.**

1. : Western blot analysis of HIF-1α and NIX protein levels in the M9a+sh*EPAS1* (n = 2) cells after treatment with PX-478 under hypoxic condition. Grayscale value analysis of target protein levels were calibrated to β-Tubulin. (B): Western blot analysis of mitophagy related protein  in the M9a+sh*EPAS1* (n = 4) cells after downregulation of HIF-1α under hypoxic condition. Grayscale value analysis of target protein levels were calibrated to β-Tubulin. (C): Western blot analysis of mitophagy-related proteins in M9a+shEPAS1 cells (n=4) upon HIF-1α knockdown and subsequent NIX overexpression under hypoxia. Band intensities were quantified and normalized to β-Tubulin. (D): The mRNA expression of *NLRP3*, *IL6*, *IL1B*, *TNF*, *IL1A* and *IL10* were detected in the M9a+sh*EPAS1* (n = 4) cells after treatment with PX-478 under hypoxic condition. ACTIN was used as a control. Data are presented as the mean ± SD of three experiments. Two-tailed, paired Student’s t-tests; *P < 0.05; **P < 0.01, ***P < 0.001, ****P<0.0001.

**
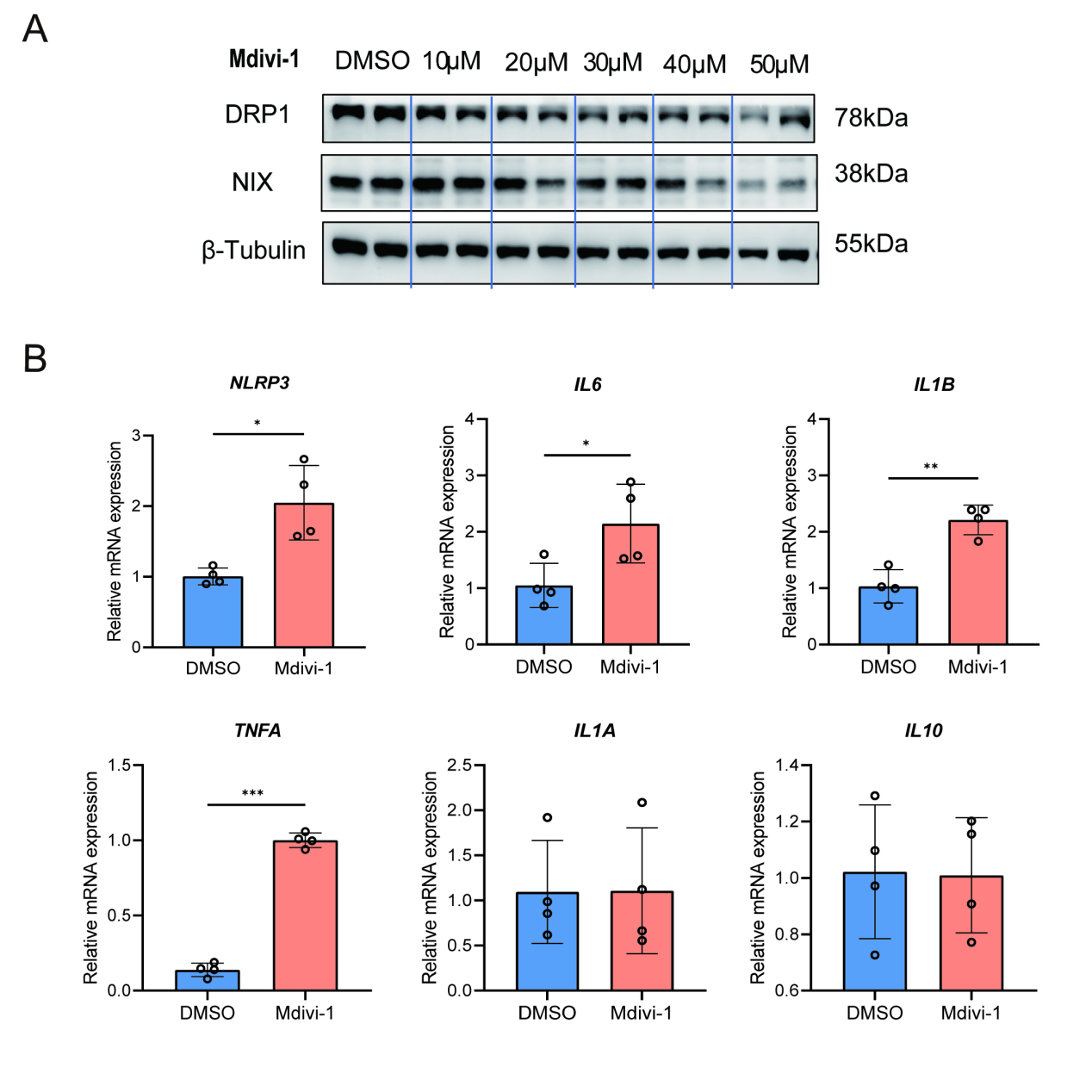
Supplementary Fig.6. The dose-dependent effect of Mdivi-1 and the expression of inflammation-related genes were detected after Mdivi-1 treatment under hypoxia.**

(A): Western blot analysis of DRP1 and NIX protein levels in the M9a+sh*EPAS1* (n = 2) cells after treatment with Mdivi-1 under hypoxic condition. Grayscale value analysis of target protein levels were calibrated to β-Tubulin. (B): The mRNA expression of *NLRP3*, *IL6*, *IL1B*, *TNF*, *IL1A* and *IL10* were detected in the M9a+sh*EPAS1* (n = 4) cells after treatment with Mdivi-1 under hypoxic condition. ACTIN was used as a control. Data are presented as the mean ± SD of three experiments. Two-tailed, paired Student’s t-tests; *P < 0.05; **P < 0.01, ***P < 0.001, ****P<0.0001.

**Supplementary Tables 1-13**

**Supplementary Table 1**-M9a cybrids

| position | gene | rCRS base | mutation | AA change | mtDNA database** |
| --- | --- | --- | --- | --- | --- |
| 73 | D-loop | A | G | no | polymorphic site |
| 263 | D-loop | A | G | no | polymorphic site |
| 489 | D-loop | T | C | no | polymorphic site |
| 750 | 12S rRNA | A | G | no | polymorphic site |
| 1041 | 12S rRNA | A | G | no | polymorphic site |
| 1438 | 12S rRNA | A | G | no | polymorphic site |
| 2416 | 16S rRNA | T | C | no | polymorphic site |
| 2706 | 16S rRNA | A | G | no | polymorphic site |
| 3394 | ND1 | T | C | Tyr > His | polymorphic site |
| 4491 | ND2 | G | A | Val > Ile | polymorphic site |
| 4769 | ND2 | A | G | no | polymorphic site |
| 7142 | COI | T | C | no | polymorphic site |
| 7697 | COII | G | A | Val > Ile | polymorphic site |
| 8701 | ATPase6 | A | G | Thr > Ala | polymorphic site |
| 8860 | ATPase6 | A | G | Thr > Ala | polymorphic site |
| 9098 | ATPase6 | T | C | Ile > Thr | polymorphic site |
| 9242 | COIII | A | G | no | polymorphic site |
| 9540 | COIII | T | C | no | polymorphic site |
| 10398 | ND3 | A | G | Thr > Ala | polymorphic site |
| 10400 | ND3 | C | T | Thr > Ala | polymorphic site |
| 10837 | ND4 | T | C | no | polymorphic site |
| 12705 | ND5 | C | T | no | polymorphic site |
| 14308 | ND6 | T | C | no | polymorphic site |
| 14417 | ND6 | A | G | Val > Ala | polymorphic site |
| 14766 | Cytb | C | T | Ile > Thr | polymorphic site |
| 14783 | Cytb | T | C | no | polymorphic site |
| 15043 | Cytb | G | A | no | polymorphic site |
| 15301 | Cytb | G | A | no | polymorphic site |
| 15326 | Cytb | A | G | Thr > Ala | polymorphic site |
| 16176 | D-loop | C | T | no | polymorphic site |
| 16223 | D-loop | C | T | no | polymorphic site |
| 16234 | D-loop | C | T | no | polymorphic site |
| 16311 | D-loop | T | C | no | polymorphic site |
| 16316 | D-loop | A | G | no | polymorphic site |
| 16362 | D-loop | T | C | no | polymorphic site |
| 16519 | D-loop | T | C | no | polymorphic site |

**Supplementary Table 2**-M9a cybrids

| position | gene | rCRS base | mutation | AA change | mtDNA database** |
| --- | --- | --- | --- | --- | --- |
| 73 | D-loop | A | G | no | polymorphic site |
| 263 | D-loop | A | G | no | polymorphic site |
| 489 | D-loop | T | C | no | polymorphic site |
| 711 | 12S rRNA | T | C | no | polymorphic site |
| 750 | 12S rRNA | A | G | no | polymorphic site |
| 1041 | 12S rRNA | A | G | no | polymorphic site |
| 1438 | 12S rRNA | A | G | no | polymorphic site |
| 2706 | 16S rRNA | A | G | no | polymorphic site |
| 3394 | ND1 | T | C | Tyr > His | polymorphic site |
| 4491 | ND2 | G | A | Val > Ile | polymorphic site |
| 4769 | ND2 | A | G | no | polymorphic site |
| 7028 | COI | C | T | no | polymorphic site |
| 7142 | COI | T | C | no | polymorphic site |
| 7697 | COII | G | A | Val > Ile | polymorphic site |
| 8701 | ATPase6 | A | G | Thr > Ala | polymorphic site |
| 8860 | ATPase6 | A | G | Thr > Ala | polymorphic site |
| 9242 | COIII | A | G | no | polymorphic site |
| 9261 | COIII | A | G | Thr > Ala | polymorphic site |
| 9540 | COIII | T | C | no | polymorphic site |
| 10205 | ND3 | C | T | Val > val | polymorphic site |
| 10398 | ND3 | A | G | Thr > Ala | polymorphic site |
| 10400 | ND3 | C | T | Thr > Ala | polymorphic site |
| 10873 | ND4 | T | C | no | polymorphic site |
| 11719 | ND4 | G | A | no | polymorphic site |
| 12705 | ND5 | C | T | no | polymorphic site |
| 14308 | ND6 | T | C | no | polymorphic site |
| 14417 | ND6 | A | G | Val > Ala | polymorphic site |
| 14766 | Cytb | C | T | Ile > Thr | polymorphic site |
| 14783 | Cytb | T | C | no | polymorphic site |
| 15043 | Cytb | G | A | no | polymorphic site |
| 15301 | Cytb | G | A | no | polymorphic site |
| 15326 | Cytb | A | G | Thr > Ala | polymorphic site |
| 16223 | D-loop | C | T | no | polymorphic site |
| 16234 | D-loop | C | T | no | polymorphic site |
| 16241 | D-loop | A | G | no | polymorphic site |
| 16316 | D-loop | A | G | no | polymorphic site |
| 16362 | D-loop | T | C | no | polymorphic site |

**Supplementary Table 3**-M9a cybrdis

| position | gene | rCRS base | mutation | AA change | mtDNA database** |
| --- | --- | --- | --- | --- | --- |
| 73 | D-loop | A | G | no | polymorphic site |
| 263 | D-loop | A | G | no | polymorphic site |
| 492 | D-loop | T | C | no | polymorphic site |
| 711 | 12S rRNA | T | C | no | polymorphic site |
| 750 | 12S rRNA | A | G | no | polymorphic site |
| 1041 | 12S rRNA | A | G | no | polymorphic site |
| 2706 | 16S rRNA | A | G | no | polymorphic site |
| 3394 | ND1 | T | C | Tyr > His | polymorphic site |
| 4491 | ND2 | G | A | Val > Ile | polymorphic site |
| 4769 | ND2 | A | G | no | polymorphic site |
| 7028 | COI | C | T | no | polymorphic site |
| 7142 | COI | T | C | no | polymorphic site |
| 7697 | COII | G | A | Val > Ile | polymorphic site |
| 8701 | ATPase6 | A | G | Thr > Ala | polymorphic site |
| 8860 | ATPase6 | A | G | Thr > Ala | polymorphic site |
| 9242 | COIII | A | G | no | polymorphic site |
| 9540 | COIII | T | C | no | polymorphic site |
| 10398 | ND3 | A | G | Thr > Ala | polymorphic site |
| 10400 | ND3 | C | T | Thr > Ala | polymorphic site |
| 10873 | ND4 | T | C | no | polymorphic site |
| 11719 | ND4 | G | A | no | polymorphic site |
| 12705 | ND5 | C | T | no | polymorphic site |
| 13015 | ND5 | T | C | no | polymorphic site |
| 14308 | ND6 | T | C | no | polymorphic site |
| 14417 | ND6 | A | G | Val > Ala | polymorphic site |
| 14766 | Cytb | C | T | Ile > Thr | polymorphic site |
| 14783 | Cytb | T | C | no | polymorphic site |
| 15043 | Cytb | G | A | no | polymorphic site |
| 15301 | Cytb | G | A | no | polymorphic site |
| 15326 | Cytb | A | G | Thr > Ala | polymorphic site |
| 16223 | D-loop | C | T | no | polymorphic site |
| 16234 | D-loop | C | T | no | polymorphic site |
| 16316 | D-loop | A | G | no | polymorphic site |
| 16362 | D-loop | T | C | no | polymorphic site |

**Supplementary Table 4**-M9a cybrids

| position | gene | rCRS base | mutation | AA change | mtDNA database** |
| --- | --- | --- | --- | --- | --- |
| 73 | D-loop | A | G | no | polymorphic site |
| 150 | D-loop | C | T | no | polymorphic site |
| 152 | D-loop | T | C | no | polymorphic site |
| 153 | D-loop | A | G | no | polymorphic site |
| 263 | D-loop | A | G | no | polymorphic site |
| 489 | D-loop | T | C | no | polymorphic site |
| 1041 | 12S rRNA | A | G | no | polymorphic site |
| 2706 | 16S rRNA | A | G | no | polymorphic site |
| 3394 | ND1 | T | C | Tyr > His | polymorphic site |
| 4491 | ND2 | G | A | Val > Ile | polymorphic site |
| 4769 | ND2 | A | G | no | polymorphic site |
| 7028 | COI | C | T | no | polymorphic site |
| 8701 | ATPase6 | A | G | Thr > Ala | polymorphic site |
| 8860 | ATPase6 | A | G | Thr > Ala | polymorphic site |
| 9540 | COIII | T | C | no | polymorphic site |
| 10398 | ND3 | A | G | Thr > Ala | polymorphic site |
| 10400 | ND3 | C | T | Thr > Ala | polymorphic site |
| 10454 | tRNA-Arg | T | C | no | polymorphic site |
| 10873 | ND4 | T | C | no | polymorphic site |
| 11719 | ND4 | G | A | no | polymorphic site |
| 12362 | ND5 | C | T | Thr > Ile | polymorphic site |
| 12705 | ND5 | C | T | no | polymorphic site |
| 14308 | ND6 | T | C | no | polymorphic site |
| 14766 | Cytb | C | T | Ile > Thr | polymorphic site |
| 14783 | Cytb | T | C | no | polymorphic site |
| 15043 | Cytb | G | A | no | polymorphic site |
| 15301 | Cytb | G | A | no | polymorphic site |
| 15326 | Cytb | A | G | Thr > Ala | polymorphic site |
| 16158 | D-loop | A | G | no | polymorphic site |
| 16223 | D-loop | C | T | no | polymorphic site |
| 16234 | D-loop | C | T | no | polymorphic site |
| 16265 | D-loop | A | C | no | polymorphic site |
| 16519 | D-loop | T | C | no | polymorphic site |

**Supplementary Table 5**-M9a cybrids

| position | gene | rCRS base | mutation | AA change | mtDNA database** |
| --- | --- | --- | --- | --- | --- |
| 73 | D-loop | A | G | no | polymorphic site |
| 150 | D-loop | C | T | no | polymorphic site |
| 152 | D-loop | T | C | no | polymorphic site |
| 153 | D-loop | A | G | no | polymorphic site |
| 263 | D-loop | A | G | no | polymorphic site |
| 489 | D-loop | T | C | no | polymorphic site |
| 750 | D-loop | A | G | no | polymorphic site |
| 1041 | 12S rRNA | A | G | no | polymorphic site |
| 2706 | 16S rRNA | A | G | no | polymorphic site |
| 3394 | ND1 | T | C | Tyr > His | polymorphic site |
| 4491 | ND2 | G | A | Val > Ile | polymorphic site |
| 4769 | ND2 | A | G | no | polymorphic site |
| 7028 | COI | C | T | no | polymorphic site |
| 8701 | ATPase6 | A | G | Thr > Ala | polymorphic site |
| 8860 | ATPase6 | A | G | Thr > Ala | polymorphic site |
| 10398 | ND3 | A | G | Thr > Ala | polymorphic site |
| 10400 | ND3 | C | T | Thr > Ala | polymorphic site |
| 10454 | tRNA-Arg | T | C | no | polymorphic site |
| 10873 | ND4 | T | C | no | polymorphic site |
| 11719 | ND4 | G | A | no | polymorphic site |
| 12362 | ND5 | C | T | Thr > Ile | polymorphic site |
| 12705 | ND5 | C | T | no | polymorphic site |
| 14308 | ND6 | T | C | no | polymorphic site |
| 14766 | Cytb | C | T | Ile > Thr | polymorphic site |
| 14783 | Cytb | T | C | no | polymorphic site |
| 15043 | Cytb | G | A | no | polymorphic site |
| 15301 | Cytb | G | A | no | polymorphic site |
| 15326 | Cytb | A | G | Thr > Ala | polymorphic site |
| 16158 | D-loop | A | G | no | polymorphic site |
| 16223 | D-loop | C | T | no | polymorphic site |
| 16234 | D-loop | C | T | no | polymorphic site |
| 16265 | D-loop | A | C | no | polymorphic site |
| 16362 | D-loop | T | C | no | polymorphic site |
| 16519 | D-loop | T | C | no | polymorphic site |

**Supplementary Table 6**-M7 cybrids

| position | gene | rCRS base | mutation | AA change | mtDNA database** |
| --- | --- | --- | --- | --- | --- |
| 73 | D-loop | A | G | no | polymorphic site |
| 150 | D-loop | C | T | no | polymorphic site |
| 199 | D-loop | T | C | no | polymorphic site |
| 204 | D-loop | T | C | no | polymorphic site |
| 263 | D-loop | A | G | no | polymorphic site |
| 489 | D-loop | T | C | no | polymorphic site |
| 750 | 12S rRNA | A | G | no | polymorphic site |
| 1438 | 12S rRNA | A | G | no | polymorphic site |
| 2706 | 16S rRNA | A | G | no | polymorphic site |
| 3483 | ND1 | G | A | no | polymorphic site |
| 4047 | ND1 | G | A | no | polymorphic site |
| 4048 | ND1 | G | A | Asp > Asn | polymorphic site |
| 4071 | ND1 | C | T | no | polymorphic site |
| 4164 | ND1 | A | G | no | polymorphic site |
| 4769 | ND2 | A | G | no | polymorphic site |
| 5351 | ND2 | A | G | no | polymorphic site |
| 5460 | ND2 | G | A | Ala > Thr | polymorphic site |
| 6455 | COI | C | T | Val > Ile | polymorphic site |
| 6680 | COI | T | C | no | polymorphic site |
| 7028 | COI | C | T | no | polymorphic site |
| 7598 | COII | G | A | Ala >Thr | polymorphic site |
| 7684 | COII | T | C | no | polymorphic site |
| 7853 | COII | G | A | Val > Ile | polymorphic site |
| 8572 | ATPase8 | G | A | no | polymorphic site |
| 8701 | ATPase6 | A | G | Thr > Ala | polymorphic site |
| 8860 | ATPase6 | A | G | Thr > Ala | polymorphic site |
| 9540 | COIII | T | C | no | polymorphic site |
| 9824 | COIII | T | C | no | polymorphic site |
| 10398 | ND3 | A | G | Thr > Ala | polymorphic site |
| 10400 | ND3 | C | T | Thr > Ala | polymorphic site |
| 10873 | ND4 | T | C | no | polymorphic site |
| 11719 | ND4 | G | A | no | polymorphic site |
| 12705 | ND5 | C | T | no | polymorphic site |
| 12811 | ND5 | T | C | Tyr > His | polymorphic site |
| 14766 | Cytb | C | T | Ile > Thr | polymorphic site |
| 14783 | Cytb | T | C | no | polymorphic site |
| 16223 | D-loop | C | T | no | polymorphic site |
| 16278 | D-loop | C | T | no | polymorphic site |
| 16297 | D-loop | T | C | no | polymorphic site |

**Supplementary Table 7**-M7 cybrids

| position | gene | rCRS base | mutation | AA change | mtDNA database** |
| --- | --- | --- | --- | --- | --- |
| 73 | D-loop | A | G | no | polymorphic site |
| 146 | D-loop | T | C | no | polymorphic site |
| 199 | D-loop | T | C | no | polymorphic site |
| 263 | D-loop | A | G | no | polymorphic site |
| 489 | D-loop | T | C | no | polymorphic site |
| 750 | 12S rRNA | A | G | no | polymorphic site |
| 1438 | 12S rRNA | A | G | no | polymorphic site |
| 2706 | 16S rRNA | A | G | no | polymorphic site |
| 3882 | ND1 | G | A | no | polymorphic site |
| 4071 | ND1 | C | T | no | polymorphic site |
| 4769 | ND2 | A | G | no | polymorphic site |
| 4850 | ND2 | C | T | no | polymorphic site |
| 5442 | ND2 | T | C | Phe > Leu | polymorphic site |
| 7028 | COI | C | T | no | polymorphic site |
| 8701 | ATPase6 | A | G | Thr > Ala | polymorphic site |
| 8860 | ATPase6 | A | G | Thr > Ala | polymorphic site |
| 9540 | COIII | T | C | no | polymorphic site |
| 9797 | COIII | T | C | no | polymorphic site |
| 9824 | COIII | T | C | no | polymorphic site |
| 10398 | ND3 | A | G | Thr > Ala | polymorphic site |
| 10400 | ND3 | C | T | Thr > Ala | polymorphic site |
| 10873 | ND4 | T | C | no | polymorphic site |
| 11665 | ND4 | C | T | no | polymorphic site |
| 11719 | ND4 | G | A | no | polymorphic site |
| 11815 | ND4 | C | A | no | polymorphic site |
| 12091 | ND4 | T | C | no | polymorphic site |
| 12705 | ND5 | C | T | no | polymorphic site |
| 14766 | Cytb | C | T | Ile > Thr | polymorphic site |
| 14783 | Cytb | T | C | no | polymorphic site |
| 15043 | Cytb | G | A | no | polymorphic site |
| 15301 | Cytb | G | A | no | polymorphic site |
| 15326 | Cytb | A | G | Thr > Ala | polymorphic site |
| 15944 | tRNA-Pro | T | C | no | polymorphic site |
| 16172 | D-loop | T | C | no | polymorphic site |
| 16173 | D-loop | C | T | no | polymorphic site |
| 16223 | D-loop | C | T | no | polymorphic site |
| 16295 | D-loop | C | T | no | polymorphic site |
| 16362 | D-loop | T | C | no | polymorphic site |
| 16401 | D-loop | C | T | no | polymorphic site |
| 16519 | D-loop | T | C | no | polymorphic site |

**Supplementary Table 8**-M7 cybrids

| position | gene | rCRS base | mutation | AA change | mtDNA database** |
| --- | --- | --- | --- | --- | --- |
| 73 | D-loop | A | G | no | polymorphic site |
| 150 | D-loop | C | T | no | polymorphic site |
| 199 | D-loop | T | C | no | polymorphic site |
| 204 | D-loop | T | C | no | polymorphic site |
| 263 | D-loop | A | G | no | polymorphic site |
| 489 | D-loop | T | C | no | polymorphic site |
| 750 | 12S rRNA | A | G | no | polymorphic site |
| 1438 | 12S rRNA | A | G | no | polymorphic site |
| 2706 | 16S rRNA | A | G | no | polymorphic site |
| 4048 | ND1 | G | A | Asp > Asn | polymorphic site |
| 4071 | ND1 | C | T | no | polymorphic site |
| 4164 | ND1 | A | G | no | polymorphic site |
| 4769 | ND2 | A | G | no | polymorphic site |
| 5460 | ND2 | G | A | Ala > Thr | polymorphic site |
| 6680 | COI | T | C | no | polymorphic site |
| 7028 | COI | C | T | no | polymorphic site |
| 7684 | COII | T | C | no | polymorphic site |
| 7853 | COII | G | A | Val > Ile | polymorphic site |
| 8701 | ATPase6 | A | G | Thr > Ala | polymorphic site |
| 8860 | ATPase6 | A | G | Thr > Ala | polymorphic site |
| 9540 | COIII | T | C | no | polymorphic site |
| 9824 | COIII | T | C | no | polymorphic site |
| 10398 | ND3 | A | G | Thr > Ala | polymorphic site |
| 10400 | ND3 | C | T | Thr > Ala | polymorphic site |
| 10873 | ND4 | T | C | no | polymorphic site |
| 11719 | ND4 | G | A | no | polymorphic site |
| 12705 | ND5 | C | T | no | polymorphic site |
| 12811 | ND5 | T | C | Tyr > His | polymorphic site |
| 14256 | ND6 | T | C | no | polymorphic site |
| 14766 | Cytb | C | T | Ile > Thr | polymorphic site |
| 14783 | Cytb | T | C | no | polymorphic site |
| 15043 | Cytb | G | A | no | polymorphic site |
| 15301 | Cytb | G | A | no | polymorphic site |
| 15326 | Cytb | A | G | Thr > Ala | polymorphic site |
| 16129 | D-loop | G | A | D-loop | polymorphic site |
| 16192 | D-loop | C | T | D-loop | polymorphic site |
| 16223 | D-loop | C | T | D-loop | polymorphic site |
| 16291 | D-loop | C | T | D-loop | polymorphic site |
| 16297 | D-loop | T | C | D-loop | polymorphic site |
| 16519 | D-loop | T | C | D-loop | polymorphic site |

**Supplementary Table 9**-M8 cybrids

| position | gene | rCRS base | mutation | AA change | mtDNA database** |
| --- | --- | --- | --- | --- | --- |
| 73 | D-loop | A | G | no | polymorphic site |
| 199 | D-loop | T | C | no | polymorphic site |
| 204 | D-loop | T | C | no | polymorphic site |
| 263 | D-loop | A | G | no | polymorphic site |
| 489 | D-loop | T | C | no | polymorphic site |
| 750 | 12S rRNA | A | G | no | polymorphic site |
| 2706 | 16S rRNA | A | G | no | polymorphic site |
| 2835 | 16S rRNA | C | T | no | polymorphic site |
| 4715 | ND2 | A | G | no | polymorphic site |
| 4769 | ND2 | A | G | no | polymorphic site |
| 6179 | COI | G | A | no | polymorphic site |
| 7028 | COI | C | T | no | polymorphic site |
| 7196 | COI | C | A | no | polymorphic site |
| 8486 | ATPase6 | C | T | Pro > Ser | polymorphic site |
| 8584 | ATPase6 | G | A | Ala > Thr | polymorphic site |
| 8684 | ATPase6 | C | T | Thr > Ile | polymorphic site |
| 8701 | ATPase6 | A | G | no | polymorphic site |
| 8860 | ATPase6 | A | G | no | polymorphic site |
| 9540 | COIII | T | C | no | polymorphic site |
| 10398 | ND3 | A | G | Thr > Ala | polymorphic site |
| 10400 | ND3 | C | T | Thr > Ala | polymorphic site |
| 10873 | ND4 | T | C | no | polymorphic site |
| 11719 | ND4 | G | A | no | polymorphic site |
| 12705 | ND5 | C | T | no | polymorphic site |
| 14470 | ND6 | T | C | no | polymorphic site |
| 14766 | Cytb | C | T | Ile > Thr | polymorphic site |
| 14783 | Cytb | T | C | no | polymorphic site |
| 15043 | Cytb | G | A | no | polymorphic site |
| 15244 | Cytb | A | G | no | polymorphic site |
| 15301 | Cytb | G | A | no | polymorphic site |
| 15326 | Cytb | A | G | Thr > Ala | polymorphic site |
| 15487 | Cytb | A | T | no | polymorphic site |
| 16131 | D-loop | T | C | no | polymorphic site |
| 16184 | D-loop | C | T | no | polymorphic site |
| 16223 | D-loop | C | T | no | polymorphic site |
| 16271 | D-loop | T | C | no | polymorphic site |
| 16298 | D-loop | T | C | no | polymorphic site |
| 16319 | D-loop | G | A | no | polymorphic site |

**Supplementary Table 10**-M8 cybrids

| position | gene | rCRS base | mutation | AA change | mtDNA database** |
| --- | --- | --- | --- | --- | --- |
| 73 | D-loop | A | G | no | polymorphic site |
| 263 | D-loop | A | G | no | polymorphic site |
| 489 | D-loop | T | C | no | polymorphic site |
| 750 | 12S rRNA | A | G | no | polymorphic site |
| 1438 | 12S rRNA | A | G | no | polymorphic site |
| 2706 | 16S rRNA | A | G | no | polymorphic site |
| 2835 | 16S rRNA | C | T | no | polymorphic site |
| 4670 | ND2 | C | T | no | polymorphic site |
| 4715 | ND2 | A | G | no | polymorphic site |
| 4769 | ND2 | A | G | no | polymorphic site |
| 6179 | COI | G | A | no | polymorphic site |
| 6671 | COI | T | C | no | polymorphic site |
| 7028 | COI | C | T | no | polymorphic site |
| 7196 | COI | C | A | no | polymorphic site |
| 8584 | ATPase6 | G | A | Ala > Thr | polymorphic site |
| 8684 | ATPase6 | C | T | Thr > Ile | polymorphic site |
| 8701 | ATPase6 | A | G | no | polymorphic site |
| 8860 | ATPase6 | A | G | Thr > Ala | polymorphic site |
| 9540 | COIII | T | C | no | polymorphic site |
| 10398 | ND3 | A | G | Thr > Ala | polymorphic site |
| 10400 | ND3 | C | T | Thr > Ala | polymorphic site |
| 10873 | ND4 | T | C | no | polymorphic site |
| 11255 | ND4 | T | C | Tyr > His | polymorphic site |
| 11719 | ND4 | G | A | no | polymorphic site |
| 12705 | ND5 | C | T | no | polymorphic site |
| 13050 | ND5 | A | G | no | polymorphic site |
| 14470 | ND6 | T | C | no | polymorphic site |
| 14766 | Cytb | C | T | Ile > Thr | polymorphic site |
| 14783 | Cytb | T | C | no | polymorphic site |
| 15043 | Cytb | G | A | no | polymorphic site |
| 15301 | Cytb | G | A | no | polymorphic site |
| 15326 | Cytb | A | G | Thr > Ala | polymorphic site |
| 15487 | Cytb | A | T | no | polymorphic site |
| 16153 | D-loop | G | A | no | polymorphic site |
| 16184 | D-loop | C | T | no | polymorphic site |
| 16223 | D-loop | C | T | no | polymorphic site |
| 16298 | D-loop | T | C | no | polymorphic site |
| 16319 | D-loop | G | A | no | polymorphic site |
| 16407 | D-loop | C | T | no | polymorphic site |

**Supplementary Table 11**-M8 cybrids

| position | gene | rCRS base | mutation | AA change | mtDNA database** |
| --- | --- | --- | --- | --- | --- |
| 73 | D-loop | A | G | no | polymorphic site |
| 263 | D-loop | A | G | no | polymorphic site |
| 1438 | 12S rRNA | A | G | no | polymorphic site |
| 2706 | 16S rRNA | A | G | no | polymorphic site |
| 4715 | ND2 | A | G | no | polymorphic site |
| 4769 | ND2 | A | G | no | polymorphic site |
| 4841 | ND2 | G | A | no | polymorphic site |
| 6179 | COI | G | A | no | polymorphic site |
| 7028 | COI | C | T | no | polymorphic site |
| 7196 | COI | C | A | no | polymorphic site |
| 8584 | ATPase6 | G | A | Ala > Thr | polymorphic site |
| 8684 | ATPase6 | C | T | Thr > Ile | polymorphic site |
| 8701 | ATPase6 | A | G | Thr > Ala | polymorphic site |
| 8860 | ATPase6 | A | G | Thr > Ala | polymorphic site |
| 9540 | COIII | T | C | no | polymorphic site |
| 9548 | COIII | G | A | no | polymorphic site |
| 10398 | ND3 | A | G | Thr > Ala | polymorphic site |
| 10400 | ND3 | C | T | Thr > Ala | polymorphic site |
| 10873 | ND4 | T | C | no | polymorphic site |
| 11719 | ND4 | G | A | no | polymorphic site |
| 13488 | ND5 | T | C | no | polymorphic site |
| 14470 | ND6 | T | C | no | polymorphic site |
| 14766 | Cytb | C | T | Ile > Thr | polymorphic site |
| 14783 | Cytb | T | C | no | polymorphic site |
| 15043 | Cytb | G | A | no | polymorphic site |
| 15301 | Cytb | G | A | no | polymorphic site |
| 15326 | Cytb | A | G | Thr > Ala | polymorphic site |
| 16184 | D-loop | C | T | no | polymorphic site |
| 16223 | D-loop | C | T | no | polymorphic site |
| 16245 | D-loop | C | T | no | polymorphic site |
| 16293 | D-loop | A | G | no | polymorphic site |
| 16298 | D-loop | T | C | no | polymorphic site |
| 16319 | D-loop | G | A | no | polymorphic site |

rCRS：revised Cambridge Reference Sequence

AA：Amino Acid

**databases：By searching the MITOMAP、mtSNP 、mtDB and Phylo Tree databases, we excluded the influence of reported pathogenic mutation sites on the results.

**Supplementary Table 12.** List of primers used in RT-PCR of the study.

| Gene names | Sequences (5→3’) |
| --- | --- |
| Human *EPAS1*-F | CTGTGTCTGAGAAGAGTAACTTCC |
| Human *EPAS1*-R | TTGCCATAGGCTGAGGACTCCT |
| Human *NLRP3*-F | GGACTGAAGCACCTGTTGTGCA |
| Human *NLRP3*-R | TCCTGAGTCTCCCAAGGCATTC |
| Human *IL6*-F | AGACAGCCACTCACCTCTTCAG |
| Human *IL6*-R | TTCTGCCAGTGCCTCTTTGCTG |
| Human *IL1B*-F | CCACAGACCTTCCAGGAGAATG |
| Human *IL1B*-R | GTGCAGTTCAGTGATCGTACAGG |
| Human *TNF*-F | CTCTTCTGCCTGCTGCACTTTG |
| Human *TNF*-R | ATGGGCTACAGGCTTGTCACTC |
| Human *IL1A*-F | TGTATGTGACTGCCCAAGATGAAG |
| Human *IL1A*-R | AGAGGAGGTTGGTCTCACTACC |
| Human *IL10*-F | TCTCCGAGATGCCTTCAGCAGA |
| Human *IL10*-R | TCAGACAAGGCTTGGCAACCCA |
| Human *β-ACTIN*-F | CACCATTGGCAATGAGCGGTTC |
| Human *β-ACTIN*-R | AGGTCTTTGCGGATGTCCACGT |

**Supplementary Table 13.** List of antibody used in this study.

| Antigen specificity | Species | Dilution | Application | Supplier | Reference |
| --- | --- | --- | --- | --- | --- |
| EPAS1 | Rabbit | 1:1000 | WB | NOVUS | NB100-122 |
| HIF-1α | Rabbit | 1:1000 | WB | Cell Signaling | 36169S |
| BNIP3 | Mouse | 1:1000 | WB | [Santa Cruz](https://www.bing.com/ck/a?!&&p=378e5c325153e77bJmltdHM9MTcyMTc3OTIwMCZpZ3VpZD0wZTkxZmE2OS1hODBmLTY3MTUtMzY1My1lOWMyYTlkZDY2ZDQmaW5zaWQ9NTE5OQ&ptn=3&ver=2&hsh=3&fclid=0e91fa69-a80f-6715-3653-e9c2a9dd66d4&psq=santa+cruz&u=a1aHR0cHM6Ly93d3cuc2NidC5jb20vemgvaG9tZQ&ntb=1) | sc-56167 |
| NIX | Rabbit | 1:1000 | WB | Cell Signaling | 12396S |
| LC3B | Rabbit | 1:1000 | WB | Cell Signaling | 3868S |
| p62 | Rabbit | 1:1000 | WB | Cell Signaling | 39749 |
| MFN1 | Rabbit | 1:1000 | WB | Proteintech | 13798-1-AP |
| DRP1 | Rabbit | 1:1000 | WB | Proteintech | 12957-1-AP |
| C-Caspase-3 | Rabbit | 1:1000 | WB | HUABIO | ET1602-47 |
| BAX | Rabbit | 1:1000 | WB | Cell Signaling | 2772S |
| PINK1 | Rabbit | 1:1000 | WB | Proteintech | 23274-1-AP |
| PARKIN | Rabbit | 1:1000 | WB | Abmart | [T56641](http://www.ab-mart.com.cn/page.aspx?node= 77 &id= 2309" \t "https://www.ab-mart.com.cn/_blank) |
| NLRP3 | Rabbit | 1:1000 | WB | Cell Signaling | 15101S |
| IL-1β | Rabbit | 1:1000 | WB | Abmart | P50520-1R1S |
| cGAS | Rabbit | 1:1000 | WB | Proteintech | 26416-1-AP |
| STING | Rabbit | 1:1000 | WB | Proteintech | 19851-1-AP |
| P65 | Rabbit | 1:1000 | WB | Cell Signaling | 8242S |
| P-P65 | Rabbit | 1:1000 | WB | Cell Signaling | 3033S |
| β-Tubulin | Rabbit | 1:1000 | WB | Cell Signaling | 2146S |
| β-Actin | Rabbit | 1:1000 | WB | Cell Signaling | 4967S |
| GRIM19 | Mouse | 1:1000 | BNG | Abcam | ab110240 |
| SDHA | Mouse | 1:1000 | BNG | Abcam | ab14715 |
| UQCRC2 | Mouse | 1:1000 | BNG | Abcam | ab14745 |
| COX1 | Mouse | 1:1000 | BNG | Abcam | ab109025 |
| ATP5A | Mouse | 1:1000 | BNG | Abcam | ab14748 |
| VDAC | Rabbit | 1:1000 | WB | Abcam | ab154856 |
| dsDNA | Mouse | 1:100 | IF | Abcam | ab27156 |
| LC3B | Rabbit | 1:100 | IF | Cell Signaling | 3868S |
| [Goat Anti-Rabbit IgG H&L (Alexa Fluor® 488)](https://www.abcam.cn/products/secondary-antibodies/goat-rabbit-igg-hl-alexa-fluor-488-ab150077.html) | - | 1:400 | IF | Abcam | ab150077 |
| Goat Anti-Mouse IgG (H+L) Fluor488-conjugated | - | 1:400 | IF | Affinity Biosciences | S0017 |
| HRP Goat anti-Rabbit | - | 1:2000 | WB | Cell Signaling | 7074S |
| HRP Goat anti-Mouse | - | 1:2000 | WB | Cell Signaling | 7076S |

IF: immunofluorescence; WB: western blot.
