# Supplementary material for: Mitochondrial retrograde signaling initiates HIF-1α/BNIP3/NIX-mediated mitophagy in Tibetan high-altitude adaptation
Source: Cell Death Discov. 2026 Jan 6;12:81. doi: 10.1038/s41420-025-02933-8 (PMC12877009; doi:10.1038/s41420-025-02933-8)

Western blot original images of the main article and supplementary materials

Note：

Due to issues with the previous imaging instrument, some bands in the initial results lacked molecular markers. Subsequently, we re-ran all main experiments with a new instrument to generate biologically replicated bands, ensuring both loading controls and target bands appear on the same membrane.

All representative blots from the manuscript

Figure 1

Figure 1A -Normoxia

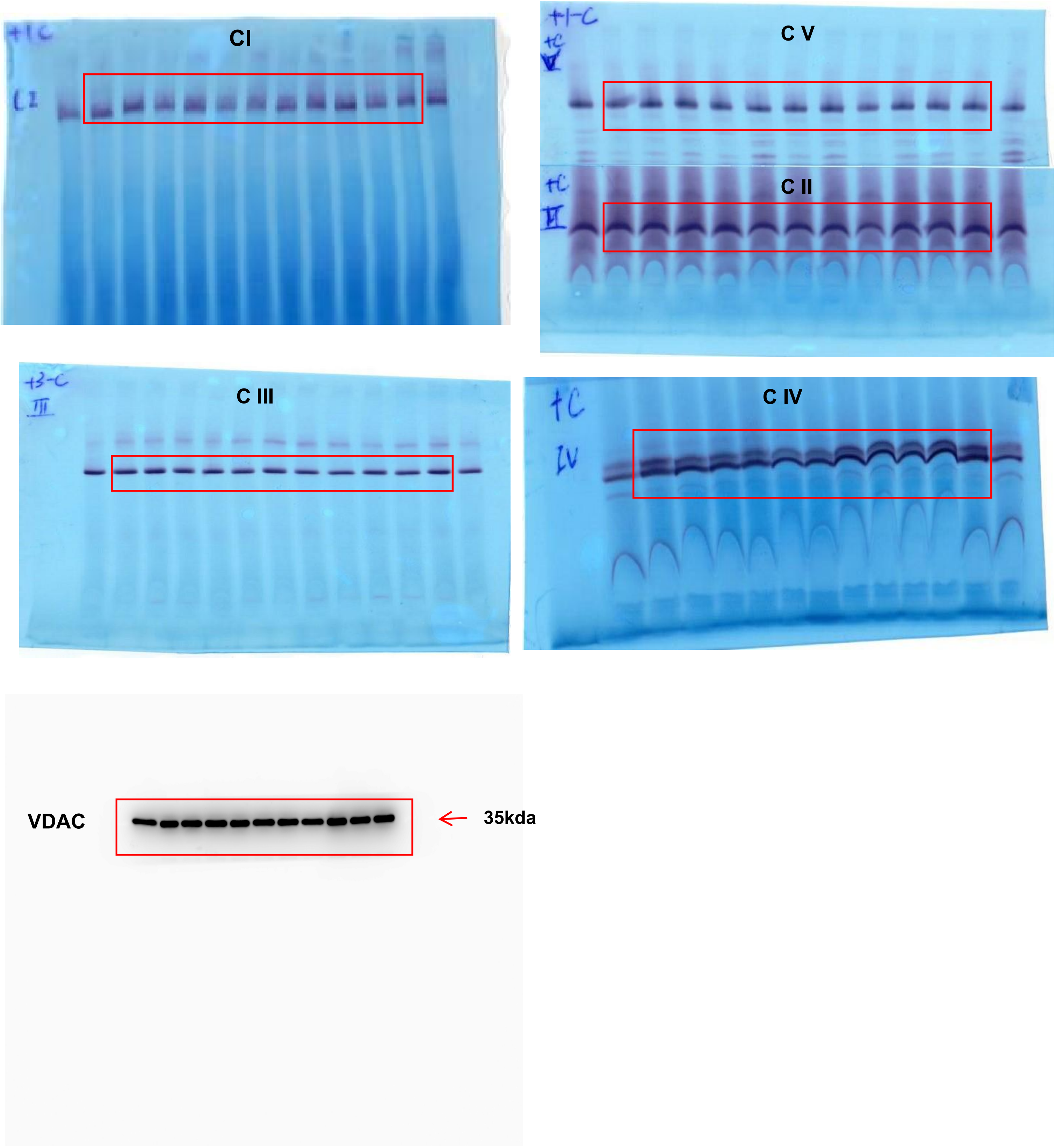

Figure 1

Figure 1A -Hypoxia

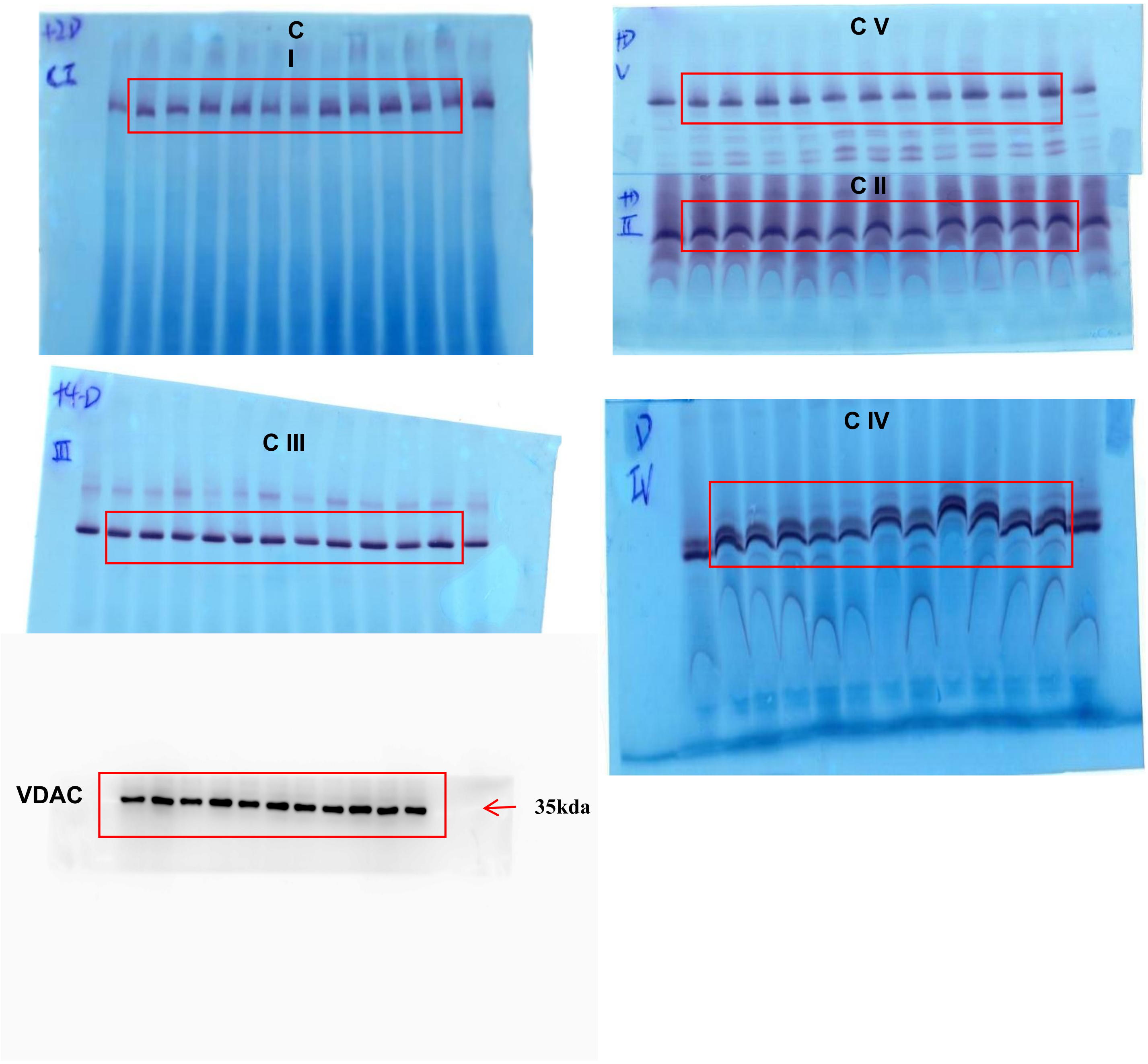

Figure 1

Figure 1B -Normoxia

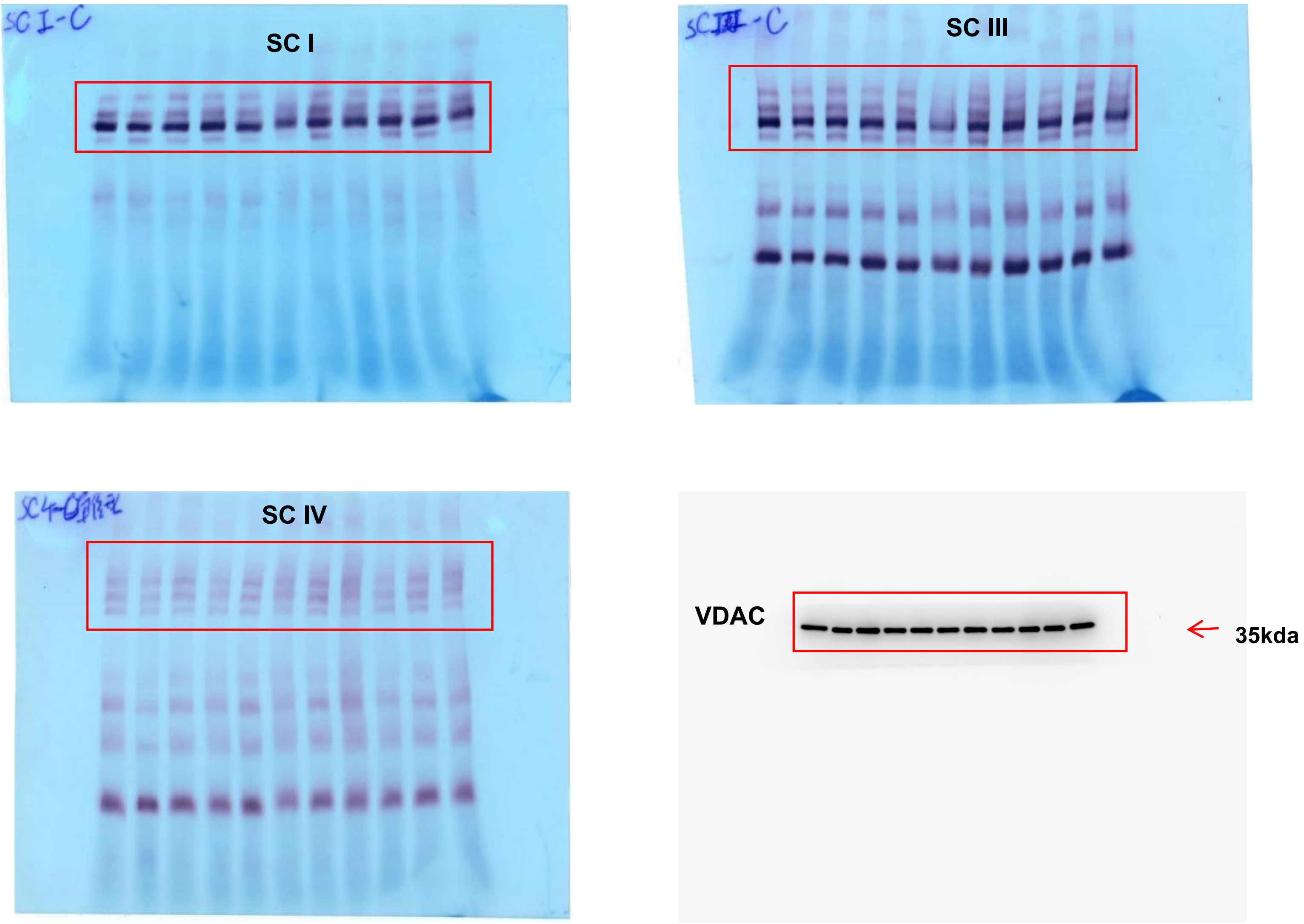

Figure 1

Figure 1B -Hypoxia

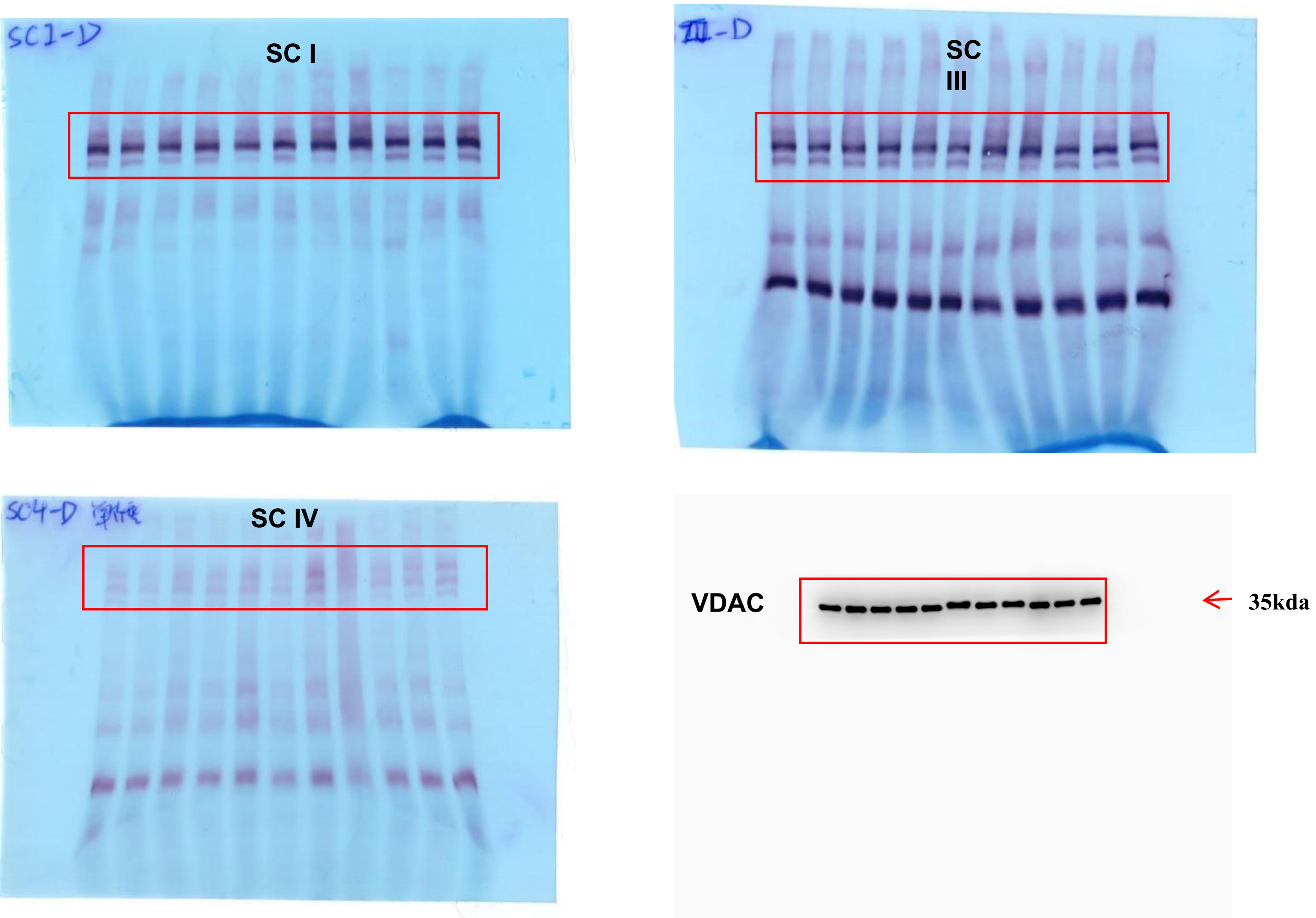

Figure 2

Figure 2K

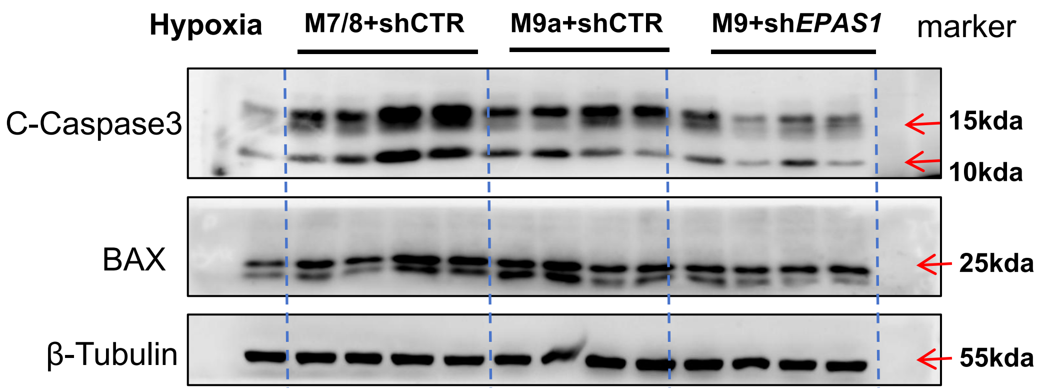

Figure 4

Figure 4A

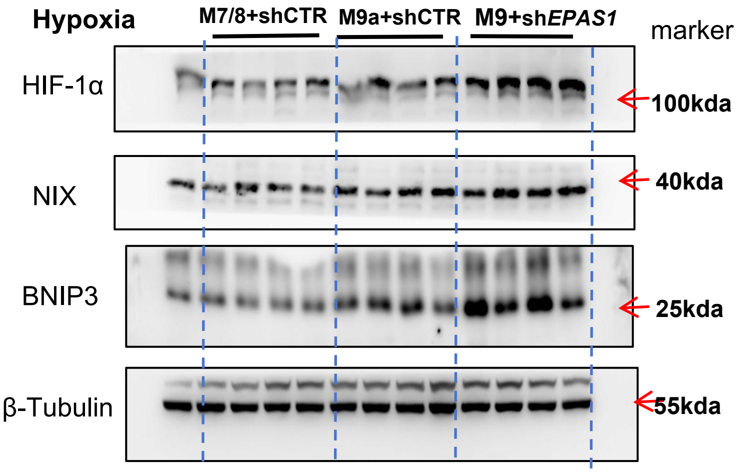

Figure 4B

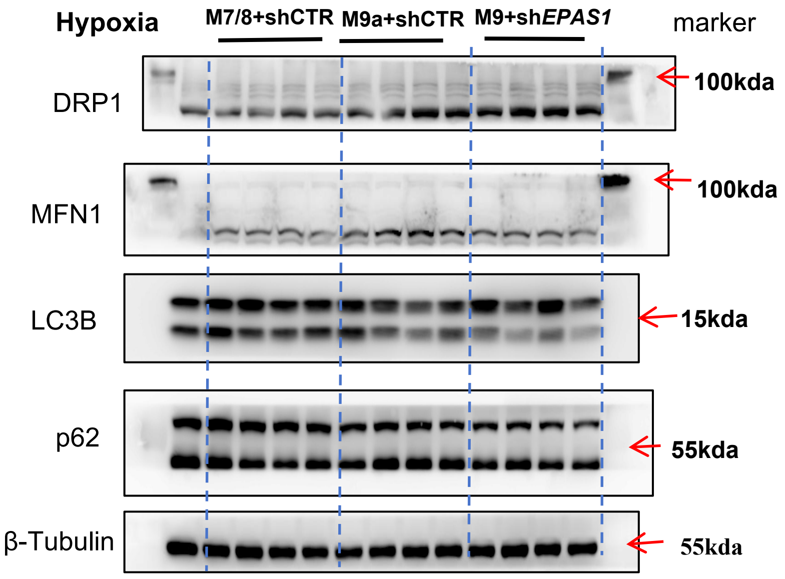

Figure 4F

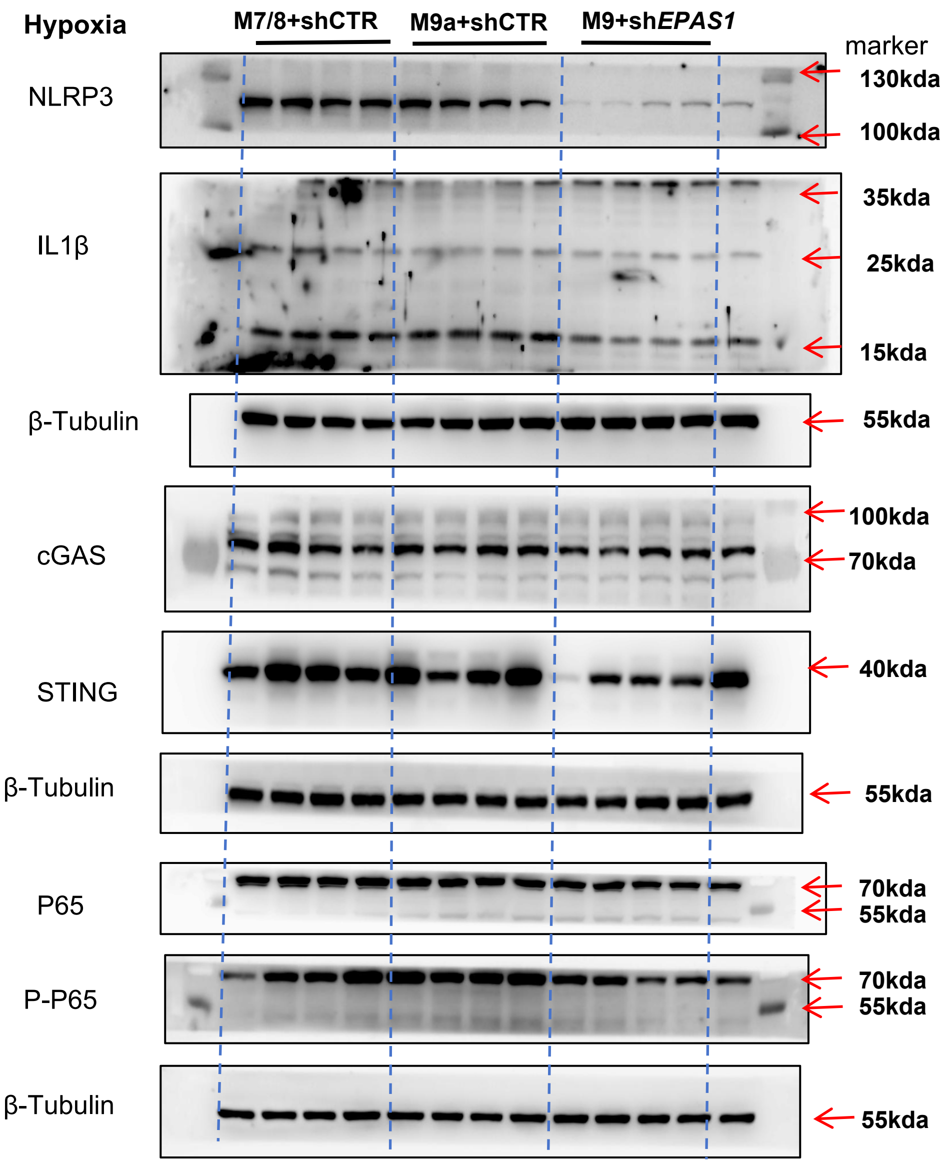

Figure 5

Figure 5B

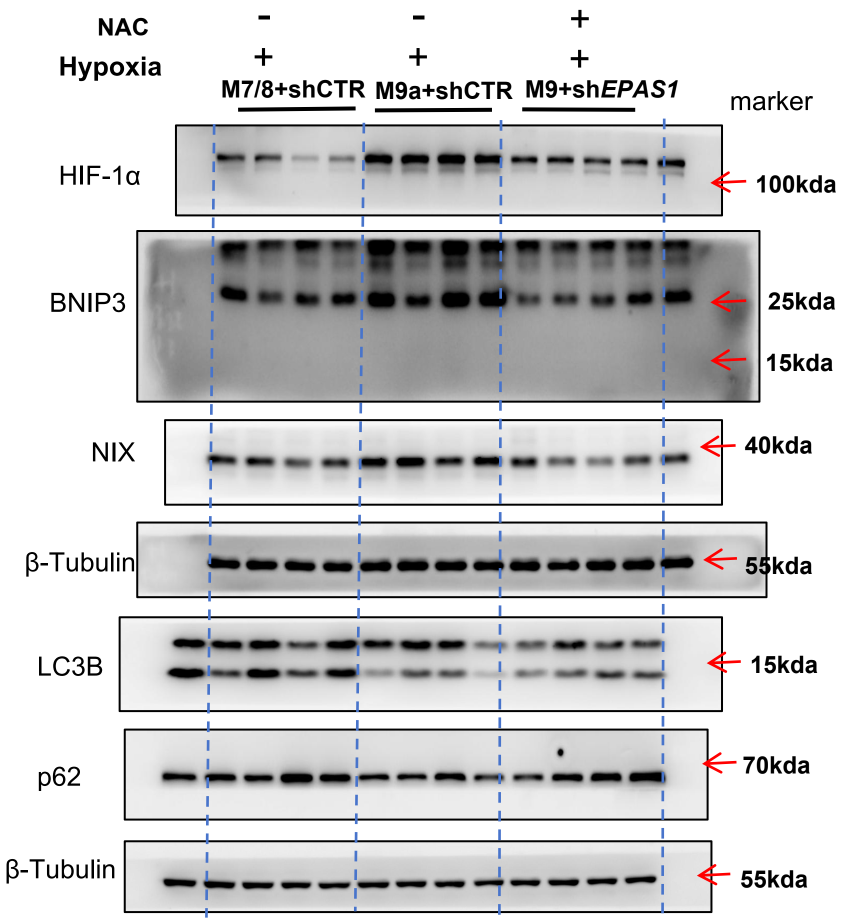

Figure 5I

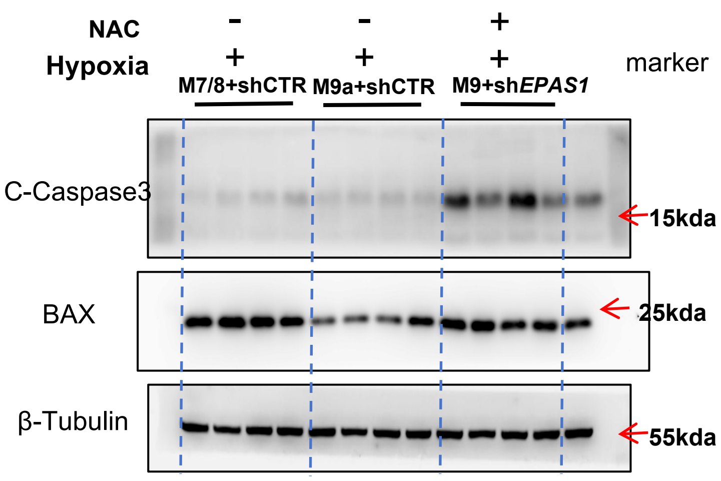

Figure 5K

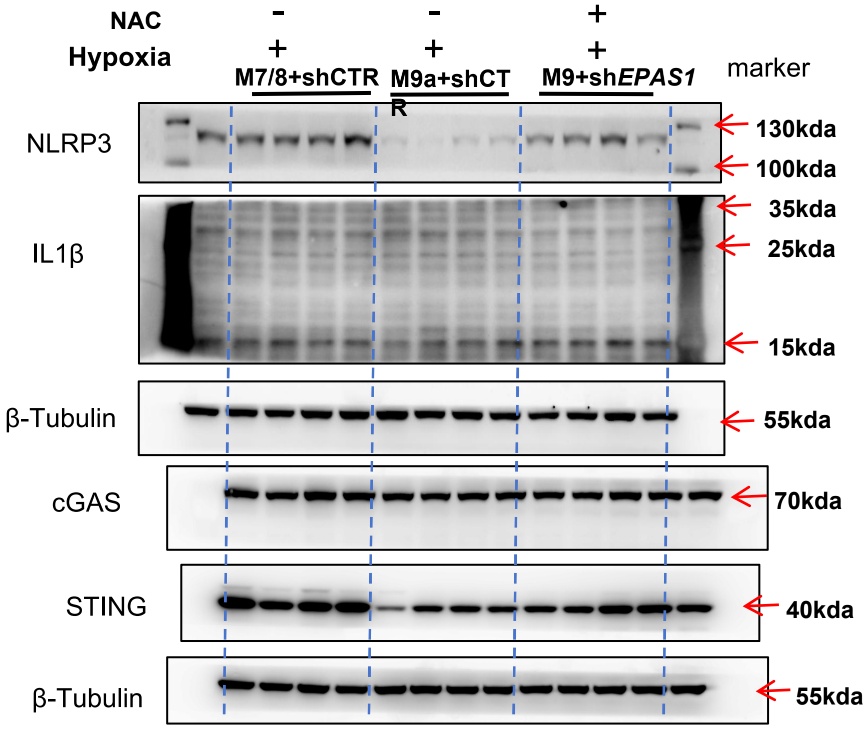

Figure 6

Figure 6B

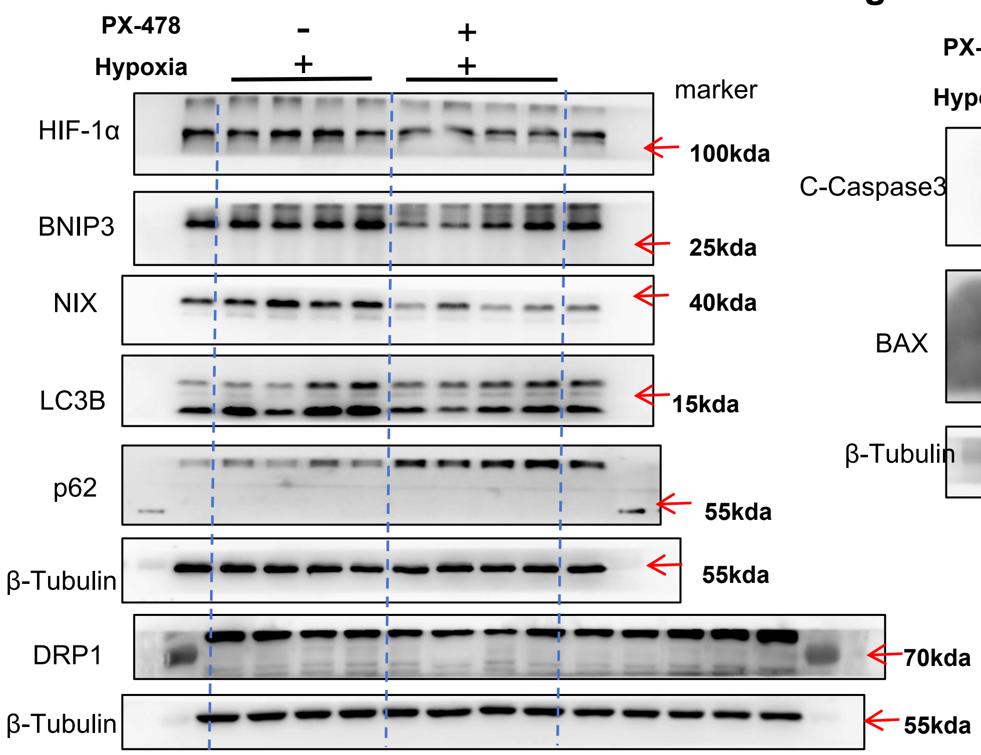

Figure 6I

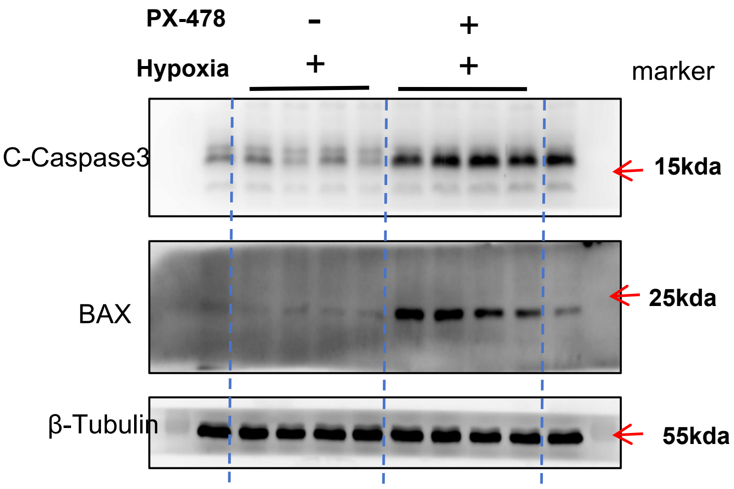

Figure 6K

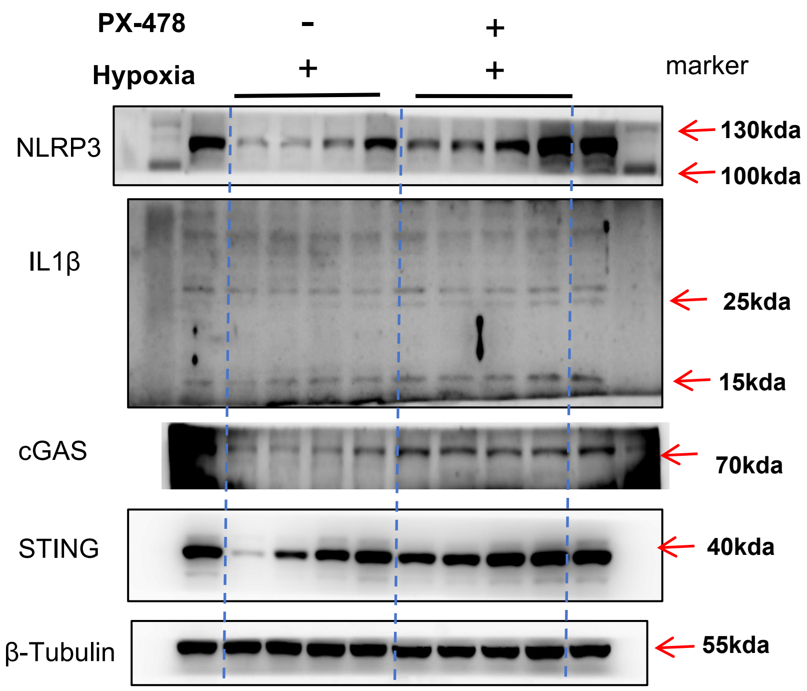

Figure 7

Figure 7B

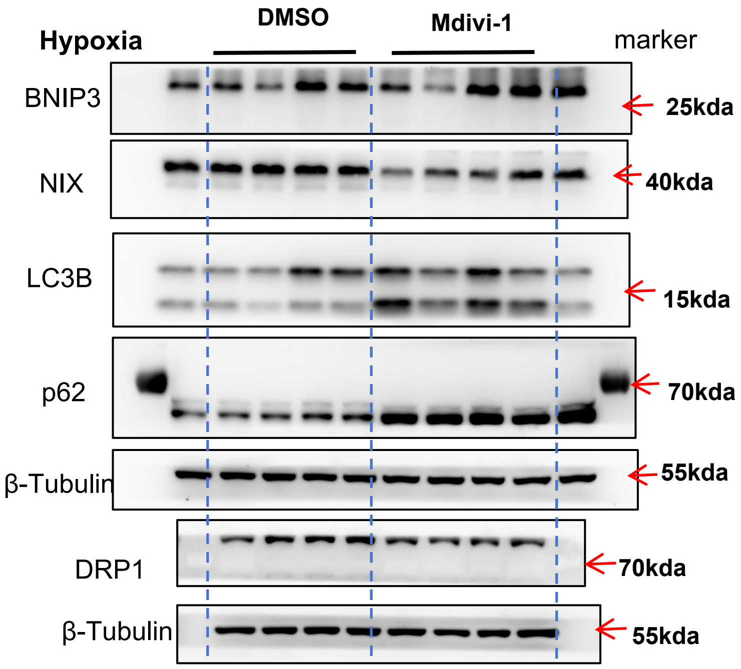

Figure 7I

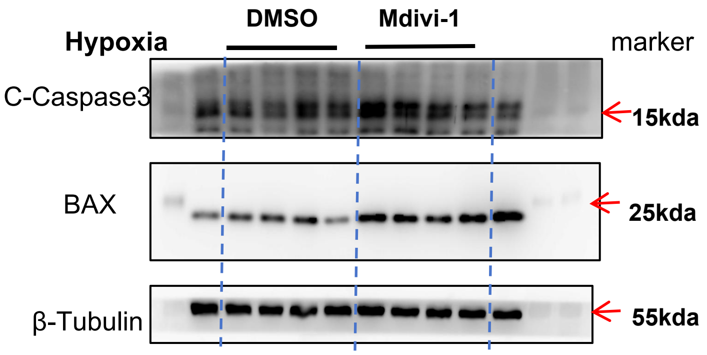

Figure 7K

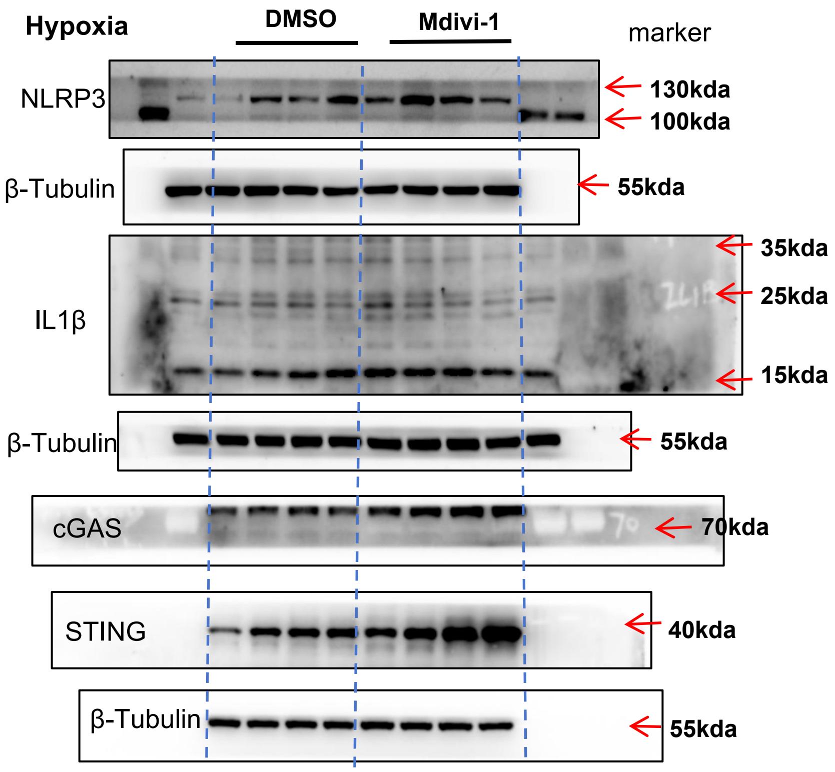

Figure S1-S6

Figure S1

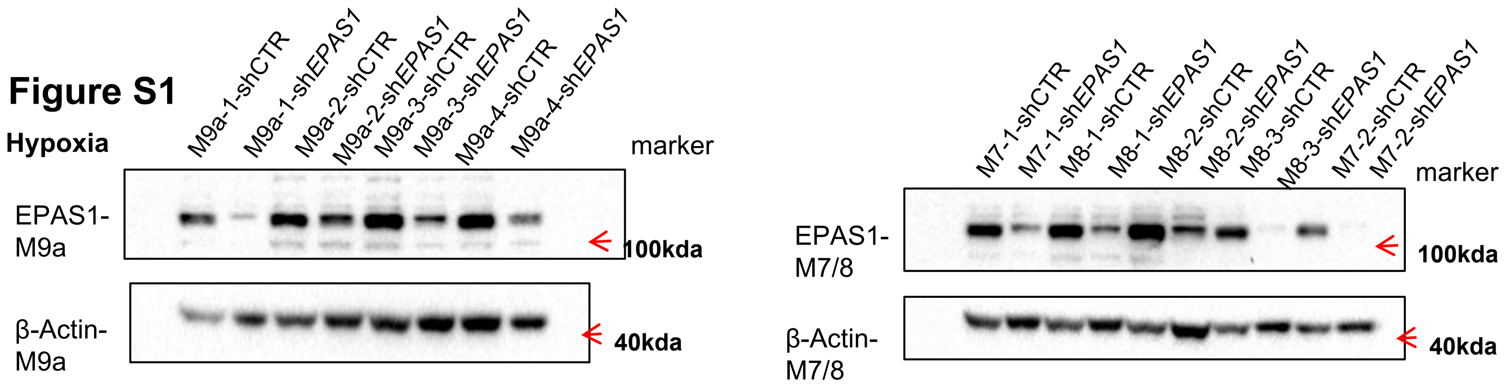

Figure S3A

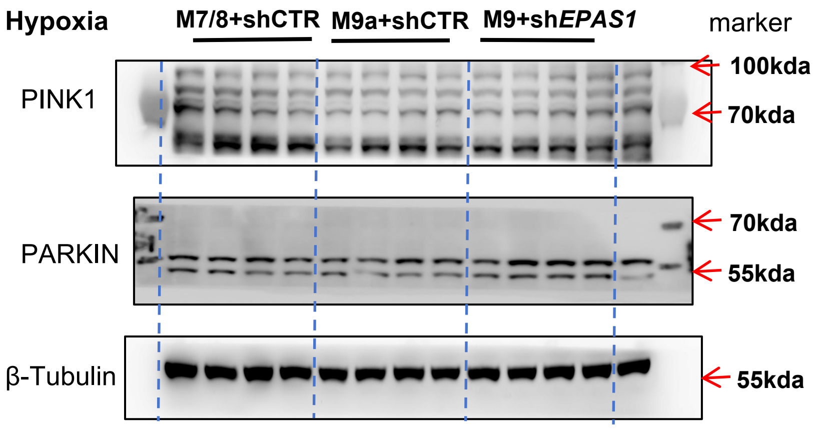

Figure S5A

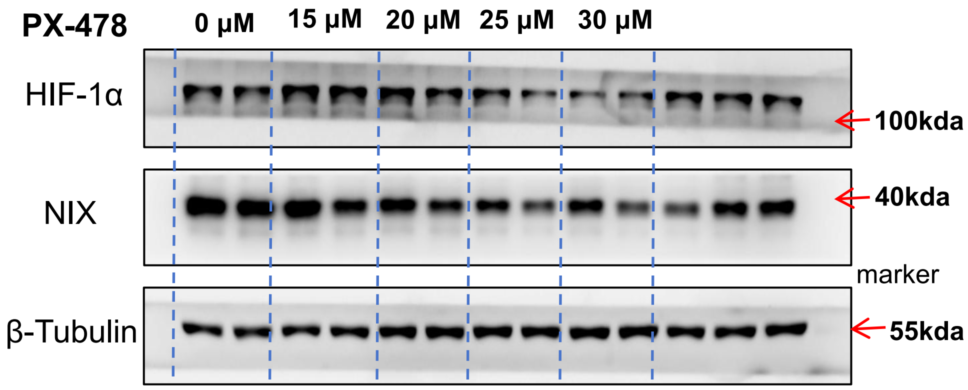

Figure S5B

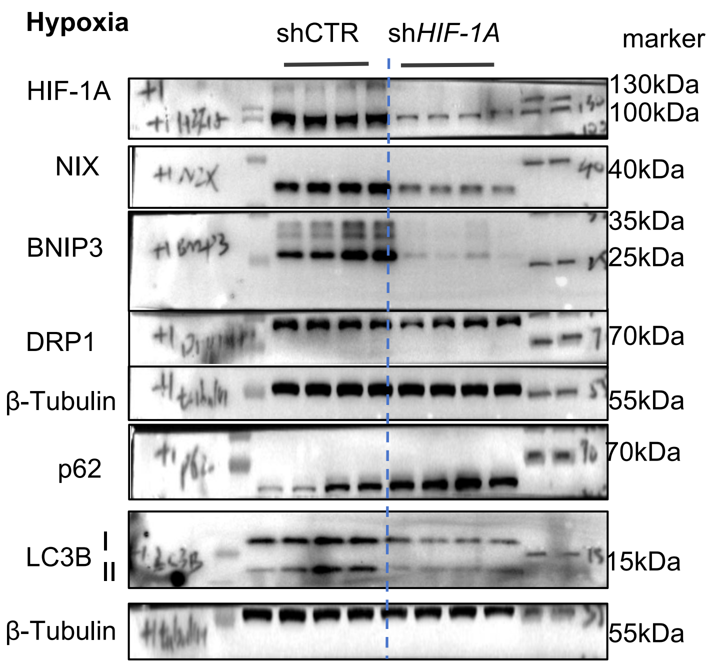

Figure S5C

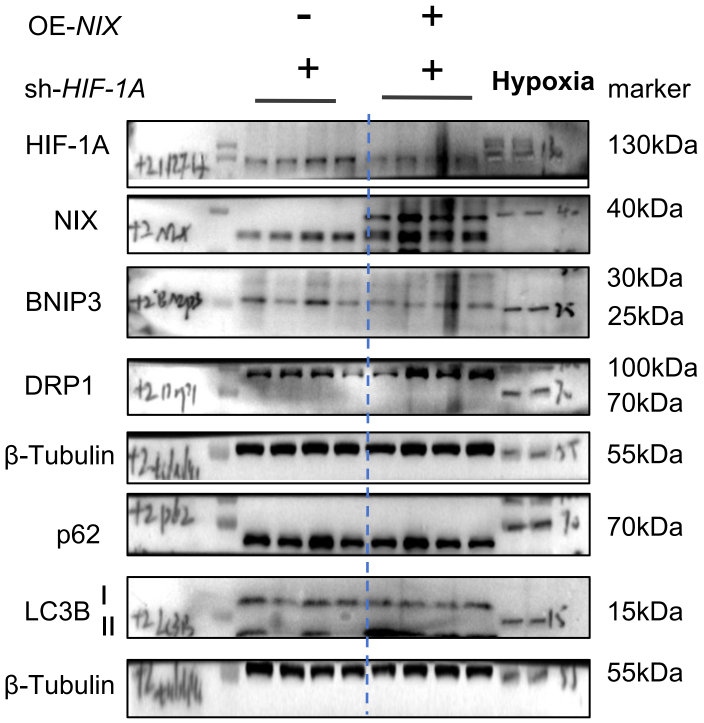

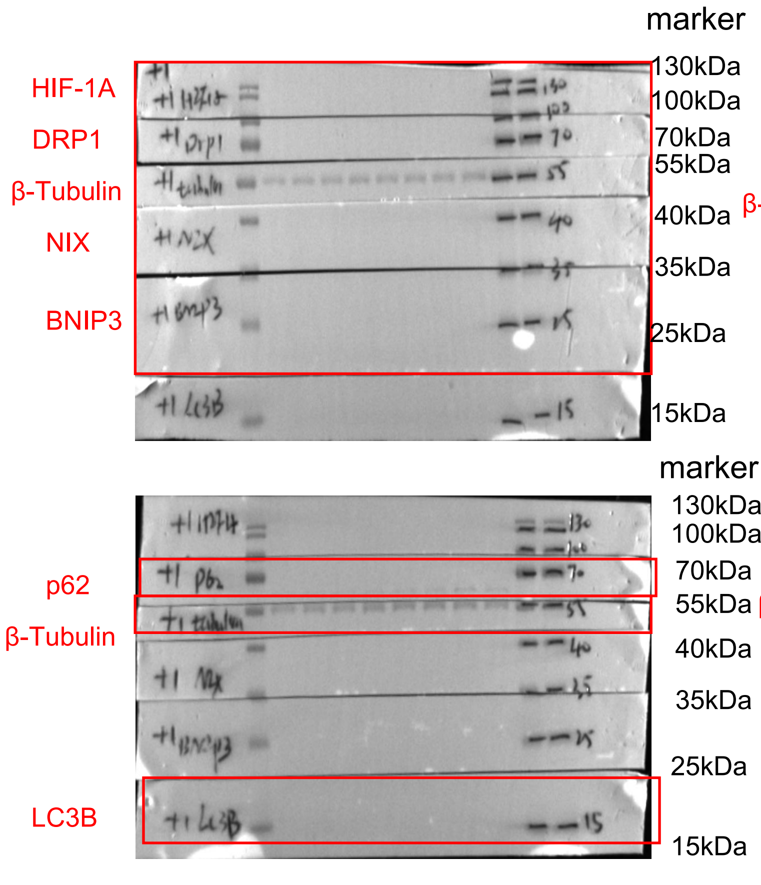

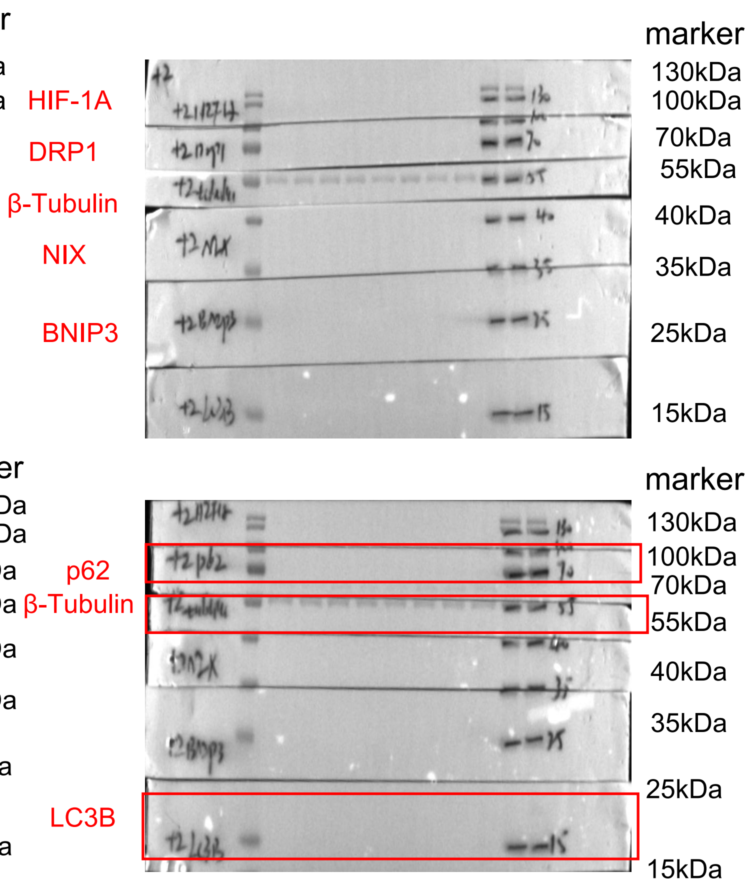

Figure S6A

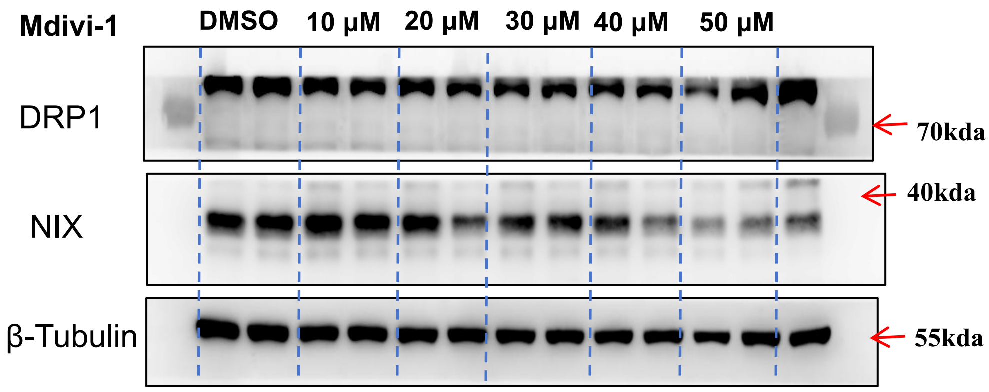

Full-membrane Western blots depicting biologically independent replicates from main figures, with molecular weight markers indicated.

Figure 2K

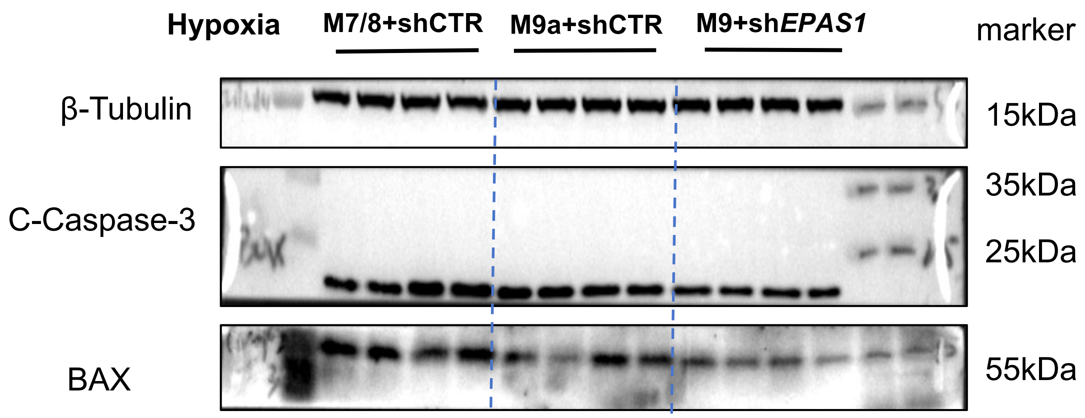
Hypoxia
M7/8+shCTR
M9a+shCTR
M9+sh*EPAS1*
marker
β-Tubulin
C-Caspase-3
BAX
15kDa
35kDa
25kDa
55kDa

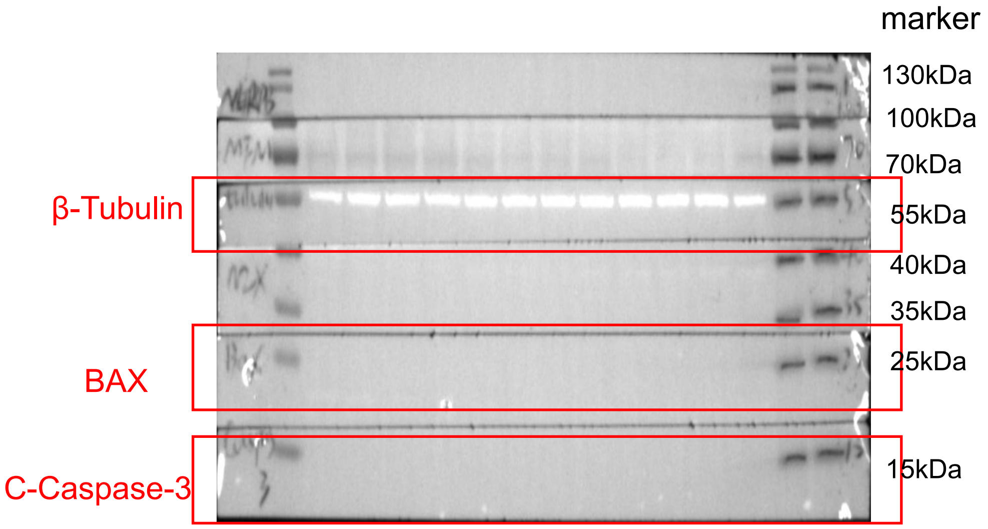
marker
130kDa
100kDa
70kDa
55kDa
40kDa
35kDa
25kDa
15kDa
β-Tubulin
BAX
C-Caspase-3

Figure 4A

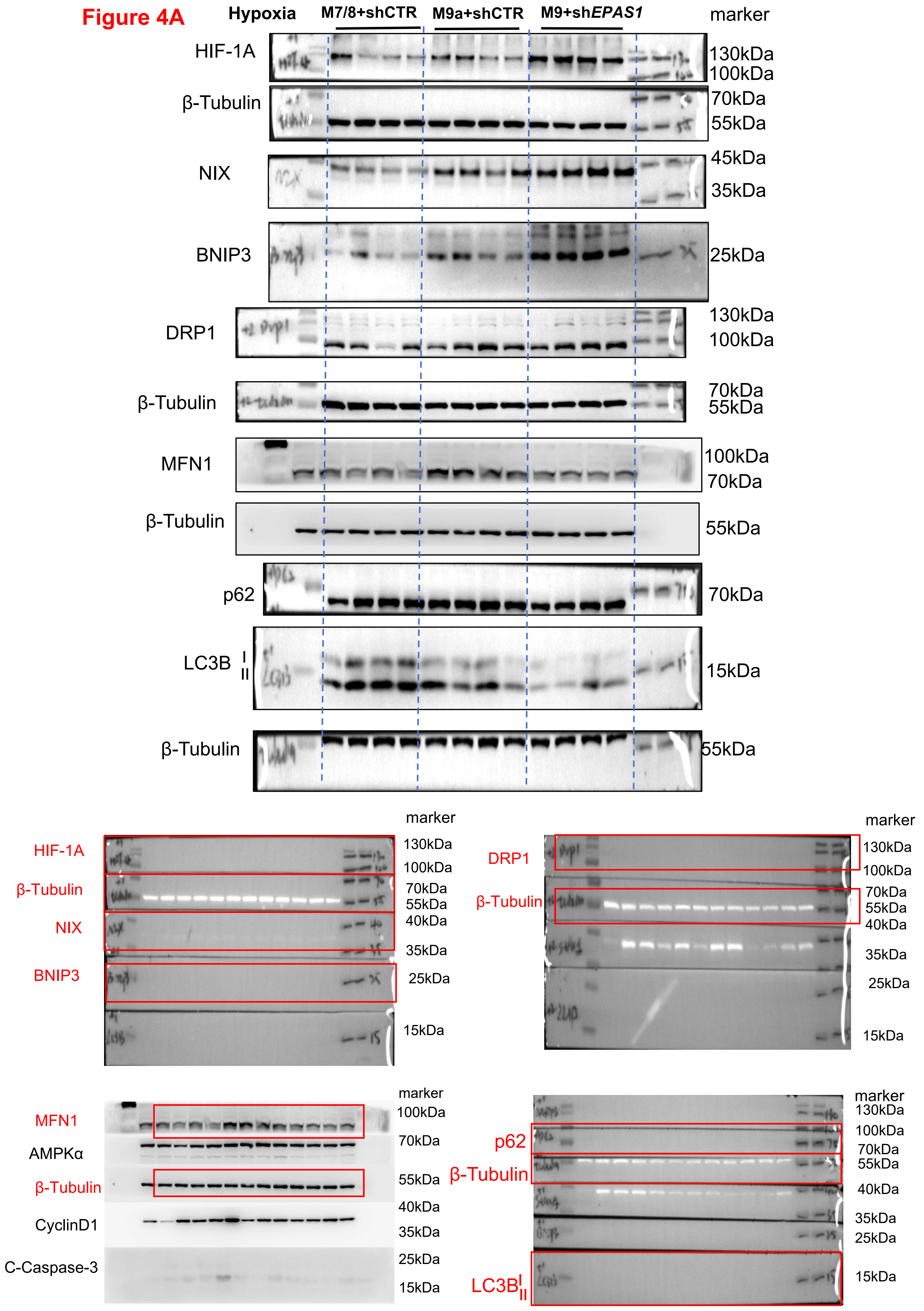

Hypoxia
M7/8+shCTR
M9a+shCTR
M9+shEPAS1
marker
HIF-1A
β-Tubulin
NIX
BNIP3
DRP1
β-Tubulin
MFN1
β-Tubulin
p62
LC3B I II
β-Tubulin
130kDa
100kDa
70kDa
55kDa
45kDa
35kDa
25kDa
130kDa
100kDa
70kDa
55kDa
100kDa
70kDa
55kDa
70kDa
15kDa
55kDa
marker
HIF-1A
β-Tubulin
NIX
BNIP3
130kDa
100kDa
70kDa
55kDa
40kDa
35kDa
25kDa
15kDa
marker
DRP1
β-Tubulin
130kDa
100kDa
70kDa
55kDa
40kDa
35kDa
25kDa
15kDa
marker
MFN1
AMPKα
β-Tubulin
CyclinD1
C-Caspase-3
100kDa
70kDa
55kDa
40kDa
35kDa
25kDa
15kDa
marker
p62
β-Tubulin
LC3B I II
130kDa
100kDa
70kDa
55kDa
40kDa
35kDa
25kDa
15kDa

Figure 4F

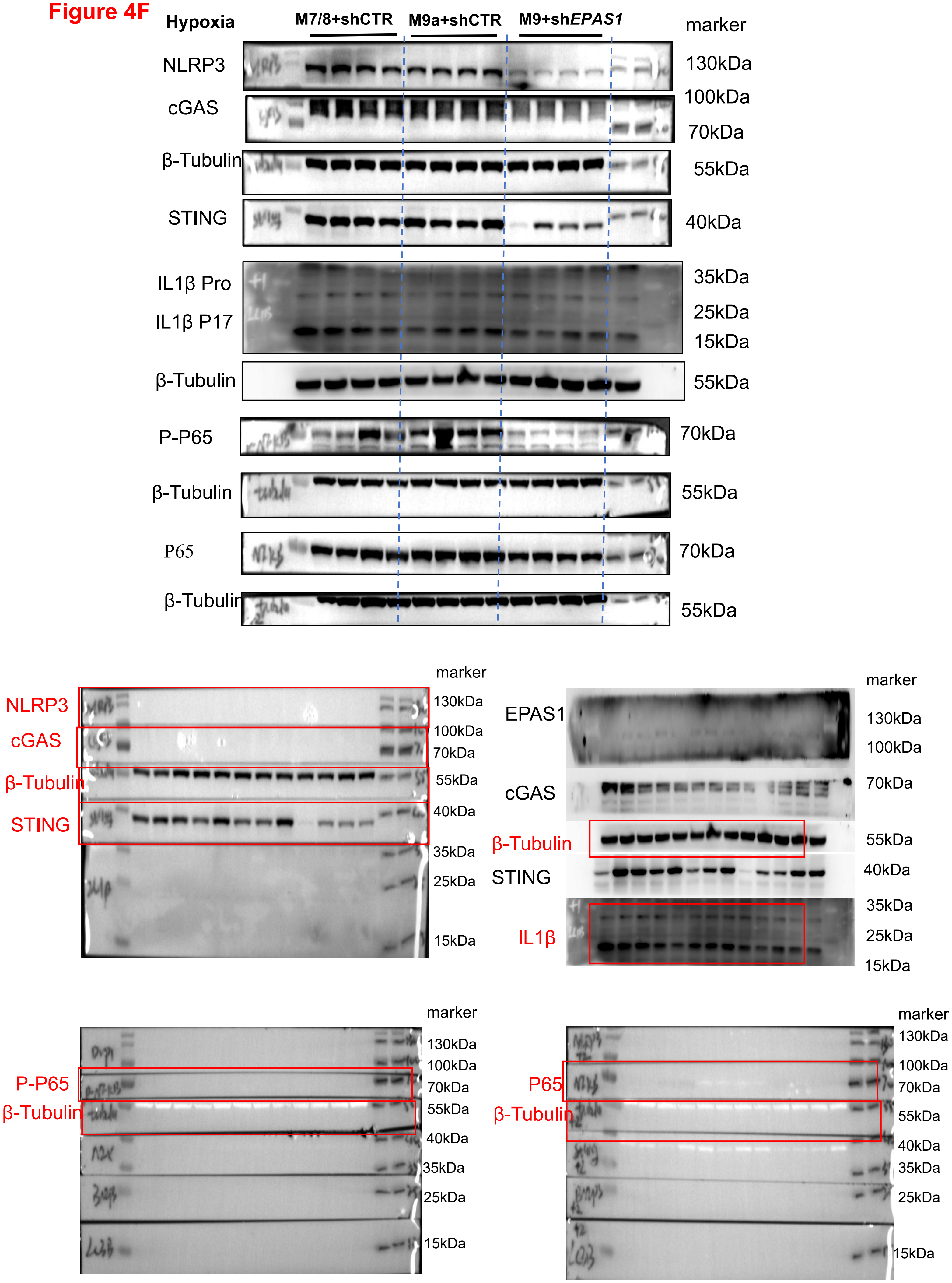
Hypoxia
M7/8+shCTR
M9a+shCTR
M9+shEPAS1
marker
NLRP3
cGAS
β-Tubulin
STING
IL1β Pro
IL1β P17
β-Tubulin
P-P65
β-Tubulin
P65
β-Tubulin
130kDa
100kDa
70kDa
55kDa
40kDa
35kDa
25kDa
15kDa
55kDa
70kDa
55kDa
70kDa
55kDa
marker
NLRP3
cGAS
β-Tubulin
STING
130kDa
100kDa
70kDa
55kDa
40kDa
35kDa
25kDa
15kDa
marker
EPAS1
cGAS
β-Tubulin
STING
IL1β
130kDa
100kDa
70kDa
55kDa
40kDa
35kDa
25kDa
15kDa
marker
P-P65
β-Tubulin
130kDa
100kDa
70kDa
55kDa
40kDa
35kDa
25kDa
15kDa
marker
P65
β-Tubulin
130kDa
100kDa
70kDa
55kDa
40kDa
35kDa
25kDa
15kDa

Figure 5B

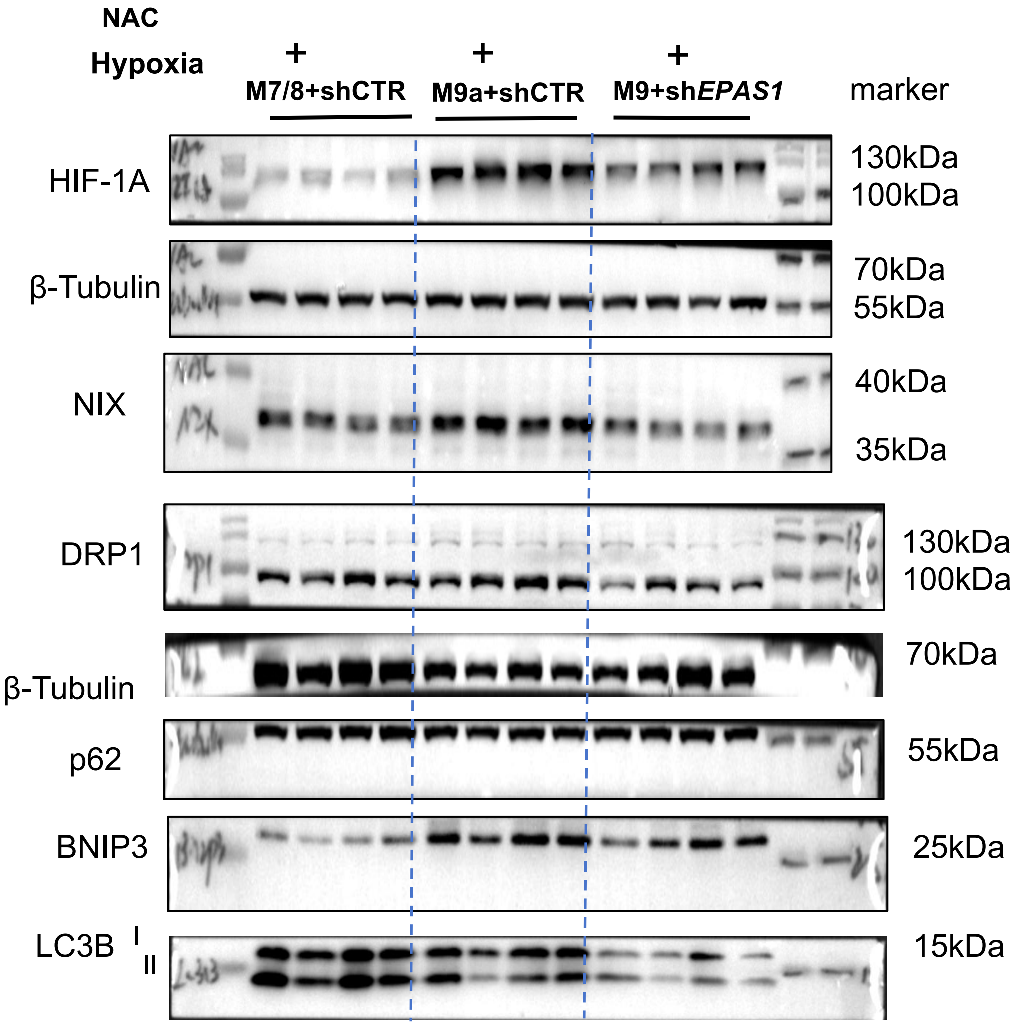

NAC
Hypoxia
+
+
+
M7/8+shCTR
M9a+shCTR
M9+sh*EPAS1*
marker
HIF-1A
130kDa
100kDa
β-Tubulin
70kDa
55kDa
NIX
40kDa
35kDa
DRP1
130kDa
100kDa
β-Tubulin
70kDa
p62
55kDa
BNIP3
25kDa
LC3B
I
II
15kDa

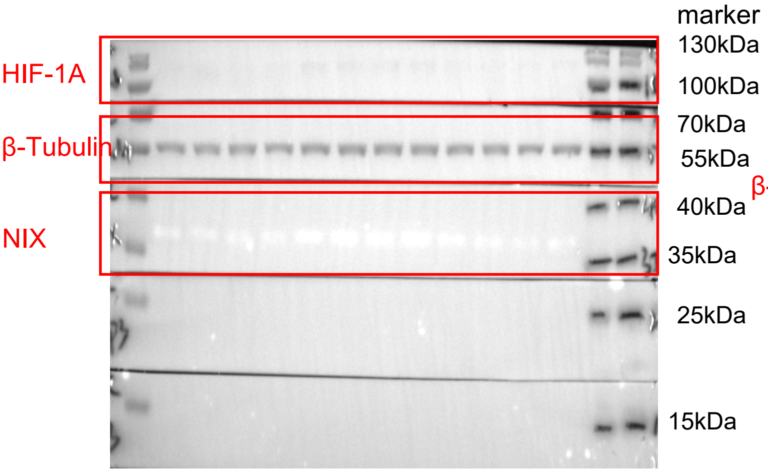

marker
HIF-1A
130kDa
100kDa
β-Tubulin
70kDa
55kDa
NIX
40kDa
35kDa
25kDa
15kDa

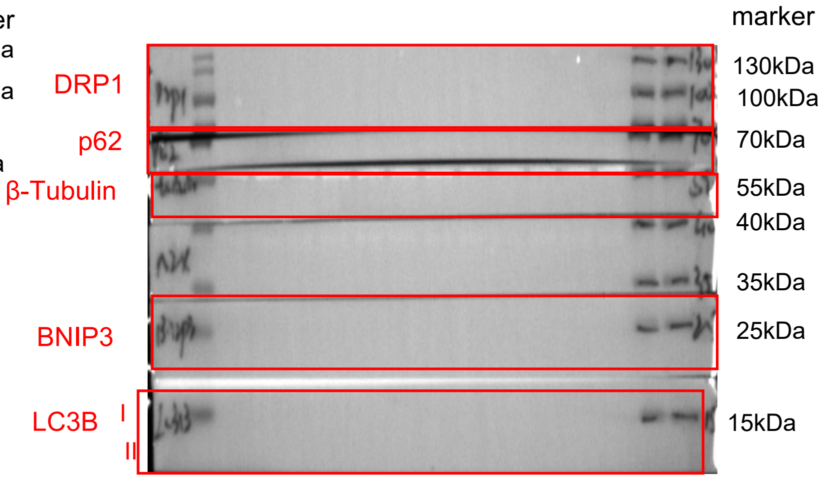

marker
DRP1
130kDa
100kDa
p62
70kDa
β-Tubulin
55kDa
40kDa
35kDa
BNIP3
25kDa
LC3B
I
II
15kDa

Figure 5I

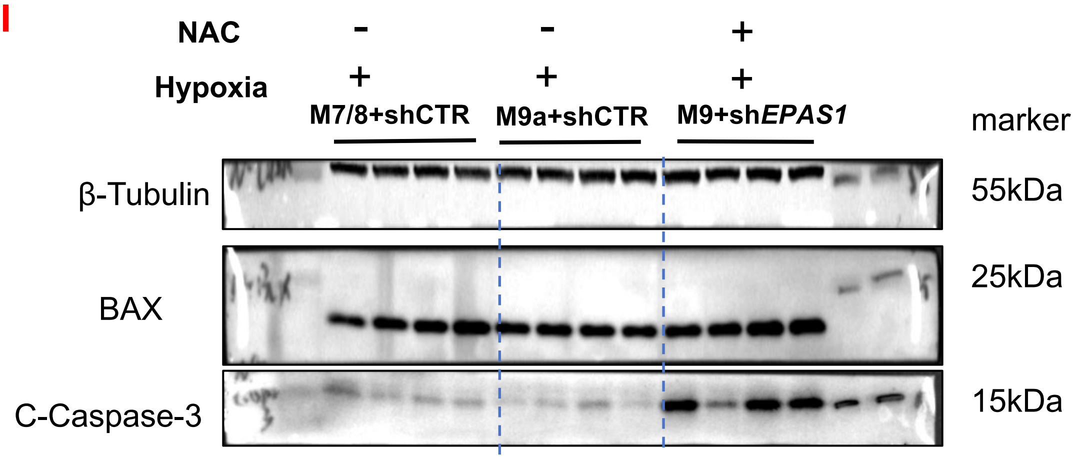

Figure 5K

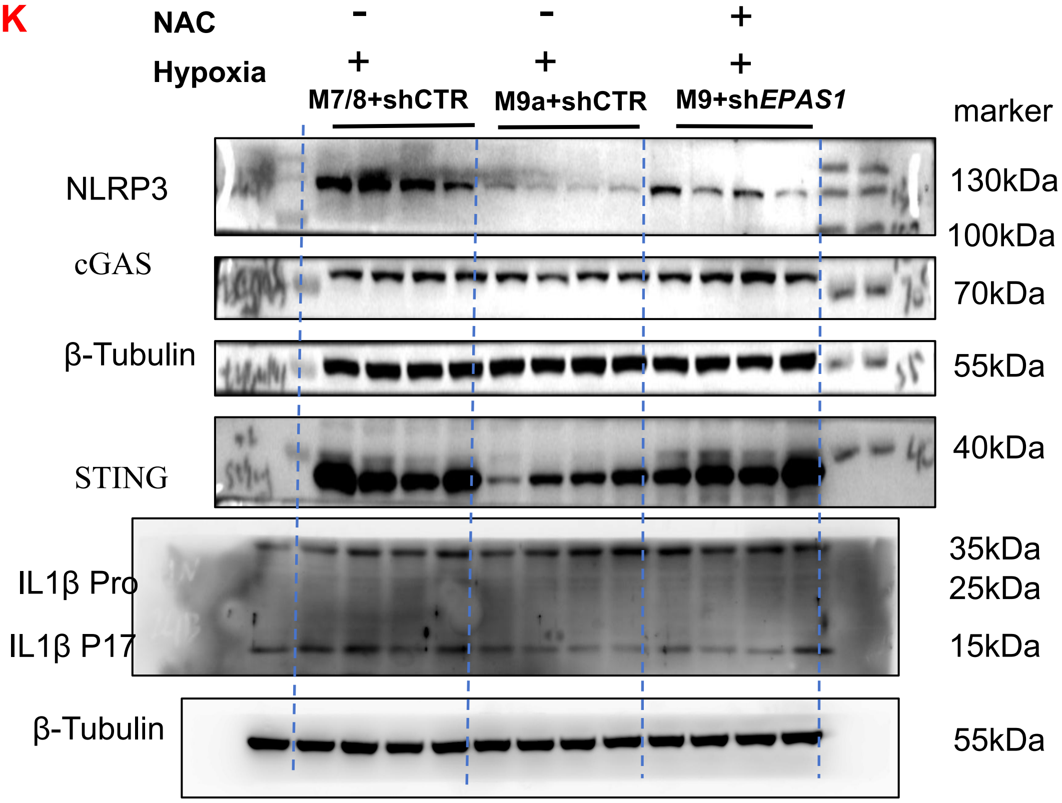

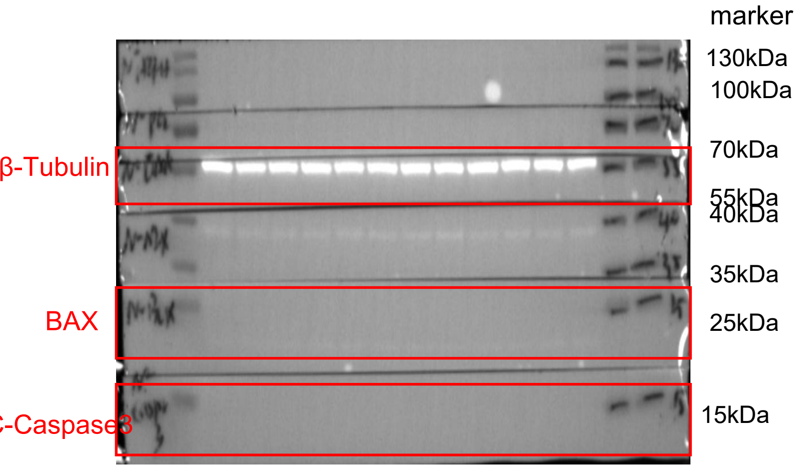

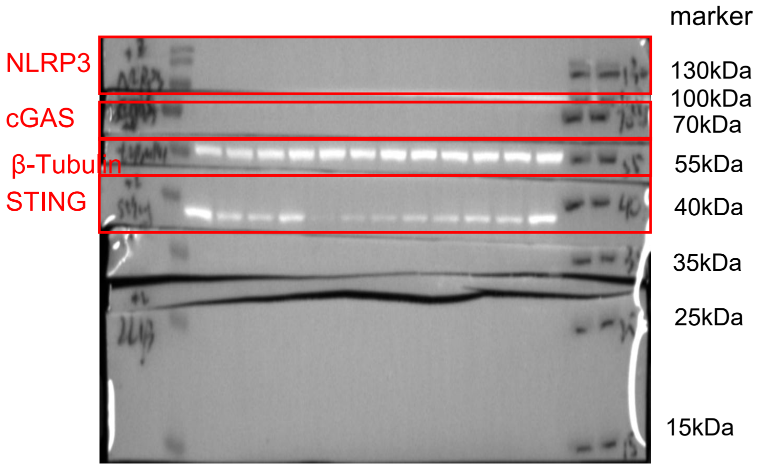

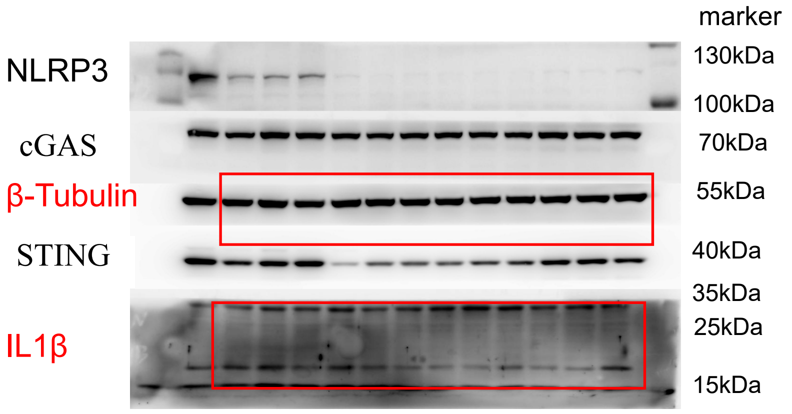

Figure 6B

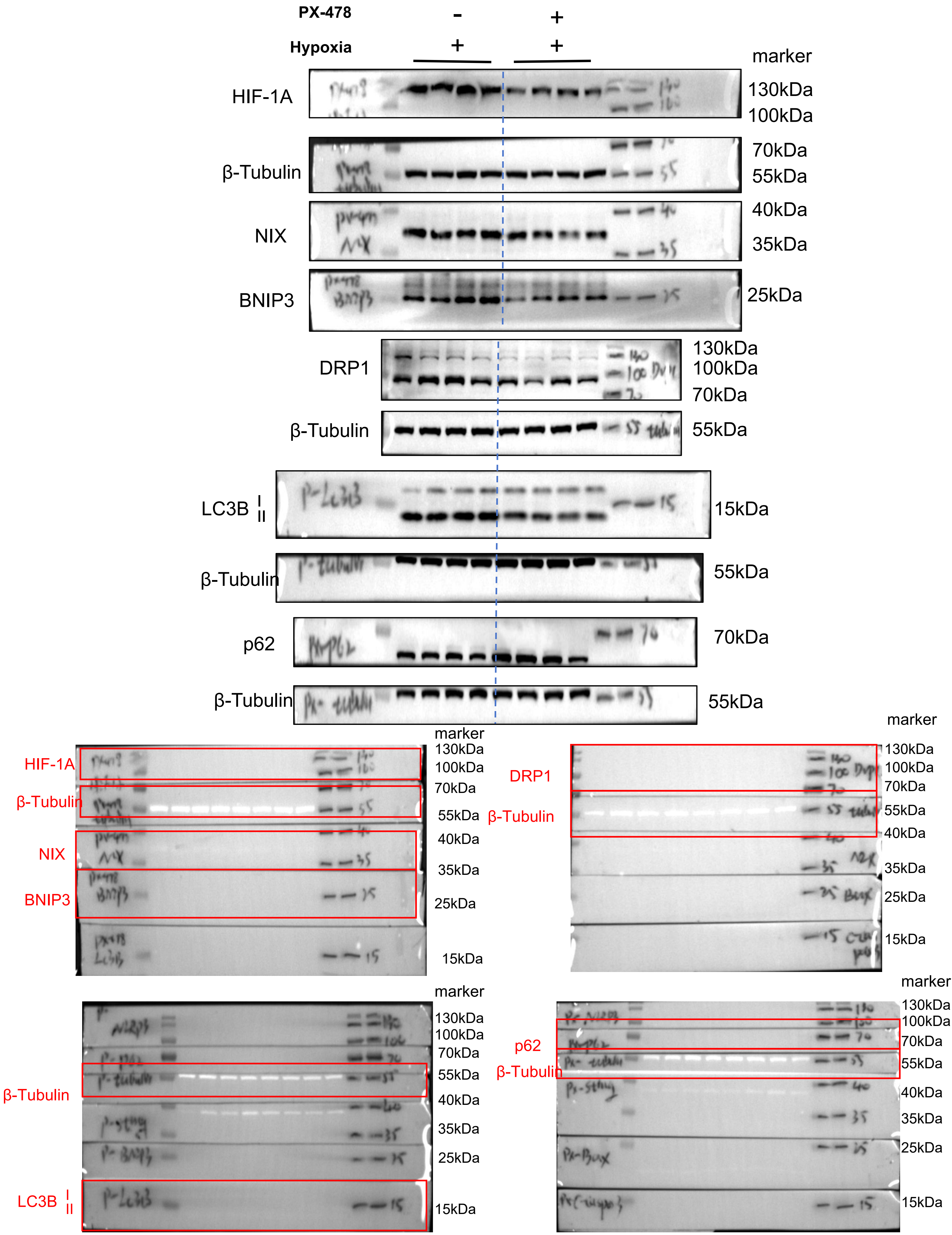

PX-478
Hypoxia
-
+
+
+
marker
HIF-1A
130kDa
100kDa
β-Tubulin
70kDa
55kDa
NIX
40kDa
35kDa
BNIP3
25kDa
DRP1
130kDa
100kDa
70kDa
β-Tubulin
55kDa
LC3B I II
15kDa
β-Tubulin
55kDa
p62
70kDa
β-Tubulin
55kDa
HIF-1A
β-Tubulin
NIX
BNIP3
marker
130kDa
100kDa
70kDa
55kDa
40kDa
35kDa
25kDa
15kDa
DRP1
β-Tubulin
marker
130kDa
100kDa
70kDa
55kDa
40kDa
35kDa
25kDa
15kDa
β-Tubulin
LC3B I II
marker
130kDa
100kDa
70kDa
55kDa
40kDa
35kDa
25kDa
15kDa
p62
β-Tubulin
marker
130kDa
100kDa
70kDa
55kDa
40kDa
35kDa
25kDa
15kDa

Figure 6I

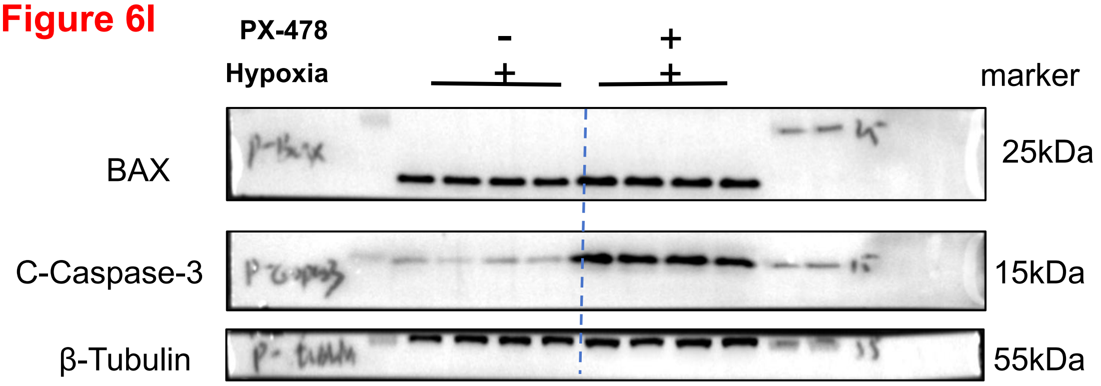

Figure 6K

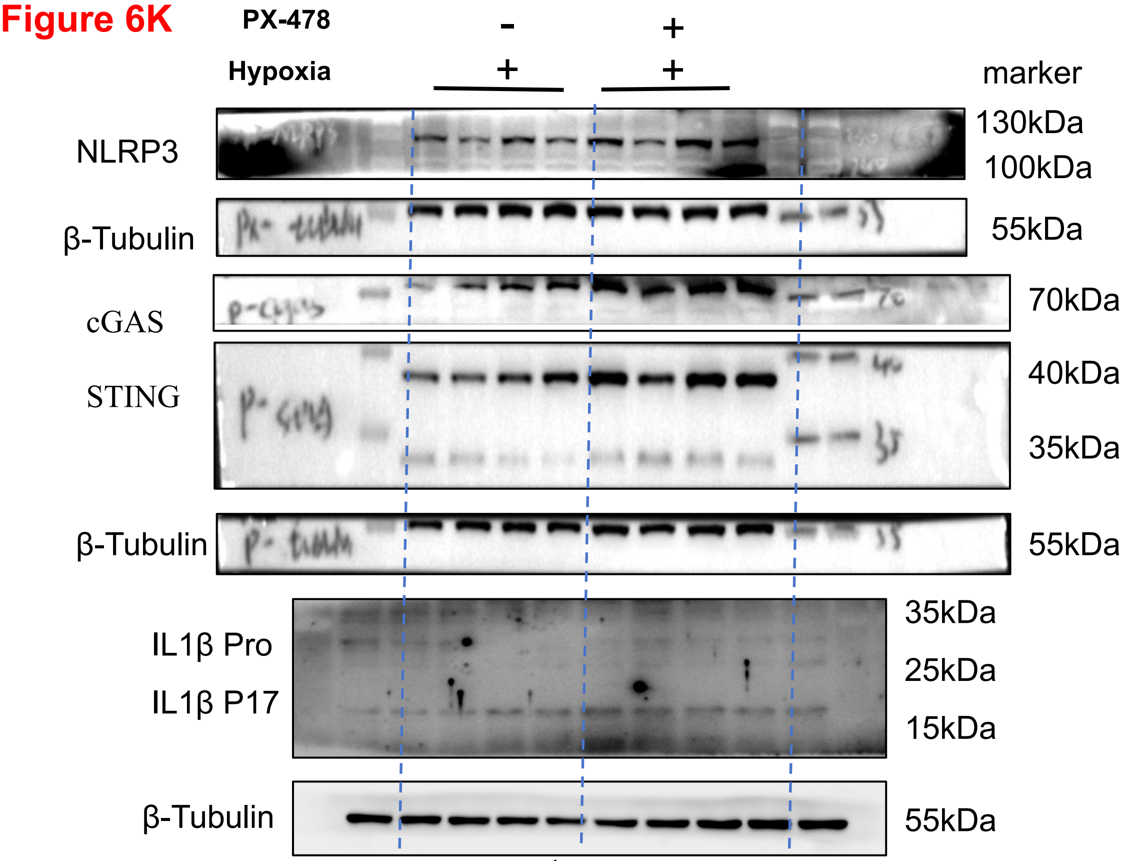

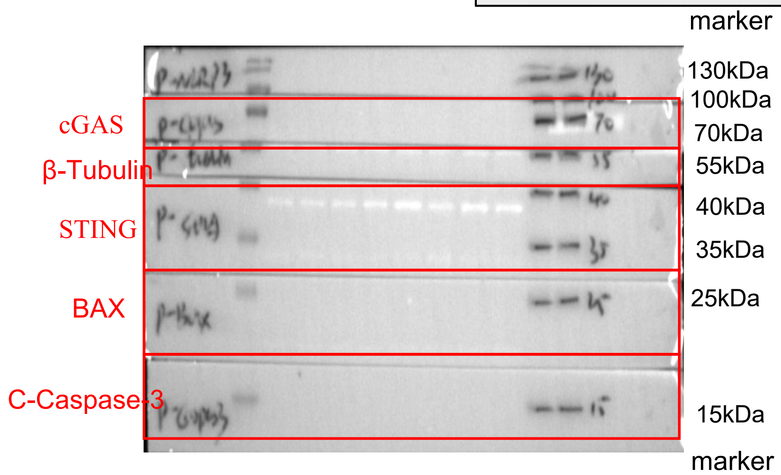

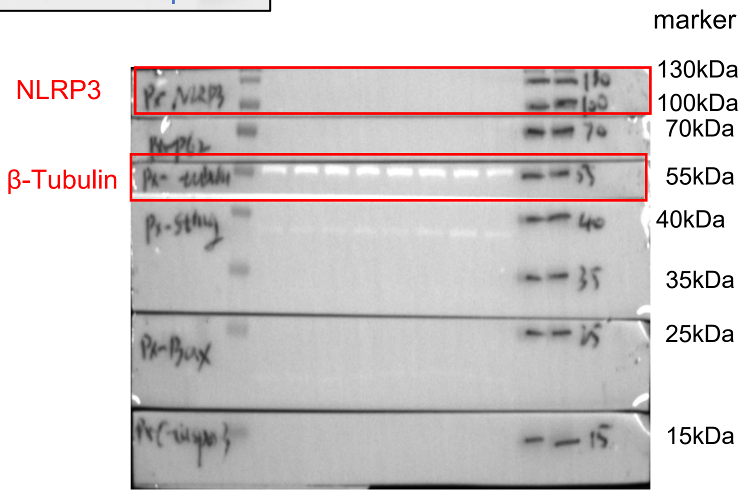

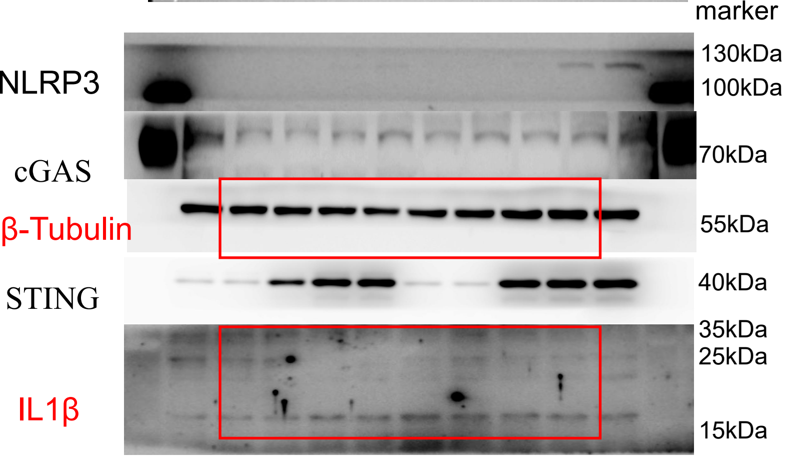

Figure 7A

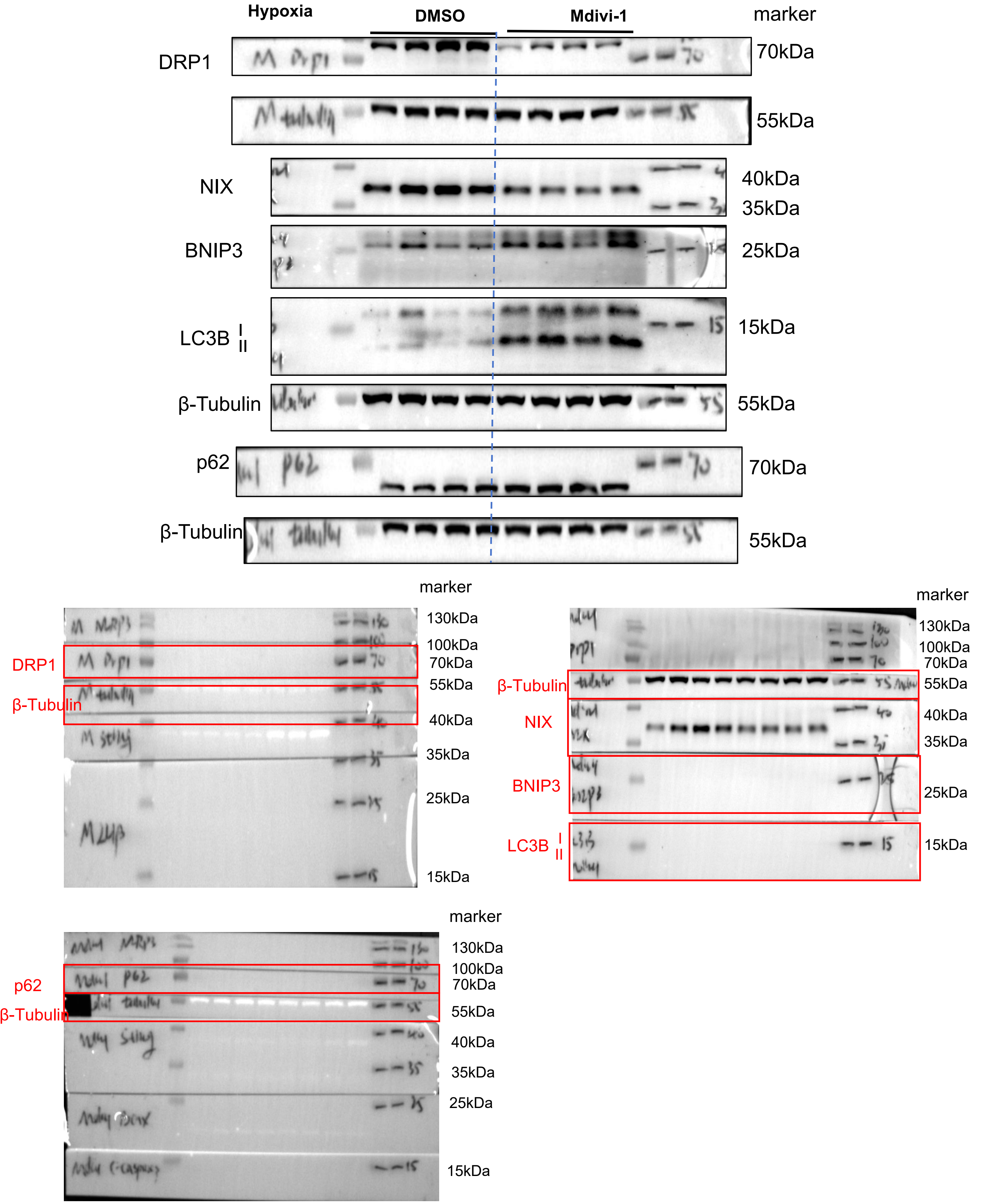
Hypoxia
DMSO
Mdivi-1
marker
DRP1
70kDa
55kDa
NIX
40kDa
35kDa
BNIP3
25kDa
LC3B
I
II
15kDa
β-Tubulin
55kDa
p62
70kDa
β-Tubulin
55kDa
marker
130kDa
100kDa
70kDa
55kDa
40kDa
35kDa
25kDa
15kDa
DRP1
β-Tubulin
marker
130kDa
100kDa
70kDa
55kDa
40kDa
35kDa
25kDa
15kDa
β-Tubulin
NIX
BNIP3
LC3B
I
II
marker
130kDa
100kDa
70kDa
55kDa
40kDa
35kDa
25kDa
15kDa
p62
β-Tubulin

Figure 7I

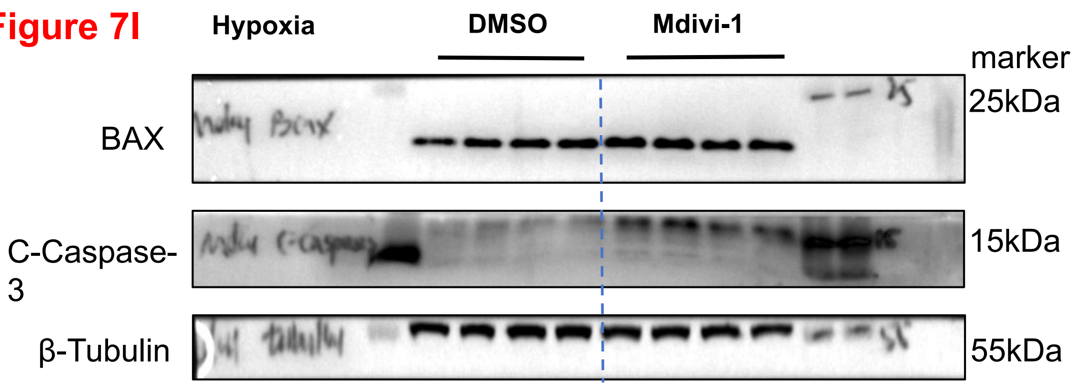

Figure 7K

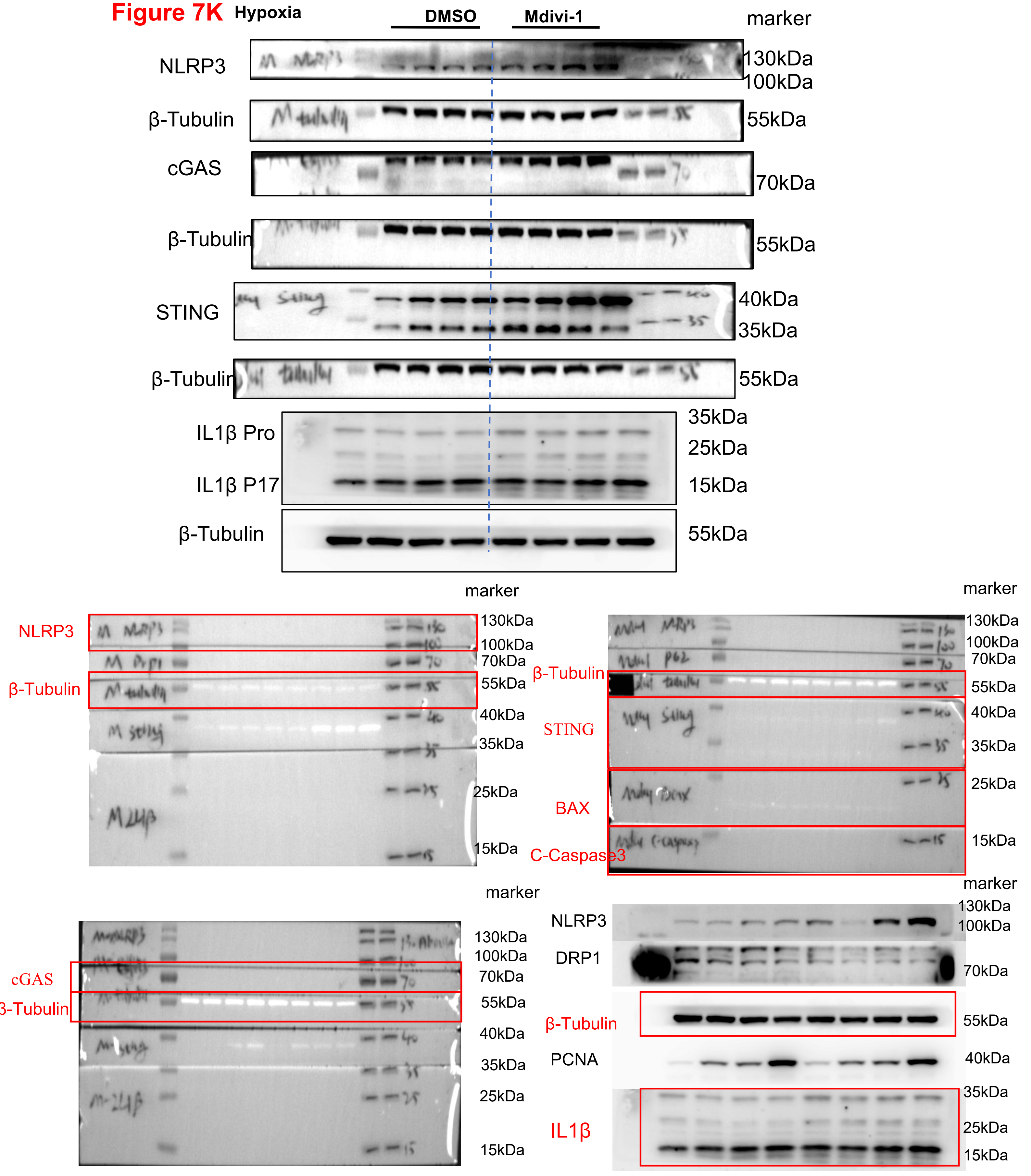

Supplement: Supplementary file 2 — Full length uncropped original western blots. [file 41420_2025_2933_MOESM2_ESM.pdf]
